# Genomic Instability of the Sex-Determining Locus in Atlantic Salmon (*Salmo salar*)

**DOI:** 10.1534/g3.115.020115

**Published:** 2015-09-22

**Authors:** Krzysztof P. Lubieniecki, Song Lin, Emily I. Cabana, Jieying Li, Yvonne Y. Y. Lai, William S. Davidson

**Affiliations:** Department of Molecular Biology and Biochemistry, Simon Fraser University, Burnaby, British Columbia, V5A 1S6 Canada

**Keywords:** sex-determining locus, sdY, jumping gene, Atlantic salmon, genetics of sex

## Abstract

Atlantic salmon and rainbow trout, like other members of the subfamily Salmoninae, are gonochoristic with male heterogamety. The finding that sex-linked genetic markers varied between species suggested that the sex-determining gene differs among salmonid species, or that there is one sex-determining gene that has the capacity to move around the genome. The discovery of *sdY*, the sex-determining gene in rainbow trout, and its presence in many male salmonids gave support to the latter. Additional evidence for a salmonid-specific, sex-determining jumping gene came from the mapping of the sex-determining locus to three different chromosomes in Tasmanian male Atlantic salmon lineages. To characterize the sex-determining region, we isolated three *sdY* containing BACs from an Atlantic salmon male library. Sequencing of these BACs yielded two contigs, one of which contained the sdY gene. Sequence analysis of the borders of male-specific and female/male common regions revealed highly repetitive sequences associated with mobile elements, which may allow an sdY cassette to jump around the genome. FISH analysis using a BAC or a plasmid containing the sdY gene showed that the sdY gene did indeed localize to the chromosomes where *SEX* had been mapped in different Tasmanian Atlantic salmon families. Moreover, the plasmid sdY gene probe hybridized primarily to one of the sex chromosomes as would be expected of a male-specific gene. Our results suggest that a common salmonid sex-determining gene (sdY) can move between three specific loci on chromosomes 2, 3, and 6, giving the impression that there are multiple SEX loci both within and between salmonid species.

Fish in the subfamily Salmoninae, such as Atlantic salmon (*Salmo salar*) and rainbow trout (*Oncorhynchus mykiss*), are generally considered to be gonochoristic and to exhibit male heterogamety (*i.e.*, sex is determined by an XY/XX system) (Thorgaard 1977, 1978; Devlin and Nagahama 2002; Davidson *et al.* 2009). This is supported by the observation that the progeny of a hormonally sex-reversed female (neo-male) and a normal female are all female (Johnstone and MacLachlan 1994; Johnston *et al.* 1999). However, there is some evidence to suggest that sex differentiation in Atlantic salmon is thermolabile (King *et al.* 2012), and high temperature during development was shown to increase the masculinization rate (Valdivia *et al.* 2014) in an all-female (XX) rainbow trout population carrying the *mal* mutation (Quillet *et al.* 2002). It should be noted that the nature of the *mal* mutation is unknown; it has been identified as a spontaneous male phenotype in what was expected to be an all female XX strain of rainbow trout.

Several microsatellite genetic markers were identified as being sex-linked in Atlantic salmon, brown trout (*Salmo trutta)*, rainbow trout, or Arctic charr (*Salvelinus alpinus*), but the markers linked to *SEX* (male phenotype) in one species are not linked to *SEX* in different species; rather, they reside on autosomes (Woram *et al.* 2003; Davidson *et al.* 2009). This led to the suggestion that there were either several sex-determining genes in salmonids or a single sex-determining gene that could move around the genome by transposition or translocation of a small chromosome arm (Woram *et al.* 2003; Davidson *et al.* 2009). The discovery of the master sex-determining gene in rainbow trout, *sdY* (Yano *et al.* 2012), and its presence in males of many other salmonid species, including Atlantic salmon (Yano *et al.* 2013), was interpreted by these authors as providing support for the latter hypothesis of a universal salmonid sex determination “jumping gene.” Further support for the jumping gene hypothesis comes from the mapping of the sex-determining locus to three different chromosomes in Tasmanian Atlantic salmon in a male lineage–dependent manner (Eisbrenner *et al.* 2014). If, however, *sdY* is not the universal sex-determining gene in salmonids, then one would not expect to find *sdY* located at the same place as *SEX* in the genome of all male salmonids. Indeed, Eisbrenner *et al.* (2014) discovered a few cases in which the sdY gene is present in female and absent in male Atlantic salmon. This phenomenon of phenotypic males lacking *sdY* and some phenotypic females possessing the sdY gene has also been described in Chinook salmon (Cavileer *et al.* 2015). Therefore, it appears that there may be more to the story of sex determination in salmonids than just the presence of the sdY gene.

Although the Atlantic salmon genome has been sequenced by the International Collaboration to Sequence the Atlantic Salmon Genome (Davidson *et al.* 2010), and although an assembly has been deposited in GenBank (http://www.ncbi.nlm.nih.gov/assembly/GCA_000233375.4/#/st), the specimen chosen for sequencing was a double-haploid female (nicknamed Sally). If *sdY* is indeed the sex-determining gene in Atlantic salmon and its presence is restricted to males, then this region of the genome will not be obtained as part of this sequencing project. Therefore, we set out to isolate Bacterial Artificial Chromosome clones (BACs) from an Atlantic salmon library (CHORI-214), which was constructed from the DNA from a single Norwegian Atlantic salmon male (Ng *et al.* 2005; Thorsen *et al.* 2005), and to characterize the genomic environment of the sdY gene.

A preliminary account of this work was presented at the Plant and Animal Genome Conference (PAG XXIV) by Palibroda *et al.* (2013). Faber-Hammond *et al.* (2015) also sequenced the same three *sdY*-containing Atlantic salmon BAC clones and compared these sequences with the corresponding regions of rainbow trout and Chinook salmon. Our results are in agreement with their conclusions.

## Materials and Methods

### Screening the CHORI-214 Atlantic salmon genomic library for BACs containing *sdY*

Two oligonucleotide probes, which were designed from sequences of salmonid sdY cDNAs (Supporting Information, Table S1), were used to screen the CHORI-214 Atlantic salmon BAC library (Ng *et al.* 2005; Thorsen *et al.* 2005). The probe labeling and the hybridization were performed as described by Johnstone *et al.* (2009). BAC filters were prehybridized in a solution containing 5× SSC, 5× Denhardt’s reagent, and 0.5% SDS at 65° for 3 hr prior to the addition of the ^32^P-labeled probes, and the mixture was incubated overnight. The filters were washed three times, with 1 hr per wash with the washing solution (0.1% SDS and 1× SSC) at 65°. The filters were then wrapped in Saran® wrap before being placed in a phosphor screen cassette for exposure overnight. A Typhoon Imaging System was used to visualize the image of the screen. PCR with sdY-specific primers for exon 2 and exon 4 (Table S1 and Figure S1) was performed on crude extracts of cultures of the hybridization-positive BAC clones. The PCR conditions comprised 95° for 5 min followed by 35 cycles of 95° for 45 sec, 65° for 45 sec, and 72° for 2 min, and then 72° for 10 min. Only BAC clones that were positive by both hybridization and gene-specific PCR were considered to contain the sdY gene.

### Production of BAC shotgun libraries

The production of shotgun libraries of BACs that were hybridization-positive and PCR-positive for sdY was performed essentially as previously described by Quinn *et al.* (2010). BAC DNA was first isolated using a Large Construct Kit (Qiagen, Mississauga, ON), and a minimum of 5 μg of purified BAC DNA was sheared by sonication to yield 2–5 kb DNA fragments. The sheared DNA was end-repaired (Epicentre End-It DNA End-Repair Kit) and purified from a 1% agarose gel using a QIAquick Gel Extraction Kit (Qiagen, Mississauga, ON). Sheared/end-repaired BAC DNA (100 ng), 25 ng of *Sma*I digested and shrimp alkaline phosphatase-treated pUC19, and 1 unit of T4 DNA ligase (Invitrogen, Burlington, ON) in a 20-μl ligation reaction were incubated at 14° overnight. Three μl of the ligation mix was used to transform *Escherichia coli* XL1-Blue Supercompetent cells (Stratagene, La Jolla, CA). Approximately 3000 colonies containing hybrid recombinant plasmids for each BAC were sent to the Michael Smith Genome Sciences Centre (Vancouver, BC) for paired-end Sanger sequencing.

### Assembly and annotation of Atlantic salmon BAC sequences containing sdY

The Sanger paired-end sequences from ∼3000 colonies from each of the three *sdY*-containing BACs were assembled independently using Phred/Phrap (Ewing and Green 1998; Ewing *et al.* 1998) to produce contigs derived from a single BAC. The resulting contigs were viewed using Consed (Gordon *et al.* 1998). The contig consensus sequences were annotated using the Genomics Research on Atlantic Salmon Project (GRASP) annotation pipeline (see http://trutta.mbb.sfu.ca/bacannotations/GRASPbac.html Information for a schematic of the work flow). Alignments of contig sequences from each BAC were performed using Geneious 6.0.6 (Biomatters).

### Linkage analysis

We were curious to know if the *sdY* from the European Atlantic salmon male used to produce the CHORI-214 BAC library (Thorsen *et al.* 2005) had *SEX* on linkage group 1 (chromosome Ssa02), as had been found by Woram *et al.* (2003). Therefore, we searched for a variable microsatellite that could be used in a genetic mapping analysis. A microsatellite in contig 1 of the assembly of the Atlantic salmon BACs containing the sdY gene was identified using FastPCR (www.biocenter.helsinki.fi/bi/programs/fastpcr.htm), and a pair of primers was designed to amplify the microsatellite (Table S1). The M13 sequence, 5′-TGTAAAACGACGGCCAGT-3′, was added to the 5′ end of the forward primer as part of the genotyping procedure (Danzmann *et al.* 2008). The primers were first tested on DNA from the parents of the two Atlantic salmon SALMAP families, Br5 and Br6 (Danzmann *et al.* 2008), to determine if any of the parents were heterozygous for the marker. Both parents in the Br5 family were informative; therefore, the microsatellite marker was genotyped in this family using an ABI 377 automated DNA sequencer. The genotype data were added to the data for family Br5 from Danzmann *et al.* (2008), and linkage analysis was performed using the LINKMFEX package (Danzmann and Gharbi 2001).

### Fluorescent *in situ* hybridization of Atlantic salmon chromosomes

Fluorescent *in situ* hybridization (FISH) analyses using BAC DNA as the probes were performed using a modification of the procedure of Li *et al.* (2011) as described by Brenna-Hansen *et al.* (2012). Up to 2 ml of blood was aseptically drawn from the caudal vein of the fish using a sterile syringe inserted near the anal fin. The blood was collected into a Vacutainer tube containing heparin, gently mixed, and transported to the lab at 4°. The heparinized blood was thoroughly mixed with 5 ml of media L-15 (Gibco) in a 15-ml sterile plastic tube and placed on ice for 5 min. The diluted blood was then centrifuged at 1200 rpm for 5 min at room temperature. After centrifugation, the buffy coat (containing lymphocytes) above the red blood cells was floated in plasma by gentle stirring with a 1-ml pipette. The lymphocyte-enriched plasma was then collected in a new 15-ml sterile plastic tube. The lymphocyte-enriched plasma was centrifuged at 1500 rpm for 5 min, and the resulting cell pellet was suspended in 5 ml of complete media L-15 containing 10% fetal bovine serum (FBS), 60 μg/ml of kanamycin sulfate, 1× antibiotic/antimycotic solution (100 units/ml of penicillin, 100 μg/ml of streptomycin, and 250 ng of amphotericin B), 25 μM of 2-mercaptoethanol, 18 μg/ml of phytohemagglutinin (PHA-W), and 100 μg/ml of lipopolysaccharide (LPS). The cells were cultured at 18° in a culture tube slanting to an angle of approximately 30° with gentle daily mixing for 6 d. Approximately 90 min before cell harvest, the lymphocyte culture was supplemented with 500 ng/ml colcemid. The cells were collected by centrifugation at 1500 rpm for 5 min, and the supernatant was discarded. The cell pellet was suspended in 2 ml of 0.075 M KCl hypotonic solution for 20 min at 20°. The hypotonic solution was slowly added to a volume of 2 ml. Then, 2 ml of fresh Carnoy’s fixative (3 methanol: 1 acetic acid) was added slowly. After centrifugation at 1500 rpm for 5 min, the supernatant was discarded. The fixed cells were gently suspended in 3 ml of Carnoy’s fixative. The fixation step was repeated two more times, and then the cells were suspended in 1–2 ml of Carnoy’s fixative. A microscope slide was exposed to hot water vapor at 73.5° for 30 sec. The cell suspension was immediately dropped onto the slide. After the slide surface became “grainy” the slide was immediately exposed again to the hot water vapor at 73.5° for 30 sec. The slide was then quickly dried on a hot surface, which provided good chromosome spreading.

BAC DNA, prepared using a large construct kit (QIAgen), was labeled with either SpectrumOrange (Vysis) or SpectrumGreen (Vysis); 500 ng of extracted BAC DNA was mixed with 1.25 μl of SpectrumOrange or SpectrumGreen, 2.5 μl of 0.1 mM dTTP, 5 μl 0.1 mM dNTP mix, 2.5 μl 10× nick translation buffer, and 2.5 μl of nick translation enzyme in 25 μl. The reaction mixture was briefly mixed and centrifuged, and then incubated in a PCR thermocycler at 15° for 8 hr, followed by 70° for 10 min, and then paused at 4°; 5 μl of the nick translation reaction mixture of the BAC containing the sdY gene and 5 μl of the nick translation reaction mixture of a chromosome-specific BAC (Phillips *et al.* 2009) were combined with 2 μg of Atlantic salmon Cot-1 DNA and 2 μg of human placental DNA in a microcentrifuge tube; 0.1 volume of 3 M sodium acetate and 2.5 volumes of 95% EtOH were added to precipitate the DNA. The mixture was incubated at −80° for 60 min and then centrifuged at 12,000 rpm for 30 min at 4° to pellet the DNA. The supernatant was removed and the pellet was dried for 15 min at room temperature. The pellet was then suspended in 3 μl of distilled water and 7 μl of hybridization buffer by shaking at 250 rpm for 30 min at 37°. The probe was denatured by heating at 80° for 5 min and then chilled on ice for 1 min. Then, the probe was incubated at 37° from 30 to 60 min prior to hybridization.

Hybridization of the fluorescent-labeled DNA probe with chromosome spreads was performed as suggested by the manufacture (Vysis). The freshly made metaphase-containing slides were treated with 2× SSC for 30 min at 37°, and then serial dehydrated in 70% EtOH, 85% EtOH, and 100% EtOH, with each treatment lasting 2 min. The hybridization area was marked using a tipped scribe. The slide was denatured in 70% formamide in 2× SSC, pH 7.0–8.0, at 73° for 3 min. Then, the slide was serial dehydrated in −20° 70% EtOH, 5% EtOH, and 100% EtOH, with each treatment being 2 min, and then air-dried; 10 μl denatured probe was added to the slide, and a coverslip was immediately applied and sealed with rubber cement. The slide was put in a sealed humidified box at 37° for 16 hr. The coverslip was removed together with the rubber cement seal, and the slide was immediately placed into a 0.4× SSC/0.3% NP-40 wash solution at 73° for 4 min, with several agitations per minute. The slide was then treated with the 2× SSC/0.1% NP-40 wash solution at room temperature for 2 min. The slide was dried in the dark, and then 10 μL of DAPI antifade solution (Invitrogen) was applied to the slide. A multiphoton confocal microscope A1R MP (Nikon) was used to check the metaphase spreads. Laser 405 was used to detect the DAPI stain, laser 488 was used for the green dUTP labeling, and laser 560 was used for the orange dUTP labeling.

FISH analyses using a plasmid containing the Atlantic salmon sdY gene (PCR amplification product obtained using primers from contig 2: Contig 2 FISH 858F and Contig 2 FISH 5976R, Table S1) as the probe required a more elaborate procedure than that for BAC DNA. The plasmid was first labeled using a DIG-nick translation kit (Roche) at 15° for 70 min to obtain labeled fragments ranging in size from 200 to 400 bp. The probe mixture for visualizing the location of the sdY gene contained 50 ng of DIG-nick translated plasmid probe and 100 ng of a SpectrumOrange (Vysis) labeled BAC for a particular chromosome (Phillips *et al.* 2009). Ethanol precipitation of the probe mixture and slide preparation were performed as described for BAC FISH with the modification of air-drying slides for 72 hr at 55° before denaturing the chromosomes. The precipitated probe mixture was then dissolved in 10 μl of a solution containing 50% deionized formamide, 2× SSC, 10% dextran sulfate, 50 mM sodium phosphate, pH 7, before hybridization with chromosome spreads overnight. The coverslip was removed together with the rubber cement seal and the slide was immediately placed into a 2× SSC wash solution containing 50% formamide at 45° for 2 min without agitation. The second wash was performed with immersion in a TNT buffer containing 100 mM Tris-HCl, pH 7.5, 150 mM NaCl, and 0.05% Tween 20 for 2 min. The slide was then incubated with 100 μl of TNB buffer containing 100 mM Tris-HCl, pH 7.5, 150 mM NaCl, and 0.5% blocking reagent for 30 min at 37° with a coverslip. The coverslip was then removed by immersion of the slide in TNT buffer for 30 sec. A series of antibody reactions was then performed by incubating 100 μl of each specific antibody for 30 min at 37° with a washing step for 3 min in TNT buffer between each of reactions. The first antibody was mouse monoclonal anti-DIG, 0.5 μg/ml in TNB (Roche Diagnostics, Laval, QC), followed by the second antibody, DIG-conjugated sheep antimouse IgG (Millipore (Canada) Ltd., Etobicoke, ON), 2 μg/ml in TNB, and the third antibody was fluorescein-conjugated sheep anti-DIG (Roche Diagnostics, Laval, QC), 2 μg/ml in TNB. The antibody attached slide was washed by TNT buffer for 2 min and then subjected to a series of dehydration steps of 70%, 90%, and 100% ethanol for 2 min each. The air-dried slide was finally counterstained with 10 μl of DAPI antifade solution (Life Technologies Inc., Burlington, ON) and sealed with a coverslip using rubber cement. Laser 405 was used to detect the DAPI stain, laser 488 was used for the DIG-labeled plasmid probe, and laser 560 was used for the SpectrumOrange-labeled BAC probe.

### Sequencing of the *sdY* gene from Atlantic salmon from Tasmania and brown trout

Various sections of the sdY gene region of Tasmanian Atlantic salmon and brown trout were first amplified through PCR using TopTaq DNA polymerase (Qiagen) and primers stated in Table S1 to achieve overlapping sequences. Purified PCR products were cloned using either the pSTBlue-1 Acceptor vector kit or pJET 1.2 vector kit. DNA was extracted from 5 ml LB culture of five single colonies picked from each transformation. Restriction digestion was used to confirm the existence of positive inserts in the transformed bacteria prior to the recombinant plasmids being sent for sequencing by Genewiz.

### Data availability

Atlantic salmon BACS from the CHORI-214 library may be obtained from The BACPAC Resource Center at the Children's Hospital Oakland Research Institute in Oakland, California, USA (https://bacpac.chori.org/). The Atlantic salmon *sdY*-containing plasmid cone is available upon request. Table S1 contains the sequences of all probes and primers used. Sequence data are available at GenBank and the accession numbers are listed within the text.

## Results

### Characterization of Atlantic Salmon BACs containing sdY

Three BACs (S0227M24, S0367C01, and S0524M13) from the CHORI-214 Atlantic salmon male BAC library (Ng *et al.* 2005; Thorsen *et al.* 2005) were identified as being hybridization-positive for oligonucleotide probes designed from sdY cDNA sequences (Table S1). These BACs also gave PCR amplification products when primers (Table S1 and Figure S1) designed from exon 2 and exon 4 were used. Neither S0227J24 nor S0367C01 belong to any fingerprint scaffold in the Atlantic salmon physical map, whereas S0524M13 is one of 19 BACs that comprise fingerprint scaffold 2599, which is estimated to span 282.6 kb (Ng *et al.* 2005; www.asalbase.org). None of the other BACs in fingerprint scaffold 2599 were positive for sdY, either by hybridization or by PCR screening.

**Figure 1:**
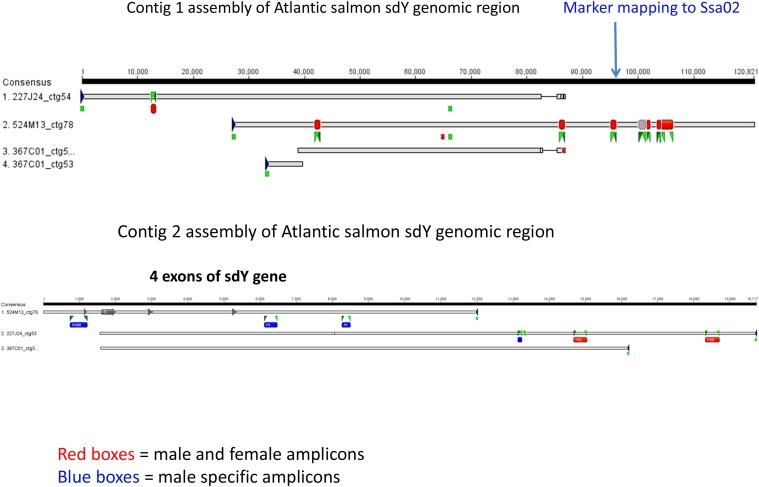
Schema of the assembly of the contig 1 and contig 2 with indication of the male-specific (blue boxes) and male and female common (red boxes) genomic regions. BAC end sequences are denoted by green boxes at the end of sequences. The green triangles illustrate the positions of primers (Table S1) used in PCR. The exons of *sdY* are shown as boxes ending in arrows in Contig 2. The position of the microsatellite marker that mapped to linkage group 1 (Ssa02) in European Atlantic salmon is shown by the blue arrow in Contig 1.

S0227M24, S0367C01, and S0524M13 were shotgun cloned and sequenced, and the sequence of each BAC was assembled individually. A considerable amount of repetitive sequence was evident from the CONSED view (data not shown); however, it was possible to combine the sequences from S0227M24, S0367C01, and S0524M13 into two contigs: one of ∼120 kb (contig 1; KP898411) and the other of ∼19 kb (contig 2; KP898412) (Figure 1). The orientation of the contigs relative to one another was determined from the BAC end sequences. We were not able to join contig 1 and contig 2 using primers and long-range PCR.

### Annotation of contig 1 and contig 2

**Figure 2:**
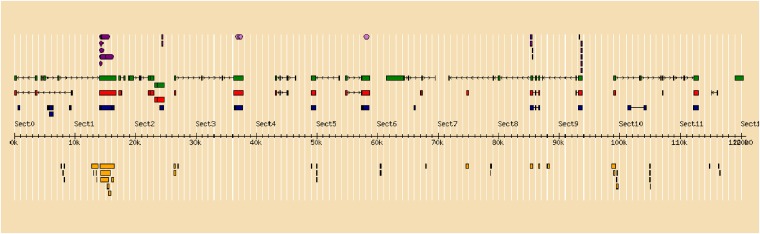
Annotation of contig 1. The sequence of Contig 1 has been submitted to GenBank (KP898411). The sequence was annotated using the Genomics Research on Atlantic Salmon Project (GRASP) annotation pipeline (see http://trutta.mbb.sfu.ca/bacannotations/GRASPbac.html and select Information for a schematic of the work flow). The full annotation data can be seen by going to http://trutta.mbb.sfu.ca/bacannotations/GRASPbac.html and choosing SOSDY_contig291 from the Atlantic salmon BAC list. Purple indicates conserved domain; green indicates a predicted protein by GeneScan from unmasked sequence using BLASTp against the UniRef database; red indicates a predicted protein by GeneScan from masked sequence using BLASTp against the UniRef database; blue shows the results of BLASTx comparisons against the nonredundant NCBI protein database; and yellow shows Atlantic salmon ESTs identified in the sequence using BLAT.

**Figure 3:**
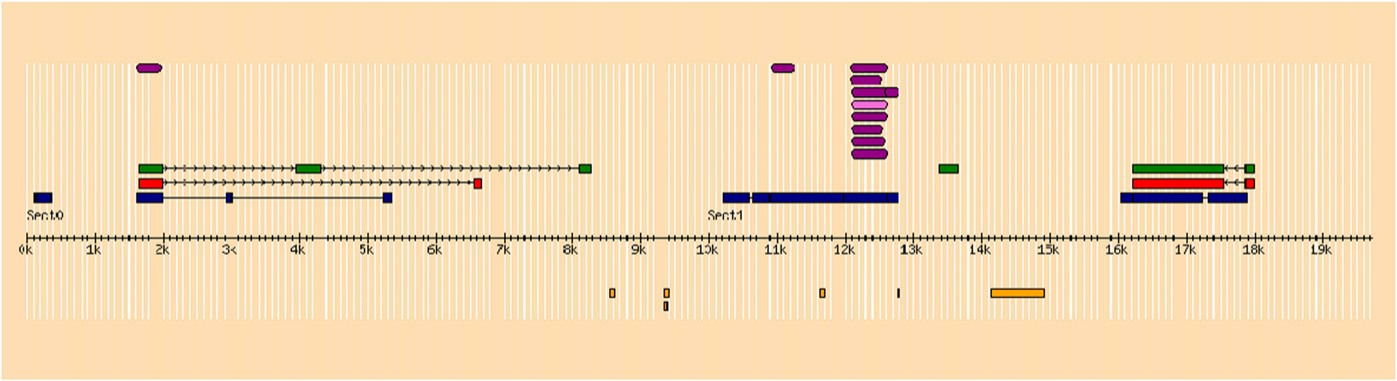
Annotation of contig 2. The sequence of Contig 2 has been submitted to GenBank (KP898412). The sequence was annotated using the Genomics Research on Atlantic Salmon Project (GRASP) annotation pipeline (see http://trutta.mbb.sfu.ca/bacannotations/GRASPbac.html and select Information for a schematic of the work flow). The full annotation data can be seen by going to http://trutta.mbb.sfu.ca/bacannotations/GRASPbac.html and choosing SOSDY_contig292 from the Atlantic salmon BAC list. Purple indicates conserved domain; green indicates a predicted protein by GeneScan from unmasked sequence using BLASTp against the UniRef database; red indicates a predicted protein by GeneScan from masked sequence using BLASTp against the UniRef database; blue shows the results of BLASTx comparisons against the nonredundant NCBI protein database; and yellow shows Atlantic salmon ESTs identified in the sequence using BLAT.

The annotation of contig 1 is shown in Figure 2. There are several predicted Zinc Finger proteins, a few tripartite motif-containing proteins, as well as some uncharacterized putative proteins. However, applying the annotation pipeline to contig 2 identified exons 2, 3, and 4 of the sdY gene and the interferon 3 (IFR-3) domain in exon 2 (Figure 3). Exon 1 of the sdY gene is only 28 bp in length, which is below the threshold of our annotation pipeline for predicting an exon.

When we annotated the individual BAC sequences corresponding to contig 2, we found the four exons of sdY in S0524M13; however, although S0227M24 and S0367C01 contained exons 2, 3, and 4 of sdY, neither appeared to contain exon 1. We wondered if the genomic region between contig 1 and contig 2 was unstable and prone to rearrangements including deletions. When we streaked out an *E. coli* colony containing S0524M13 that appeared to contain all four exons of the sdY gene and tested individual BAC clone colonies from this progenitor by PCR using combinations of primers from exons 1, 2, 3, and 4 (Table S1 and Figure S1), we found that often exon 1 and occasionally exon 2 were missing. We conclude that the genomic sequence between contig 1 and contig 2 is unstable, at least when grown in *E. coli*. This may help to explain the observation of Eisbrenner *et al.* (2014) that a few Tasmanian Atlantic salmon females contain part of the sdY gene.

We designed pairs of primers for PCR analyses along the length of contig 1 and contig 2 (Table S1) and tested them for amplification products from DNA isolated from male and female Atlantic salmon. As illustrated in Figure 1, all primer pairs designed from contig 1 amplified DNA from both sexes. However, primer pairs in the first 8.5 kb of contig 2 only amplified DNA from male Atlantic salmon, whereas primer pairs from 14.7 kb onwards amplified both male and female Atlantic salmon DNA (Figure 1). The sdY gene is located within the first 6 kb of contig 2. Therefore, we predict that there is a breakpoint somewhere between 3 kb and 9.1 kb from the 3′ end of the sdY gene that defines one end of what may be a genomic cassette that contains sdY, the salmonid sex-determining gene. The other end of the cassette is presumably in the unstable genomic region between contig 1 and contig 2.

**Figure 4:**
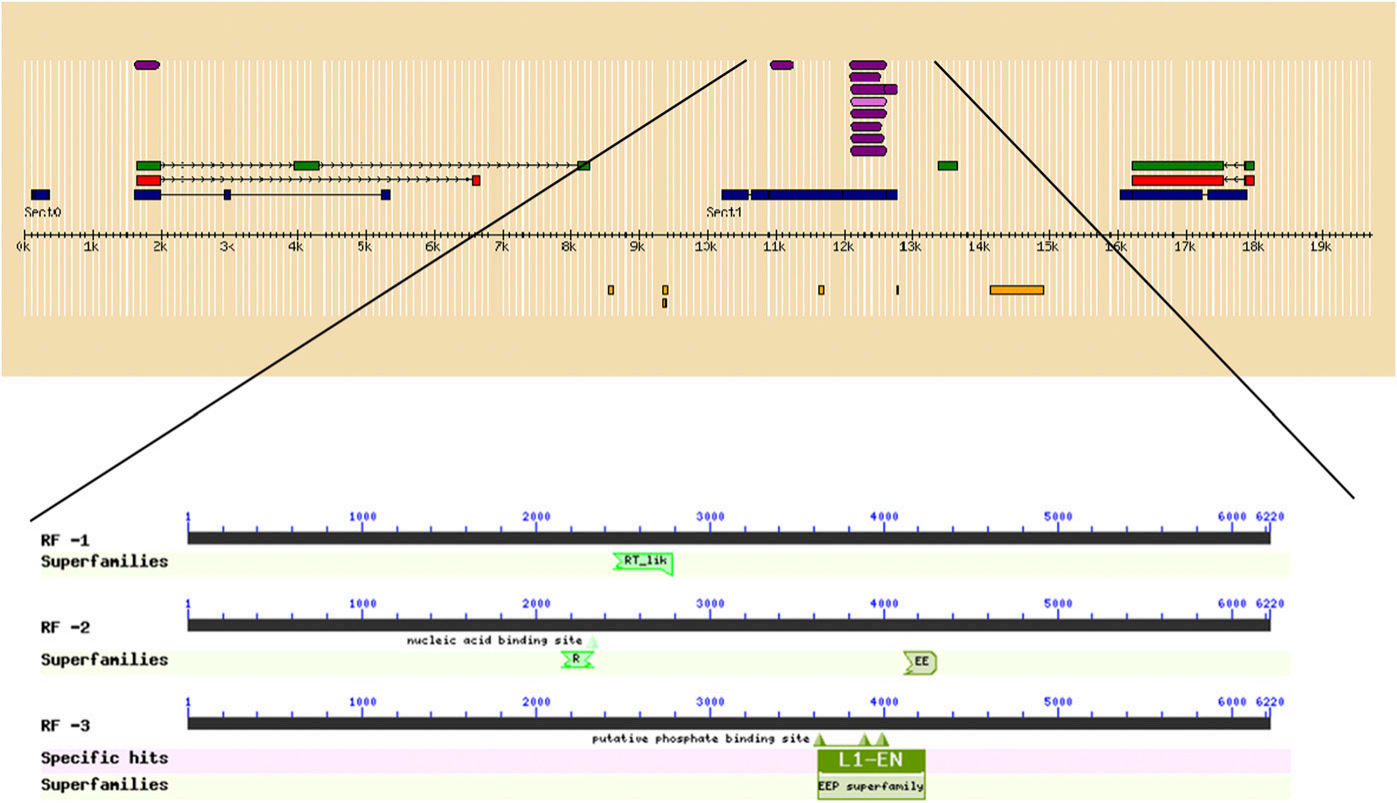
Conserved domains in genomic region of Contig 2 between male-specific DNA amplification and amplification of DNA from both male and female Atlantic salmon. See legends for Figure 2 and Figure 3 for explanation of colors and annotation pipeline.

The annotation pipeline predicted the presence of a pol-like encoding gene in contig 2 between 11 kb and 14.5 kb. This is in the region between DNA sequences found only in male Atlantic salmon and those common to males and females. To explore the nature of the 6220 bp between the end of the primers that amplified only male Atlantic salmon DNA and the primers that amplified DNA from both male and female Atlantic salmon (Figure 1), we compared it with NCBI’s conserved domain database (Marchler-Bauer *et al.* 2015). As expected from the annotation pipeline results shown in Figure 3, the domains that were predicted included members of the RT_like (reverse transcriptase–like) superfamily, EEP (exonuclease-endonuclease-phosphatase) superfamily, and L1-EN (endonuclease of the non-LTR retrotransposon LINE-1) superfamily (Figure 4). These domains are associated with mobile elements, and it is tempting to speculate that this indicates that this is a highly unstable segment of DNA that constitutes the 3′ end of a cassette that contains the sdY gene and allows it to move around the genome.

### Mapping the sdY gene region in European Atlantic salmon

We found a microsatellite marker (Figure 1, Table S1) that was informative in both parents of the SALMAP Br5 mapping family (Danzmann *et al.* 2008). Using the LINKMFEX package (Danzmann and Gharbi 2001) and the genotyping dataset from Danzmann *et al.* (2008), we mapped this marker to linkage group 1 (data not shown), which was where the phenotypic sex locus (SEX) was previously mapped in European Atlantic salmon (Woram *et al.* 2003; Danzmann *et al.* 2008). Linkage group 1 corresponds to Atlantic salmon chromosome 2 (Ssa02) (Artieri *et al.* 2006; Phillips *et al.* 2009). Using microsatellite markers, Eisbrenner *et al.* (2014) mapped SEX to Ssa02, Ssa03, and Ssa06 in Tasmanian Atlantic salmon male lineages. The Tasmanian Atlantic salmon originated from the River Phillip in Nova Scotia on Canada’s east coast. We have previously shown that Atlantic salmon from Europe and North America differ in their karyotypes and chromosomal organization (Lubieniecki *et al.* 2010; Brenna-Hansen *et al.* 2012). Therefore, we wondered if this would also be reflected in the chromosomal location of sdY, especially in Tasmanian male lineages, in which SEX maps to Ssa02, Ssa03, or Ssa06 (Eisbrenner *et al.* 2014).

### FISH analyses with BACs containing sdY

**Figure 5:**
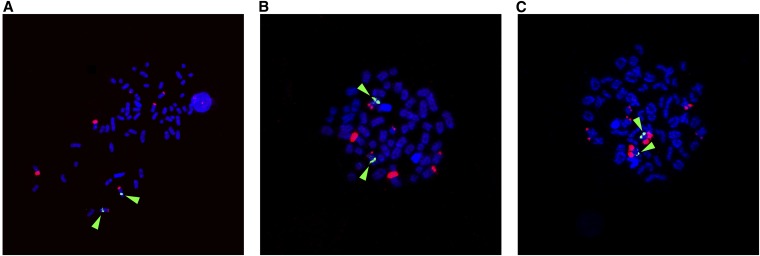
*sdY* chromosomal locations of European Atlantic salmon revealed by FISH using BAC probe, S0524M13, containing *sdY* sequences as part of insert. S0524M13 red fluorescence. Green arrows show S0033O17 (Ssa03 marker), S0332O19 (Ssa06 marker) and S0227A12 (Ssa02 marker) in A, B and C, respectively.

We were curious to determine if S0524M13, the BAC that contains the full sequence of the sdY gene, would hybridize to chromosome 2 (Ssa02) in European Atlantic salmon, as would be predicted from the results of Artieri *et al.* (2006). Therefore, we performed dual FISH analyses with S0524M13 in combination with other BACs from the set that was used to define the Atlantic salmon chromosomes and integrate them with the linkage groups in the genetic map (Phillips *et al.* 2009). To our surprise, S0524M13 hybridized to three pairs of European Atlantic salmon chromosomes, which were identified as Ssa02, Ssa03, and Ssa06 (Figure 5). These are the same chromosomes that Eisbrenner *et al.* (2014) mapped SEX to in Tasmanian Atlantic salmon male lineages. These results suggest that it was not by chance that SEX mapped to these particular chromosomes; rather, there are similar genomic regions in these chromosomes that may facilitate the jumping of the sex-determining gene around the genome. We emphasize that any given male Atlantic salmon is likely to have a single sdY gene, but the BAC containing the sdY gene contains flanking regions that must occur in both male and female salmon and have paralogs that are located in more than one chromosome.

**Figure 6:**
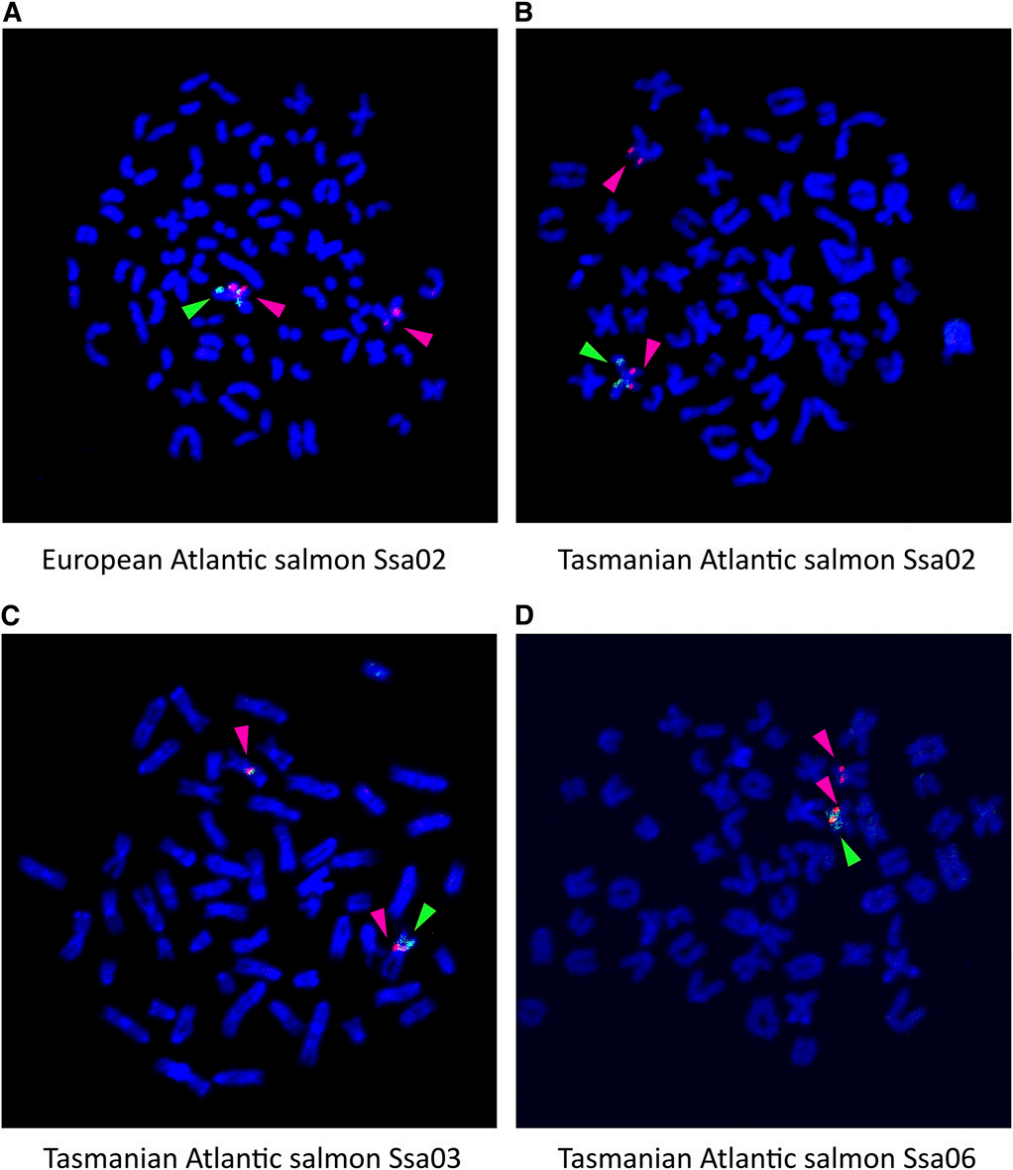
*sdY* chromosomal locations in male European (A) and Tasmanian (B, C, and D) Atlantic salmon revealed by FISH using a plasmid probe containing the sdY gene sequence. Green arrow: *sdY*-containing plasmid; red arrow: S0227A12 (Ssa02 marker), S0227A12 (Ssa02 marker), S0033O17 (Ssa03 marker), and S0332O19 (Ssa06 marker) in (A), (B), (C), and (D), respectively.

To determine if the sdY gene is located on these chromosomes in the Tasmanian male lineages, we cloned the sdY gene from Atlantic salmon into a plasmid vector using PCR amplification with primers from the 5′ and 3′ ends of the gene (Table S1). This resulted in an insert of 5118 bp that did not contain any putative repetitive mobile elements on either side of the sdY gene, but the possibility that these are located within intronic regions could not be excluded . When this sdY-containing plasmid was used as a probe in dual FISH analyses with BACs known to hybridize to Ssa02, Ssa03, or Ssa06, the results revealed that the sdY gene was indeed on the chromosomes to which SEX had been mapped in these male lineages (Figure 6). Moreover, the *sdY*-containing plasmid probe hybridized only to one of the chromosomes, presumably the Y chromosome. This result verified that sdY had indeed jumped around the genome and was located in the different sex chromosomes. It is worth noting that the FISH analyses showed that the *sdY*-containing plasmid probe hybridized to the tips of the short arm in Ssa03 and Ssa06 and to the end of long arm in Ssa02.

### Comparisons of sdY sequences in Atlantic salmon populations and other species

We wanted to determine if the sdY gene sequences were highly conserved in Tasmanian male lineages, which had SEX mapped to Ssa02, Ssa03, or Ssa06 (Eisbrenner *et al.* 2014). Our rationale for doing so was that if these sequences were identical, then it would suggest a recent jumping of the sdY gene around the genome. In contrast, if there were several changes, then it would suggest that some time had elapsed since the sdY gene jumped chromosome. Using primers (Table S1) we amplified and sequenced the sdY genes from brown trout and three Tasmanian Atlantic salmon males, which had SEX mapped to Ssa02, Ssa03, or Ssa06 (Eisbrenner *et al.* 2014). These sequences were compared with the European Atlantic salmon sdY gene sequence from contig 2 as well as sdY gene sequences from rainbow trout and Chinook salmon (Oncorhynchus tshawytscha) (Figure S1). The sdY gene consists of four exons of similar length (exon 1, 28 bp; exon 2, 360–363 bp; exon 3, 101 bp; and exon 4, 90 bp), with the exception of Chinook salmon and rainbow trout in which exon 2 is shorter by three nucleotides. The length of the gene (from start to stop codon) varies depending on the species and ranges from 3693 bp in brown trout to 6132 bp in Chinook salmon, whereas in the rainbow trout it is 5005 bp.

The sdY genes in Atlantic salmon from different populations have similar sizes (European Atlantic salmon Ssa02, 4190 bp; Tasmanian Ssa06, 4281 bp; Tasmanian Ssa02, 4292 bp; Tasmanian Ssa03, 4294 bp). The differences in length of the Atlantic salmon sdY genes come from different numbers of a CT dinucleotide repeat in Intron I. Because changes in microsatellite length are unlikely to reflect accurately the evolutionary relationship of the sdY genes in different Atlantic salmon male lineages, it was not possible to discern a pattern to the jumping of the sdY genes in Tasmanian Atlantic salmon. Therefore, we are unable to predict whether the sdY gene jumping arose in the ancestral population in the River Philip in Nova Scotia or occurred after the introduction of Atlantic salmon to Australia in the 1960s.

The major reason for the difference in sdY length between Salmo and Oncorhynchus species is an insertion of 2232 bp in Chinook salmon and 1113 bp in rainbow trout in intron 1 of the gene, relative to Salmo sp. Almost the entire length of these insertions are known salmonid repeats. In the case of Chinook salmon, 93.1% of the insertion is masked by salmon v2.0 repeat masker http://lucy.ceh.uvic.ca/repeatmasker/cbr_repeatmasker.py (2077 out of 2232 base pairs); however, in rainbow trout 91.8% (1022 out of 1113 bp) is identified as known salmonid repeats.

At the protein level the sdY sequences are nearly identical in terms of length (Figure S2). The sdY protein of all Atlantic salmon and brown trout is 193 amino acids in length, whereas Chinook salmon and rainbow trout sdYs contain 192 amino acids. There is 100% sequence identity among the sdYs from Tasmanian Atlantic salmon, in which the sdY genes are located on three different chromosomes, and European Atlantic salmon. There are 170 invariant amino acid sites in the sdYs from Salmo and Oncorhynchus species.

### Comparison of the sdY containing genomic sequences of Atlantic salmon, rainbow trout, and Chinook salmon

When Brunelli *et al.* (2008) characterized Y-specific regions of the genomes of rainbow trout (EU081756) and Chinook salmon (DQ393568.1), they found that there was a discontinuity on the 5′ side of the Omy Y1 male-specific marker in a sequence annotated as “similar to ReO-6.” This region of the rainbow trout genome was expanded and characterized in more detail (Phillips *et al.* 2013; Faber-Hammond *et al.* 2015). We compared the sequence of Atlantic salmon contig 1 with these rainbow trout and Chinook salmon sequences, but found no sequence similarity of any significance. The only sequences in common between Atlantic salmon and the Onchorhynchus sp. were identified as known repetitive elements by the Atlantic salmon repeat database (http://lucy.ceh.uvic.ca/repeatmasker/cbr_repeatmasker.py; de Boer *et al.* 2007). We then compared Atlantic salmon contig 2, which contains the sdY gene with the rainbow trout and Chinook salmon sequences. Apart from the sdY gene, there were no surrounding regions in common. When we examined the immediate 5′ sequences of the sdY gene in these three species, only approximately 222 bp upstream from the ATG start codon shared sequence similarity between Atlantic salmon and the Onchorhynchus sp. (Figure S3), although the sequence similarity extended when only rainbow trout and Chinook salmon were compared, reflecting their shared evolutionary ancestry. These results add considerable weight to the jumping gene hypothesis for the salmonid sex-determining gene. In addition, it appears that the sdY gene is in a cassette flanked by repetitive, transposable-like elements, and these may facilitate the transposition or translocation of sdY within a species. The result of within-species sdY genomic movements is consistent with what has been observed not only for Atlantic salmon in Tasmania (Eisbrenner *et al.* 2014) but also in populations of Arctic charr (Salvelinus alpinus) (Moghadam *et al.* 2007; Kuttner *et al.* 2011). The movement of the sdY gene within different species would lead to adjacent genetic markers becoming different between species, as was observed by Woram *et al.* (2003).

## Discussion

Teleosts display the full gamut of sex determination mechanisms from hermaphroditism to strictly gonochoristic with either male or female heterogamety to environmental sex determination (Devlin and Nagahama 2002; Mank and Avise 2009; Bachtrog *et al.* 2014). The work of Phillips *et al.* (2001) revealed that members of the Salmoninae (salmon, trout, and charr) did not share the same sex chromosomes as lake trout (*Salvelinus namaycush*), and these species appeared to have different sex chromosomes from one another. The genetic mapping of male phenotype (*SEX*) in Atlantic salmon, brown trout, rainbow trout, and Arctic charr (Woram *et al.* 2003) supported the cytogenetic analyses. Together these results led to two main hypotheses regarding the sex-determining gene in salmonids: (1) there are several sex-determining genes in this family or (2) there is a common sex-determining gene that has the capability to move around the genome either by transposition or by translocation (Phillips *et al.* 2001; Woram *et al.* 2003; Davidson *et al.* 2009). The discovery of *sdY*, the sex-determining gene in rainbow trout (Yano *et al.* 2012), and its presence in many male salmonids (Yano *et al.* 2013) suggested that the sex-determining jumping gene hypothesis was correct, but it did not provide a mechanism for this phenomenon. Our results from mapping *SEX* in Tasmanian Atlantic salmon male lineages (Eisbrenner *et al.* 2014) also lent support to the sex-determining jumping gene hypothesis as we found three chromosomes on which *SEX* could occur. However, although *SEX* mapped to Ssa02, Ssa03, or Ssa06, and although we found the presence of *sdY* in most males in these families, we could not be sure that *sdY* was located on these chromosomes. Therefore, we set out to characterize the sdY gene region in Atlantic salmon and to examine its chromosomal organization in different Tasmanian Atlantic salmon male lineages.

We isolated three BACs containing the sdY gene from a library constructed from DNA from a European Atlantic salmon male (Ng *et al.* 2005; Thorsen *et al.* 2005) and determined the genomic sequence around *sdY*. The region upstream from the start of the sdY gene in Atlantic salmon appeared to be unstable, and this region corresponds to the discontinuity seen in the genomic sequences of rainbow trout and Chinook salmon (Brunelli *et al.* 2008). There was little sequence similarity between Atlantic salmon and rainbow trout 3′ downstream from the end of the sdY gene. This region corresponded to the boundary between DNA that was only found in male Atlantic salmon and genomic sequences that occur in both male and female Atlantic salmon. A closer examination of this genomic sequences indicated that it was home to genes that contain signatures of repetitive elements such as transposons (Figure 4). These results define the boundaries of what appears to be an *sdY* cassette that fits the sex-determining jumping gene model.

Cytogenetic dual FISH analyses showed that a BAC containing the sdY gene hybridized not only to its expected location on European Atlantic salmon chromosomes (*i.e.*, Ssa02) (Artieri *et al.* 2006) but also to Ssa03 and Ssa06 (Figure 5), where *SEX* had been mapped in Tasmanian families (Eisbrenner *et al.* 2014). These results suggested that there are similar sequences in these regions of the genome and that this may facilitate the movement of the sdY gene cassette. The use of a plasmid probe that contains the sdY gene without flanking genomic sequences in dual FISH analyses showed that the sdY gene did indeed localize to the chromosomes where *SEX* had been mapped in different Tasmanian Atlantic salmon families. Moreover, the probe hybridized primarily to one of the sex chromosomes (Figure 6), as would be expected of a male-specific gene. The hybridization of the plasmid sdY gene probe to the telomeric ends of the short arms of Ssa03 and Ssa06 and, as had been predicted previously (Artieri *et al.* 2006), to the end of the long arm of Ssa02, supports the hypothesis of a male-specific gene (*sdY*) having been transposed to the ends of different chromosomes not only in different species but also within a species (Phillips *et al.* 2001; Woram *et al.* 2003).

We searched the literature for other examples of the transposition of a sex-determining gene within a species. Although there is considerable evidence for turnover of sex chromosomes in closely related species of fish (Tanaka *et al.* 2007; Ross *et al.* 2009; Kitano and Peichel 2012; Bachtrog *et al.* 2014), in many instances the actual sex-determining gene(s) in the different species is/are not known. These observations rely on genetic mapping of *SEX* and comparing the locations of sex-linked markers and autosomal markers in different species. On occasion, these observations are coupled with chromosomal analyses with hybridization probes (Ross *et al.* 2009). In closely related species in the genus *Oryzias* it was shown that the sex-determining gene, *DMY*, in *O. latipes* (Medaka) is not the sex-determining gene in *O. luzonensis* (Tanaka *et al.* 2007); subsequently, the sex-determining gene in this species was found to be *Gsdf^Y^* (Myosho *et al.* 2012). It has been suggested that multiple mechanisms contribute to sex chromosome turnover, including a new master sex-determining gene arising on an autosome, the fusion of an existing Y chromosome with an autosome, and by transposition of an existing sex-determining gene to an autosome (Kitano and Peichel 2012). Our results clearly show that the transposition of a sex-determining gene, *sdY*, has occurred in Atlantic salmon. We do note, however, that this phenomenon of a jumping sex-determining factor has been documented in the fly *Megaselia scalaris* (Traut and Willhoeft 1990).

### Conclusions

We have characterized the sdY genomic environment in Atlantic salmon. In doing so, we identified a cassette containing the sdY gene that is flanked by repetitive, transposable-like elements. These results suggest a mechanism whereby the salmonid sex-determining locus can move around the genome, giving the impression that there are multiple sex-determining genes both within and between salmonid species.

## 

## Supplementary Material

Supporting Information

## References

[bib1] Artieri C. G. Mitchell L. A. Ng S. H. S. Parisotto S. E. Danzmann R. G., 2006 Identification of the sex-determining locus of Atlantic salmon *Salmo salar* on chromosome 2. Cytogenet. Genome Res. 112: 152–159. 16276105 10.1159/000087528

[bib2] Bachtrog D. Mank J. E. Peichel C. L. Kirkpatrick M. Otto S. P., 2014 Sex determination: Why so many ways of doing it? PLoS Biol. 12: e1001899. 24983465 10.1371/journal.pbio.1001899 PMC4077654

[bib3] Brenna-Hansen S. Li J. Kent M. P. Boulding E. G. Dominik S., 2012 Chromosomal differences between European and North American Atlantic salmon discovered by linkage mapping and supported by fluorescence in situ hybridization analysis. BMC Genomics 13: 432. 22928605 10.1186/1471-2164-13-432 PMC3495403

[bib4] Brunelli J. P., K. J. Wertzler, K. Sundin, and G. H. Thorgaard, 2008 Y-specific sequences and polymorphisms in rainbow trout and Chinook salmon. Genome 51: 739–748. 18772952 10.1139/G08-060

[bib5] Cavileer T. D. Hunter S. S. Olsen J. Wenburg J. Nagler J. J., 2015 A sex-determining gene (sdY) assay shows discordance between phenotypic and genotypic sex in wild populations of Chinook salmon. Trans. Am. Fish. Soc. 144: 423–430.

[bib6] Danzmann R. G. Gharbi K., 2001 Gene mapping in fishes: a means to an end. Genetica 111: 3–23. 11841175 10.1023/a:1013713431255

[bib7] Danzmann R. G. Davidson E. A. Ferguson M. M. Gharbi K. Koop B. F., 2008 Distribution of ancestral proto-Actinopterygian chromosome arms within the genomes of 4R-derivative salmonid fishes (Rainbow trout and Atlantic salmon). BMC Genomics 9: 557. 19032764 10.1186/1471-2164-9-557 PMC2632648

[bib8] Davidson W. S. Huang T. Fujiki K. von Schalburg K. R. Koop B. F., 2009 The sex determining loci and sex chromosomes in the family Salmonidae. Sex Dev. 3: 78–87. 19684453 10.1159/000223073

[bib9] Davidson W. S. Koop B. F. Jones S. J. M. Iturra P. Vidal R., 2010 Sequencing the genome of the Atlantic salmon *Salmo salar*. Genome Biol. 11: 403. 20887641 10.1186/gb-2010-11-9-403 PMC2965382

[bib10] de Boer J. G. Yazawa R. Davidson W. S. Koop B. F., 2007 Bursts and horizontal evolution of DNA transposons in the speciation of pseudotetraploid salmonids. BMC Genomics 8: 422. 18021408 10.1186/1471-2164-8-422 PMC2198921

[bib11] Devlin R. H. Nagahama Y., 2002 Sex determination and sex differentiation in fish: an overview of genetic, physiological, and environmental influences. Aquaculture 208: 191–364.

[bib12] Eisbrenner W. D. Botwright N. Cook M. Davidson E. A. Dominik S., 2014 Evidence for multiple sex-determining loci in Tasmanian Atlantic salmon Salmo salar. Heredity 113: 86–92. 23759729 10.1038/hdy.2013.55 PMC4815647

[bib13] Ewing B. Green P., 1998 Base-calling of automated sequencer traces using phred. II. Error probabilities. Genome Res. 8: 186–194. 9521922

[bib14] Ewing B. Hillier L. Wendl M. C. Green P., 1998 Base-calling of automated sequencer traces using phred. I. Accuracy assessment. Genome Res. 8: 175–185. 9521921 10.1101/gr.8.3.175

[bib15] Faber-Hammond J. J. Phillips R. B. Brown K. H., 2015 Comparative analysis of the shared sex-determination region (SDR) among salmonid fishes. Genome Biol. Evol. . 10.1093/gbe/evv123. PMC4524489 26112966

[bib16] Gordon D. Abajian C. Green P., 1998 Consed: agraphical tool for sequence finishing. Genome Res. 8: 195–202. 9521923 10.1101/gr.8.3.195

[bib17] Johnston I. A. Strugnell G. McCracken M. L. Johnstone R., 1999 Muscle growth and development in normal-sex-ratio and all-female diploid and triploid Atlantic salmon. J. Exp. Biol. 202: 1991–2016. 10393816 10.1242/jeb.202.15.1991

[bib18] Johnstone R. MacLachlan P. M., 1994 Further observations on the sex inversion of Atlantic salmon, *Salmo salar*, using 17α methyl testosterone. Aquacult. Fish. Manage. 25: 855–859.

[bib19] Johnstone K. A. Ciborowski K. L. Lubieniecki K. P. Chow W. Phillips R. B., 2009 Genomic organization and evolution of the vomeronasal type 2 receptor-like (OlfC) gene clusters in Atlantic salmon, *Salmo salar*. Mol. Biol. Evol. 26: 1117–1125. 19221009 10.1093/molbev/msp027 PMC2668830

[bib20] King, H., N. Ruff, B. Evans, and N. Elliott, 2012 Evidence that sex differentiation in Atlantic salmon is thermolabile. ICISB, Oslo P-018.

[bib21] Kitano J. Peichel C. L., 2012 Turnover of sex chromosomes and speciation in fishes. Environ. Biol. Fishes 94: 549–558. 26069393 10.1007/s10641-011-9853-8 PMC4459657

[bib22] Kuttner E. Nilsson J. Skulason S. Gunnarsson S. Ferguson M. M., 2011 Sex chromosome polymorphisms in Arctic charr and their evolutionary origins. Genome 54: 852–861. 21970434 10.1139/g11-041

[bib23] Li J. Phillips R. B. Harwood A. S. Koop B. F. Davidson W. S., 2011 Identification of the sex chromosomes of brown trout *Salmo trutta* and their comparison with the corresponding chromosomes in Atlantic salmon *Salmo salar* and rainbow trout *Oncorhynchus mykiss*. Cytogenet. Genome Res. 133: 25–33. 21252487 10.1159/000323410

[bib24] Lubieniecki K. P. Jones S. L. Davidson E. A. Park J. Koop B. F., 2010 Comparative genomic analysis of Atlantic salmon, Salmo salar, from Europe and North America. BMC Genet. 11: 105. 21092310 10.1186/1471-2156-11-105 PMC2995484

[bib44] Mank J. E. Avise J. C., 2009 Evolutionary diversity and turn-over of sex determination in teleost fished. Sex Dev. 3: 60–67. 19684451 10.1159/000223071

[bib25] Marchler-Bauer A. Derbyshire M. K. Gonzales N. R. Lu S. Chitsaz F., 2015 CDD: NCBI’s conserved domain database. Nucleic Acids Res. 43: D222–D226. 25414356 10.1093/nar/gku1221 PMC4383992

[bib26] Moghadam H. K. Ferguson M. M. Danzmann R. G., 2007 Linkage variation at the sex determining locus within Fraser strain Arctic charr *Salvelinus alpinus*. J. Fish Biol. 71: 294–301.

[bib45] Myosho T. Otake H. Masuyama H. Matsuda M. Kuroki Y., 2012 Tracing the emergence of a novel sex-determining gene in Medaka *Oryzias luzonensis*. Genetics 191: 163–170. 22367037 10.1534/genetics.111.137497 PMC3338257

[bib27] Ng S. H. S. Artieri C. G. Bosdet I. E. Chiu R. Danzmann R. G., 2005 A physical map of the genome of Atlantic salmon, Salmo salar. Genomics 86: 396–404. 16026963 10.1016/j.ygeno.2005.06.001

[bib28] Palibroda, E. I., K. Lubieniecki, Y. Y. Y. Lai, and W. S. Davidson, 2013 *The Organization of the Atlantic Salmon (Salmo salar) sdY Gene and Its Potential Genomic Environments*. PAG XXIV, San Diego, CA.

[bib29] Phillips R. B. Konkol N. R., K. M. Reed, and J. D. Stein, 2001 Chromosome painting supports lack of homology among sex chromosomes in *Oncorhynchus*, *Salmo* and *Salvelinus* (Salmonidae). Genetica 111: 119–123. 11841160 10.1023/a:1013743431738

[bib30] Phillips R. B. Keatley K. A. Morasch M. R. Ventura A. B. Lubieniecki K. P., 2009 Assignment of Atlantic salmon (*Salmo salar*) linkage groups to specific chromosomes: conservation of large syntenic blocks corresponding to whole chromosome arms in rainbow trout (*Oncorhynchus mykiss*). BMC Genet. 10: 46. 19689812 10.1186/1471-2156-10-46 PMC2734554

[bib31] Phillips R. B. DeKoning J. J. Brunelli J. P. Faber-Hammond J. J. Hansen J. D., 2013 Characterization of the OmyY1 region on the rainbow trout chromosome. Int. J. Genomics 2013: 261730. 23671840 10.1155/2013/261730 PMC3647546

[bib32] Quillet E. Aubard G. Queau L., 2002 Mutation in a sex-determining gene in rainbow trout: detection and genetic analysis. J. Hered. 9: 91–99. 12140268 10.1093/jhered/93.2.91

[bib33] Quinn N. L. Boroevich K. A. Lubieniecki K. P. Chow W. Davidson E. A., 2010 Genomic organization and evolution of the Atlantic salmon hemoglobin repertoire. BMC Genomics 11: 539. 20923558 10.1186/1471-2164-11-539 PMC3091688

[bib34] Ross J. A. Urton J. R. Boland J. Shapiro M. D. Peichel C. L., 2009 Turnover of sex chromosomes in the stickleback fishes (Gasterosteidae). PLoS Genet. 5: e1000391. 19229325 10.1371/journal.pgen.1000391 PMC2638011

[bib35] Tanaka K. Takehana Y. Naruse K. Hamaguchi S. Sakaizumi M., 2007 Evidence for different origins of sex chromosomes in closely related *Oryzias* fishes: substitution of the master sex-determining gene. Genetics 177: 2075–2081. 17947439 10.1534/genetics.107.075598 PMC2219477

[bib36] Thorgaard G. H., 1977 Heteromorphic sex chromosomes in male rainbow trout. Science 196: 900–902. 860122 10.1126/science.860122

[bib37] Thorgaard G. H., 1978 Sex chromosomes in the sockeye salmon: a Y-autosome fusion. Can. J. Genet. Cytol. 20: 349–354. 570442 10.1139/g78-039

[bib38] Thorsen J. Zhu B. Frengen E. Osoegawa K. de Jong P. J., 2005 A highly redundant BAC library of Atlantic salmon (Salmo salar): an important tool for salmon projects. BMC Genomics 6: 50. 15807896 10.1186/1471-2164-6-50 PMC1082906

[bib39] Traut W. Willhoeft U., 1990 A jumping sex determining factor in the fly *Megaelia scalaris*. Chromosoma 99: 407–412.

[bib40] Valdivia K. Jouanno E. Volff J.-N. Galiana-Arnoux D. Guyomard R., 2014 High temperature increases the masculinization rate of the all-female (XX) rainbow trout “Mal” population. PLoS One . 10.1371/journal.pone.011335 PMC4264747 25501353

[bib41] Woram R. A. Gharbi K. Sakamoto T. Hoyheim B. Holm L. E., 2003 Comparative genome analysis of the primary sex-determining locus in salmonid fishes. Genome Res. 13: 272–280. 12566405 10.1101/gr.578503 PMC420375

[bib42] Yano A. Guyomard R. Nicol B. Jouanno E. Quillet E., 2012 An immune-related gene evolved into the master sex-determining gene in rainbow trout, *Oncorhynchus mykiss*. Curr. Biol. 22: 1423–1428. 22727696 10.1016/j.cub.2012.05.045

[bib43] Yano A. Nicol B. Jouanno E., E. Quillet, A. Fostier, *et al*, 2013 The sexually dimorphic on the Y-chromosome gene (*sdY*) is a conserved male-specific Y-chromosome sequence in many salmonids. Evol. Appl. 6: 486–496. 23745140 10.1111/eva.12032 PMC3673476

