# Supplementary material for: Genomic Instability of the Sex-Determining Locus in Atlantic Salmon (Salmo salar)
Source: G3 (Bethesda). 2015 Sep 22;5(11):2513–22. doi: 10.1534/g3.115.020115 (PMC4632069; doi:10.1534/g3.115.020115)
Supplement: Supporting Information [file supp_5_11_2513__index.html]

Genomic Instability of the Sex-Determining Locus in Atlantic Salmon (Salmo salar) — Supporting Information 

# Genomic Instability of the Sex-Determining Locus in Atlantic Salmon (*Salmo salar*)

## Supporting Information for Lubieniecki *et al.*, 2015

**Files in this Data Supplement:**

- Supporting Information - Figures S1-S3 and Table S1 (PDF, 146 KB)
- Figure S1 - CLUSTAL 2.1 multiple sequence alignment of sdY from brown trout (BT), three Tasmanian and one European Atlantic salmon, Chinook salmon and rainbow trout (RT). The boundaries of exons and locations of primers are shown. (PDF, 63 KB)
- Figure S2 - CLUSTAL 2.1 multiple sequence alignment of sdY from brown trout (BT), three Tasmanian and one European Atlantic salmon, Chinook Ssalmon and rainbow trout (RT). (PDF, 24 KB)
- Figure S3 - CLUSTAL multiple sequence alignment by Kalign (2.0) of 5' sdY sequences of Atlantic salmon, rainbow trout and Chinook salmon. (PDF, 26 KB)
- Table S1 - Nucleotide sequences of primers and probes. (PDF, 93 KB)
