# Supplementary material for: Genomic Instability of the Sex-Determining Locus in Atlantic Salmon (Salmo salar)
Source: G3 (Bethesda). 2015 Sep 22;5(11):2513–22. doi: 10.1534/g3.115.020115 (PMC4632069; doi:10.1534/g3.115.020115)
Supplement: Supporting Information [file supp_g3.115.020115_020115SI.pdf]

Genomic instability of the sex-determining locus in Atlantic salmon (*Salmo salar*)

Krzysztof P. Lubieniecki,[*] Song Lin,[*] Emily I. Cabana,* Jieying Li,* Yvonne Y.Y. Lai* and William S. Davidson[*,1]

[*]Department of Molecular Biology and Biochemistry, Simon Fraser University, Burnaby, BC, V5A 1S6 Canada

[1]Corresponding author: Department of Molecular Biology and Biochemistry, Simon Fraser University, Burnaby, BC, V5A 1S6 Canada. E-mail: **wdavidso@sfu.ca**

```
BT_sdY                      ----------CTCCCTT--ACTAATCATTTTACATGAAAAAATGCATATC
AS_Tasmanian_Ssa02_sdY      ----------CTCCCCTTAACTAATCATTTTACATGAAAAAATGCATATC
AS_Tasmanian_Ssa03_sdY      ---------ACTCCCCTT-ACTAATCATTTTACATGAAAAAATGCATATC
AS_European_sdY             GAGAGAAACACTCCCCTTAACTAATCATTTTACATGAAAAAATGCATATC
AS_Tasmanian_Ssa06_sdY      ---------ACTCCCCTTAACTAATCATTTTACATGAAGAAATGCATATC
Chinook_sdY                 ----------CTCCCCTTAACTAATACTTTTACATGAAAACATGCACATA
RT_sdY                      ------AACACTCCCCTTAACTAATACTTTTACATGAAAACATGCTCATA
                                      ***** *  ******  *********** * ****  **

BT_sdY                      AAAAACTCCAGCGCATGTTTAAAAGCAGTCCTTAGAGGTTTTGAATCCAC
AS_Tasmanian_Ssa02_sdY      AAAAACTCCAGCGCATGTTTAAAAACAGTCCTTAGAGGTTTTGAATCCAC
AS_Tasmanian_Ssa03_sdY      AAAAACTCCAGCGCATGTTTAAAAACAGTCCTTAGAGGTTTTGAATCCAC
AS_European_sdY             AAAAACTCCAGCGCATGTTTAAAAACAGTCCTTAGAGGTTTTGAATCCAC
AS_Tasmanian_Ssa06_sdY      AAAAACTCCAGCGCATGTTTAAAAACAGTCCTTAGAGGTTTTGAATCCAC
Chinook_sdY                 AAAAACTCCAGCTCATGTTTAAAAGCCGTCCTTAGAATAGTTGAGTCCAT
RT_sdY                      AAAAACTCCAGCTCATGTTTAAAAGCCGTCCTTAGAATATTTGAGTCCAT
                            ************ *********** * *********    **** ****

                                      |      exon1             |
                                      |-------Exon 1F------>       |
BT_sdY                      CTGCCCTTCA|ATGGCTGACAGAGAGGCCAGATTCCAAG|GTACAGAACACA
AS_Tasmanian_Ssa02_sdY      CTGCCCTTCA|ATGGTTGACAGAGAGGCCAGATTCCAAG|GTACAGAACACA
AS_Tasmanian_Ssa03_sdY      CTGCCCTTCA|ATGGTTGACAGAGAGGCCAGATTCCAAG|GTACAGAACACA
AS_European_sdY             CTGCCCTTCA|ATGGTTGACAGAGAGGCCAGATTCCAAG|GTACAGAACACA
AS_Tasmanian_Ssa06_sdY      CTGCCCTTCA|ATGGTTGACAGAGAGGCCAGATTCCAAG|GTACAGAACACA
Chinook_sdY                 CTGCCCTTCA|ATGGCTGACAGAGAGGCCAGAATCCAAG|GTACAGAACACA
RT_sdY                      CTGCCCTTCA|ATGGCTGACAGAGAGGCCAGAATCCAAG|GTACAGAACACA
                            **********|**** **************** ******|************
```

```
BT_sdY                    CAAATATTCAGTATGTTATCTAAGAGTGTTTTGATTTTCATTATTTTGGG
AS_Tasmanian_Ssa02_sdY    CAAATATTCAGTATGTTATCTAAGAGTGTTTTGATTTTCATTATTTTGGG
AS_Tasmanian_Ssa03_sdY    CAAATATTCAGTATGTTATCTAAGAGTGTTTTGATTTTCATTATTTTGGG
AS_European_sdY           CAAATATTCAGTATGTTATCTAAGAGTGTTTTGATTTTCATTATTTTGGG
AS_Tasmanian_Ssa06_sdY    CAAATATTCAGTATGTTATCTAAGAGTGTTTTGATTTTCATTATTTTGGG
Chinook_sdY               CAAACATTCAGTATGTTATGTAAGACTG-TTTGGTTTTCATTATTTTGGG
RT_sdY                    CAAACATTCAGTATGTTATGTAAGACTG-TTTGGTTTTCATTATTTTGGG
                          **** ************** ***** ** **** ****************

BT_sdY                    CCTATGAATTTCTGATGTTAA----------GGACTGTATCTATGTGGTT
AS_Tasmanian_Ssa02_sdY    CCTATGCATTTCTGATGTTGAACAGCTTAA-GGACTGTGTCTATGTGGTT
AS_Tasmanian_Ssa03_sdY    CCTATGCATTTCTGATGTTGAACAGCTTAA-GGACTGTGTCTATGTGGTT
AS_European_sdY           CCTATGCATTTCTGATGTTGAACAG---AA-GGACTGTGTCTATGTGGTT
AS_Tasmanian_Ssa06_sdY    CCTATGCATTTCTGATGTTGAACAGCTTAA-GGACTGTGTCTATGTGGTT
Chinook_sdY               CCTATGAATGTCTGATGTTATACAGCTTACTGTGCTGTGTACATGTGGTT
RT_sdY                    CCTATGAATGTCTGATGTTGTACAGCTTACTGTGCTGTGTATATGTGGTT
                          ****** ** *********            *  **** *  ********

BT_sdY                    ATTTCCATGTCTGATCTTTTGAGGGAAATGCTCAAAATGAGTATATAGCA
AS_Tasmanian_Ssa02_sdY    ATTTCCATGTCTGATCTTTTGAGGGA-------AAAATGAGTATATAGCG
AS_Tasmanian_Ssa03_sdY    ATTTCCATGTCTGATCTTTTGAGGGA-------AAAATGAGTATATAGCG
AS_European_sdY           ATTTCCATGTCTGATCTTTTGAGGGAAATGCTCAAAATGAGTATATAGCG
AS_Tasmanian_Ssa06_sdY    ATTTCCATGTCTGATCTTTTGAGGGAAATGCTCAAAATGAGTATATAGCG
Chinook_sdY               ATTTCCATGTCTGATTTC-------------------------CATGTCT
RT_sdY                    ATTTCCATGTCTGA------------------------------------
                          **************
```

```
BT_sdY                     TTTTGGAATTAG---CTTCAGTTGTACAATTTCATTACTATACGTTTTTT
AS_Tasmanian_Ssa02_sdY     TTTTGGAATTAG---CTTCAGTTGTACAATTTCATTACTATACTTTTTTT
AS_Tasmanian_Ssa03_sdY     TTTTGGAATTAG---CTTCAGTTGTACAATTTCATTACTATACTTTTTTT
AS_European_sdY            TTTTGGAATTAG---CTTCAGTTGTACAATTTCATTACTATACTTTTTTT
AS_Tasmanian_Ssa06_sdY     TTTTGGAATTAG---CTTCAGTTGTACAATTTCATTACTATACTTTTTTT
Chinook_sdY                AATTGGAATGAAACTTTTCAGTTGTACAATTTCATTACTTAACTTTTTTT
RT_sdY                     --TTGGAATGAAACTCTTCAGTTGTACAATTTCATTATTTAACTTTTTTT
                             ******* *     ********************* *  ** ******

BT_sdY                     GTGCTAATTTTATAATGTTCATGTCGCAAATCTGTTATAAAAAGTCAGGA
AS_Tasmanian_Ssa02_sdY     GTGCTAATTTTATAATGTTCATGTTGCAAATCTGTTATAAAAAGTCAGGA
AS_Tasmanian_Ssa03_sdY     GTGCTAATTTTATAATGTTCATGTTGCAAATCTGTTATAAAAAGTCAGGA
AS_European_sdY            GTGCTAATTTTATAATGTTCATGTTGCAAATCTGTTATAAAAAGTCAGGA
AS_Tasmanian_Ssa06_sdY     GTGCTAATTTTATAATGTTCATGTTGCAAATCTGTTATAAAAAGTCAGGA
Chinook_sdY                GTGCTCATT------GGATCATATCACAAATCTCTTATAAAAAGTCAGGA
RT_sdY                     GTGCTCATT------GGATCATATCACAAATCTCTTATAAAAAGTCAGGA
                           ***** ***       * **** *  ******* ****************

BT_sdY                     GCTGTTCTTCTGCGAGA---CTCATTGAAATTATTTTTTTCTCTCTCTCC
AS_Tasmanian_Ssa02_sdY     GCTGTTCTTCTGCGGGACTGCTCATTGAAATTTTGTTTTTCTCTCTCTCT
AS_Tasmanian_Ssa03_sdY     GCTGTTCTTCTGCGGGACTGCTCATTGAAATTTTGTTTTTCTCTCTCTCT
AS_European_sdY            GCTGTTCTTCTGCGAGACTGCTCATTGAAATTTTGTTTTTCTCTCTCTCT
AS_Tasmanian_Ssa06_sdY     GCTGTTCTTCTGCGAGACTGCTCATTGAAACTTTGTTTTTCTCTCTCTCT
Chinook_sdY                GCTGTTCTTCTGCATAACTGCTCATTGCAAGTA---TTTTTTATCTC---
RT_sdY                     GCTGTTCTTCTGCATAACTGCTCATTGCAAGTATTTTTTTTTATCTC---
                           *************   *   ******* ** *    **** * ****
```

```
BT_sdY                    CCC-----------------------------------------------
AS_Tasmanian_Ssa02_sdY    CCCTCTCTCCCCCCTCTCTCTCTCTCTCTCTCTCTCTCTCCCTAACTCTC
AS_Tasmanian_Ssa03_sdY    CCCTCTCTCCCCC--CTCTCTCTCTCTCTCTCTCTCTCTCCCTAACTCTC
AS_European_sdY           CCCTCTCTCCCCA-------------------------------------
AS_Tasmanian_Ssa06_sdY    CCCTCTCTCCCCC--------CCCTCTCTCTCTCTCTCTCCCTAACTCTC
Chinook_sdY               --------------------------------------------------
RT_sdY                    --------------------------------------------------

BT_sdY                    -----CCCTCTCTCTCTCTCTCTATTTCTCTGTCTCTCCCTAACTCTCTC
AS_Tasmanian_Ssa02_sdY    TCTCTCCCTCTCTCTCTCTCTTTCTCTCTCTCTCTCCCTCTCTCTCTC
AS_Tasmanian_Ssa03_sdY    TCTCTCCCTCTCTCTCTCTCTTTCTCTCTCTCTCTCCCTCTCTCTCTC
AS_European_sdY           -------------CTCTCTCTCTCTCTCTCTCTCTCTCCCTAACTCTCTC
AS_Tasmanian_Ssa06_sdY    TCTC-CCCTCTCTCTCTCTCTTTCTCTCTCTCTCTCTCCCTCTCTCTC
Chinook_sdY               --------------------------------------------------
RT_sdY                    --------------------------------------------------

BT_sdY                    TCTCCCTCTCTCTCTCT----------------------------CTCCC
AS_Tasmanian_Ssa02_sdY    TCTCTCTCTCTCTCTCTCTCTCTATCACTCTCCCTCTCTCTCTCTCCC
AS_Tasmanian_Ssa03_sdY    TCTCTCTCTCTCTCTCTCTCTCTATCACTCTCCCTCTCTCTCTCTCCC
AS_European_sdY           TCTCCCTCTCTCTCTCT---------------------------------
AS_Tasmanian_Ssa06_sdY    TCTCTCTCTCTCTCTCT----------CTCTCACTCTCCCTCTCTCTCTC
Chinook_sdY               --------------------------------------------------
RT_sdY                    --------------------------------------------------

BT_sdY                    TCTCTCTCTCTCTCTCCCTCCCTCTCTCTCTCTTTCTCTCTCTCTCTCTC
AS_Tasmanian_Ssa02_sdY    TCCCT--CTCTCTCTCTCTCTCTCTCTCTCTCTCTCTCTCTCTCTCTCTC
AS_Tasmanian_Ssa03_sdY    TCCCTCTCTCTCTCTCTCTCTCTCTCTCTCTCTCTCTCTCTCTCTCTCTC
AS_European_sdY           ---------------------------CTCTCTCTCTCTCTCTCTCTCTC
AS_Tasmanian_Ssa06_sdY    TCCCTCCCTCTCTCTCTCTCTCTCTCTCTCTCTCTCTCTCTCTCTCTCTC
Chinook_sdY               ---------------------------------CTCCCTCTA--------
RT_sdY                    ---------------------------------CTCCCTCTATCTCTGTC
```

```
                                                                              |----
BT_sdY                     TCCTCCCTCTCTTTCTATGTCCCTCTGTCACTCTCTCTCTCCCCAG|CCCA
AS_Tasmanian_Ssa02_sdY     TCCTCCCTCTCTTTCTATGTCCCTCTGTCACTCTCTCTCTCCCCAG|CCCA
AS_Tasmanian_Ssa03_sdY     TCCTCCCTCTCTTTCTATGTCCCTCTGTCACTCTCTCTCTCCCCAG|CCCA
AS_European_sdY            TCCTCCCTCTCTTTCTATGTCCCTCTGTCACTCTCTCTCTCCCCAG|CCCA
AS_Tasmanian_Ssa06_sdY     TCCTCCCTCTCTTTCTATGTCCCTCTGTCACTCTCTCTCTCCCCAG|CCCA
Chinook_sdY                -----------------------------------TCTCTCCCCAG|CCCA
RT_sdY                     TGTTCCCTCTGTT--------------------TCTCTCTCCCCAG|CCCA
                                                              ***********|****

                             exon2
                           --Exon 2F-------->
BT_sdY                     GCACTCTTTTCTTGTCTCAGTGGAGTACTGCGAAGAGGAGGTGCTTAGTC
AS_Tasmanian_Ssa02_sdY     GCACTCTTTTCTTGTCTCAGTGGAGTACTGCGAAGAGGAGGTGCTTAGTC
AS_Tasmanian_Ssa03_sdY     GCACTCTTTTCTTGTCTCAGTGGAGTACTGCGAAGAGGAGGTGCTTAGTC
AS_European_sdY            GCACTCTTTTCTTGTCTCAGTGGAGTACTGCGAAGAGGAGGTGCTTAGTC
AS_Tasmanian_Ssa06_sdY     GCACTCTTTTCTTGTCTCAGTGGAGTACTGCGAAGAGGAGGTGCTTAGTC
Chinook_sdY                GCACTGTTTTCTTGTCTCAGTGGAGTACTGCGAAGAGGAGGTGCTTAGTC
RT_sdY                     GCACTGTTTTCTTGTCTCAGTGGAGTACTGCGAAGAGGAGGTGCTTAGTC
                           ***** ********************************************

                             exon2
                                                                <----Exon 1R-
BT_sdY                     ATGAGGTTATGGGGAGTGATGTCAGAATTGCCTACAAGCCCTTCTCCCTG
AS_Tasmanian_Ssa02_sdY     ATGAGGTTATGGGGAGTGATGTCAGAATTGCCTACAAGCCCTTCTCCCTG
AS_Tasmanian_Ssa03_sdY     ATGAGGTTATGGGGAGTGATGTCAGAATTGCCTACAAGCCCTTCTCCCTG
AS_European_sdY            ATGAGGTTATGGGGAGTGATGTCAGAATTGCCTACAAGCCCTTCTCCCTG
AS_Tasmanian_Ssa06_sdY     ATGAGGTTATGGGGAGTGATGTCAGAATTGCCTACAAGCCCTTCTCCCTG
Chinook_sdY                ATGAGGTCATGGGGGGTGATGTCAGAATTGCCCACAAGAC---CTCCCTA
RT_sdY                     ATGAGGTCATGGGGGGTGATGTCAGAATTGCCCACAAGAC---CTCCCTA
                           ******* ****** ***************** ***** *   ****** 
```

```
                          exon2
                         -------
BT_sdY                   ATGATGGATGGGATCCCCGTCATCTCTCTCCCAAAGCCCCCCGACACCAT
AS_Tasmanian_Ssa02_sdY   ATGATGGATGGCATCCCCGTCATCTCTCTCCCAAAGCCCCCCGACACCAT
AS_Tasmanian_Ssa03_sdY   ATGATGGATGGCATCCCCGTCATCTCTCTCCCAAAGCCCCCCGACACCAT
AS_European_sdY          ATGATGGATGGGATCCCCGTCATCTCTCTCCCAAAGCCCCCCGACACCAT
AS_Tasmanian_Ssa06_sdY   ATGATGGATGGGATCCCCGTCATCTCTCTCCCAAAGCCCCCCGACACCAT
Chinook_sdY              ATGATGGATGGGATCCCCTTCATTTCTCTCCCAAAGCCCCCCAACACCCT
RT_sdY                   ATGATGGATGGGATCCCCTTCATCTCTCTCCCAAAGCCCCCCAACACCCT
                         *********** ****** **** ****************** ***** *

                          exon2
BT_sdY                   TCCCATCTCCTCTGACCGTTCAACCCTCTCCAACCTGCTTTCCCTCATGG
AS_Tasmanian_Ssa02_sdY   TCCCATCTCCTCTGACCGTTCAATCCTCTCCAACCTGCTTTCCCTCATGG
AS_Tasmanian_Ssa03_sdY   TCCCATCTCCTCTGACCGTTCAATCCTCTCCAACCTGCTTTCCCTCATGG
AS_European_sdY          TCCCATCTCCTCTGACCGTTCAATCCTCTCCAACCTGCTTTCCCTCATGG
AS_Tasmanian_Ssa06_sdY   TCCCATCTCCTCTGACCGTTCAATCCTCTCCAACCTGCTTTCCCTCATGG
Chinook_sdY              TCCTATCTCCTCTGATCGTTCAATCCTCTCCAACCTGTTGTCCCTCATGG
RT_sdY                   TCCTATCTCCTCTGATCGTTCAATCCTCTCCAACCTGTTGTCCCTCATGG
                         *** *********** ******* ************* * **********

                          exon2
BT_sdY                   AGGGTGGAGTGGTTTTAAGCTCTAGGGAGGAAGGCATCTATGCTGAACGG
AS_Tasmanian_Ssa02_sdY   AGGGTGGAGTGGTTTTAAGCTCTAAGGAGGAAGGCATCTATGCTGAACGG
AS_Tasmanian_Ssa03_sdY   AGGGTGGAGTGGTTTTAAGCTCTAAGGAGGAAGGCATCTATGCTGAACGG
AS_European_sdY          AGGGTGGAGTGGTTTTAAGCTCTAAGGAGGAAGGCATCTATGCTGAACGG
AS_Tasmanian_Ssa06_sdY   AGGGTGGAGTGGTTTTAAGCTCTAAGGAGGAAGGCATCTATGCTGAACGG
Chinook_sdY              AGGGTGGAGTAGTTTTAAGCTCTAGGGAGGAAGGTATCTATGCTGAACGG
RT_sdY                   AGGGTGGAGTGGTTTTAAGCTCTAGGGAGGAAGGTATCTATGCTGAACGG
                         ********** ************* ********* ***************
```

```
                              exon2
BT_sdY                      CATAGCCAAGCCATAGTCTCCTGGATGGGCGGCACGGGGGATGAGATGCA
AS_Tasmanian_Ssa02_sdY      CATAGCCAAGCCATAGTCTCCTGGATGGGCGGCACGGGGGATGAGATGCA
AS_Tasmanian_Ssa03_sdY      CATAGCCAAGCCATAGTCTCCTGGATGGGCGGCACGGGGGATGAGATGCA
AS_European_sdY             CATAGCCAAGCCATAGTCTCCTGGATGGGCGGCACGGGGGATGAGATGCA
AS_Tasmanian_Ssa06_sdY      CATAGCCAAGCCATAGTCTCCTGGATGGGCGGCACGGGGGATGAGATGCA
Chinook_sdY                 CATAGCCAAGCCACAGTCTCCTGGATGGGTGGCACCGGAGATGAGATGCA
RT_sdY                      CATAGCCAAGCCACAGTCTCCTGGATGGGTGGCACCGGAGATGAGATGCA
                            ************* *************** ***** ** ***********
                              exon2
                                                                   <--Exon 2R-
BT_sdY                      CGTGATGGAGCGTGATGTGGATCCTGTGATGCTCTTCAACAGGGAGACCT
AS_Tasmanian_Ssa02_sdY      CGTGATGGAGCGTGATGTGGATCCTGTGATGCTCTTCAACAGGGAGACCT
AS_Tasmanian_Ssa03_sdY      CGTGATGGAGCGTGATGTGGATCCTGTGATGCTCTTCAACAGGGAGACCT
AS_European_sdY             CGTGATGGAGCGTGATGTGGATCCTGTGATGCTCTTCAACAGGGAGACCT
AS_Tasmanian_Ssa06_sdY      CGTGATGGAGCGTGATGTGGATCCTGTGATGCTCTTCAACAGGGAGACCT
Chinook_sdY                 CGTGATGGACCGTGATGTGGACCCTGTGATGCTCTTCAACAGAGAGACCT
RT_sdY                      CGTGATGGAGCGTGATGTGGACCCTGTGATGCTCTTCAACAGAGAGCACT
                            ********* *********** ******************** ***  **

                            ---------|
BT_sdY                      TCAGACAGG|GTGAGTGA---------------------------------
AS_Tasmanian_Ssa02_sdY      TCAGACAGG|GTGAGTGA---------------------------------
AS_Tasmanian_Ssa03_sdY      TCAGACAGG|GTGAGTGA---------------------------------
AS_European_sdY             TCAGACAGG|GTGAGTGA---------------------------------
AS_Tasmanian_Ssa06_sdY      TCAGACAGG|GTGAGTGA---------------------------------
Chinook_sdY                 TCAGACAGG|GTGAGTGAACCTGTTAGTGGTAAATTCTAATGATTCTTGAA
RT_sdY                      TCAGACAGG|GTGAGTGAATCTGTTAGTGGTAAATTCTAATGATTCTTGAA
                            *********|********
```

```
BT_sdY                       --------------------------------------------------
AS_Tasmanian_Ssa02_sdY       --------------------------------------------------
AS_Tasmanian_Ssa03_sdY       --------------------------------------------------
AS_European_sdY              --------------------------------------------------
AS_Tasmanian_Ssa06_sdY       --------------------------------------------------
Chinook_sdY                  GAGCATAACTTATAATAGGGAGGATGTTCCCTCGGGGGGATCAAATCAGC
RT_sdY                       GAGCATAACTTATAATAG--------------------------------

BT_sdY                       --------------------------------------------------
AS_Tasmanian_Ssa02_sdY       --------------------------------------------------
AS_Tasmanian_Ssa03_sdY       --------------------------------------------------
AS_European_sdY              --------------------------------------------------
AS_Tasmanian_Ssa06_sdY       --------------------------------------------------
Chinook_sdY                  GGGTAGTCAGCGGATATTTTAGAGCGTCACTGATCGTTTTTCGATAAAAT
RT_sdY                       --------------------------------------------------

BT_sdY                       --------------------------------------------------
AS_Tasmanian_Ssa02_sdY       --------------------------------------------------
AS_Tasmanian_Ssa03_sdY       --------------------------------------------------
AS_European_sdY              --------------------------------------------------
AS_Tasmanian_Ssa06_sdY       --------------------------------------------------
Chinook_sdY                  TCAAACTTTCATTAAAACACACATGCAAGGTATTGAATTAAAGCTACACT
RT_sdY                       --------------------------------------------------

BT_sdY                       --------------------------------------------------
AS_Tasmanian_Ssa02_sdY       --------------------------------------------------
AS_Tasmanian_Ssa03_sdY       --------------------------------------------------
AS_European_sdY              --------------------------------------------------
AS_Tasmanian_Ssa06_sdY       --------------------------------------------------
Chinook_sdY                  CGTTGTGAATCTAGTCAGATTTGTAAAATGCTTTTCGGCGAAAGCATGAG
RT_sdY                       --------------------------------------------------
```

```
BT_sdY                    --------------------------------------------------
AS_Tasmanian_Ssa02_sdY    --------------------------------------------------
AS_Tasmanian_Ssa03_sdY    --------------------------------------------------
AS_European_sdY           --------------------------------------------------
AS_Tasmanian_Ssa06_sdY    --------------------------------------------------
Chinook_sdY               AAGCTATTATCTGATAGCATGTAACACCCCAAAAGACCCGTAGAGGATGT
RT_sdY                    --------------------------------------------------

BT_sdY                    --------------------------------------------------
AS_Tasmanian_Ssa02_sdY    --------------------------------------------------
AS_Tasmanian_Ssa03_sdY    --------------------------------------------------
AS_European_sdY           --------------------------------------------------
AS_Tasmanian_Ssa06_sdY    --------------------------------------------------
Chinook_sdY               AAACAAAAATAATTAGCGTAGTCGGCGCTACACAAACCGCACAATAAAAT
RT_sdY                    --------------------------------------------------

BT_sdY                    --------------------------------------------------
AS_Tasmanian_Ssa02_sdY    --------------------------------------------------
AS_Tasmanian_Ssa03_sdY    --------------------------------------------------
AS_European_sdY           --------------------------------------------------
AS_Tasmanian_Ssa06_sdY    --------------------------------------------------
Chinook_sdY               ATAAAACATTCATTACCTTTGACCATCTTCTTTCTTGGCACTCCTTGATG
RT_sdY                    --------------------------------------------------

BT_sdY                    --------------------------------------------------
AS_Tasmanian_Ssa02_sdY    --------------------------------------------------
AS_Tasmanian_Ssa03_sdY    --------------------------------------------------
AS_European_sdY           --------------------------------------------------
AS_Tasmanian_Ssa06_sdY    --------------------------------------------------
Chinook_sdY               TCCCATAATCAATACTGGGTCTTTTTTTGGATTAAATCGGTCCATATATA
RT_sdY                    --------------------------------------------------
```

```
BT_sdY                   --------------------------------------------------
AS_Tasmanian_Ssa02_sdY   --------------------------------------------------
AS_Tasmanian_Ssa03_sdY   --------------------------------------------------
AS_European_sdY          --------------------------------------------------
AS_Tasmanian_Ssa06_sdY   --------------------------------------------------
Chinook_sdY              GCCTAGATATCGATCTATGAAGACTGTGTGATAAACGGAAAAAAATAGCG
RT_sdY                   --------------------------------------------------

BT_sdY                   --------------------------------------------------
AS_Tasmanian_Ssa02_sdY   --------------------------------------------------
AS_Tasmanian_Ssa03_sdY   --------------------------------------------------
AS_European_sdY          --------------------------------------------------
AS_Tasmanian_Ssa06_sdY   --------------------------------------------------
Chinook_sdY              TTTCATAACGTAACGTCATTTTTTAAAAGTTAAAAAGTCGACGATAAACT
RT_sdY                   --------------------------------------------------

BT_sdY                   --------------------------------------------------
AS_Tasmanian_Ssa02_sdY   --------------------------------------------------
AS_Tasmanian_Ssa03_sdY   --------------------------------------------------
AS_European_sdY          --------------------------------------------------
AS_Tasmanian_Ssa06_sdY   --------------------------------------------------
Chinook_sdY              TTCACAAAACACTTCGAAATACCTTTCTAATGCAACTTTAGGTATTACTA
RT_sdY                   --------------------------------------------------

BT_sdY                   --------------------------------------------------
AS_Tasmanian_Ssa02_sdY   --------------------------------------------------
AS_Tasmanian_Ssa03_sdY   --------------------------------------------------
AS_European_sdY          --------------------------------------------------
AS_Tasmanian_Ssa06_sdY   --------------------------------------------------
Chinook_sdY              CACGTTAATAAGCTATAAAAATCATCAGGAGGCGATGTAAATTCGATAGT
RT_sdY                   --------------------------------------------------
```

```
BT_sdY                        --------------------------------------------------
AS_Tasmanian_Ssa02_sdY        --------------------------------------------------
AS_Tasmanian_Ssa03_sdY        --------------------------------------------------
AS_European_sdY               --------------------------------------------------
AS_Tasmanian_Ssa06_sdY        --------------------------------------------------
Chinook_sdY                   TGGCGTGTGGAAAAAATGTCCGGAAAAACACAGAGACAATGCCTCAGGTC
RT_sdY                        --------------------------------------------------

BT_sdY                        --------------------------------------------------
AS_Tasmanian_Ssa02_sdY        --------------------------------------------------
AS_Tasmanian_Ssa03_sdY        --------------------------------------------------
AS_European_sdY               --------------------------------------------------
AS_Tasmanian_Ssa06_sdY        --------------------------------------------------
Chinook_sdY                   GGTGGTCGGAGGGAATCGGTTCCCTTTGGTCGGATCTACCAAGAATCAAA
RT_sdY                        --------------------------------------------------

BT_sdY                        --------------------------------------------------
AS_Tasmanian_Ssa02_sdY        --------------------------------------------------
AS_Tasmanian_Ssa03_sdY        --------------------------------------------------
AS_European_sdY               --------------------------------------------------
AS_Tasmanian_Ssa06_sdY        --------------------------------------------------
Chinook_sdY                   TCAGAATCAAATGAGGAGACTCTATACATCCTGTGGAAGCTGTAGGTACT
RT_sdY                        --------------------------------------------------

BT_sdY                        --------------------------------------------------
AS_Tasmanian_Ssa02_sdY        --------------------------------------------------
AS_Tasmanian_Ssa03_sdY        --------------------------------------------------
AS_European_sdY               --------------------------------------------------
AS_Tasmanian_Ssa06_sdY        --------------------------------------------------
Chinook_sdY                   GCAAGCTCGGCCTCATTTAATACGGTTCACCTTTAACAATTCATGGAAGT
RT_sdY                        --------------------------------------------------
```

```
BT_sdY                    --------------------------------------------------
AS_Tasmanian_Ssa02_sdY    --------------------------------------------------
AS_Tasmanian_Ssa03_sdY    --------------------------------------------------
AS_European_sdY           --------------------------------------------------
AS_Tasmanian_Ssa06_sdY    --------------------------------------------------
Chinook_sdY               GGCGCATGGATATTTTTTTCCATCTCCAGTKATCAGATTTTCCTGCGCTT
RT_sdY                    --------------------------------------------------

BT_sdY                    --------------------------------------------------
AS_Tasmanian_Ssa02_sdY    --------------------------------------------------
AS_Tasmanian_Ssa03_sdY    --------------------------------------------------
AS_European_sdY           --------------------------------------------------
AS_Tasmanian_Ssa06_sdY    --------------------------------------------------
Chinook_sdY               TTCGATGAAACAGACGTTCTGTTATAGTCACAGCCGTGATTTAAACAGTT
RT_sdY                    --------------------------------------------------

BT_sdY                    --------------------------------------------------
AS_Tasmanian_Ssa02_sdY    --------------------------------------------------
AS_Tasmanian_Ssa03_sdY    --------------------------------------------------
AS_European_sdY           --------------------------------------------------
AS_Tasmanian_Ssa06_sdY    --------------------------------------------------
Chinook_sdY               TTAGAAACGTCTGAGTGTTTTCTATCCACACATACTAATCATATGCATAT
RT_sdY                    --------------------------------------------------

BT_sdY                    --------------------------------------------------
AS_Tasmanian_Ssa02_sdY    --------------------------------------------------
AS_Tasmanian_Ssa03_sdY    --------------------------------------------------
AS_European_sdY           --------------------------------------------------
AS_Tasmanian_Ssa06_sdY    --------------------------------------------------
Chinook_sdY               ACTATATTCCTGGAATGAGTAGCAGGGCGCTGAAATGTTGCGCGATTTTT
RT_sdY                    --------------------------------------------------
```

```
BT_sdY                     --------------------------------------------------
AS_Tasmanian_Ssa02_sdY     --------------------------------------------------
AS_Tasmanian_Ssa03_sdY     --------------------------------------------------
AS_European_sdY            --------------------------------------------------
AS_Tasmanian_Ssa06_sdY     --------------------------------------------------
Chinook_sdY                AACAAAAAGCTGCGAAAATTCGCAGCCTCCCCAAGAGGTTTTAATTAAAC
RT_sdY                     --------------------------------------------TTAAAC

BT_sdY                     --------------------------------------------------
AS_Tasmanian_Ssa02_sdY     --------------------------------------------------
AS_Tasmanian_Ssa03_sdY     --------------------------------------------------
AS_European_sdY            --------------------------------------------------
AS_Tasmanian_Ssa06_sdY     --------------------------------------------------
Chinook_sdY                AACCCAAAATATAAGCTTATTTAAATCCTATGTTTGTGAACTTTGTAAAT
RT_sdY                     AACCCAAAATATAAGCTTGTTTAAATCCTATGTTTGTGAACTTTGTAAAT

BT_sdY                     --------------------------------------------------
AS_Tasmanian_Ssa02_sdY     --------------------------------------------------
AS_Tasmanian_Ssa03_sdY     --------------------------------------------------
AS_European_sdY            --------------------------------------------------
AS_Tasmanian_Ssa06_sdY     --------------------------------------------------
Chinook_sdY                GTAAACAAACACTATATAGCCTTAAAAATGTTACTTATGTGATAATGCCT
RT_sdY                     GTAAACAAACACTATATAGCCTTAAAAATGTTACTTATGTGATAATGCCT

BT_sdY                     --------------------------------------------------
AS_Tasmanian_Ssa02_sdY     --------------------------------------------------
AS_Tasmanian_Ssa03_sdY     --------------------------------------------------
AS_European_sdY            --------------------------------------------------
AS_Tasmanian_Ssa06_sdY     --------------------------------------------------
Chinook_sdY                GAAAAAATGGTGTTTGGAGGGTATATTGGCACAGTGTTGTTAGGCCTATA
RT_sdY                     GAAAAAACAGTGTTTGGAGGGTATATTGGCGCAGTGTTGTTAGGCCAATA
```

```
BT_sdY                      ------------------------------------------------TA
AS_Tasmanian_Ssa02_sdY      ------------------------------------------------TA
AS_Tasmanian_Ssa03_sdY      ------------------------------------------------TA
AS_European_sdY             ------------------------------------------------TA
AS_Tasmanian_Ssa06_sdY      ------------------------------------------------TA
Chinook_sdY                 ATTTTGATAAATCTATTCATACTATTTCATCCTTCTATTTCATCCTTCTA
RT_sdY                      ATTTTGATAAATGTATTCATAC--------------------------TA
                                                                            **

BT_sdY                      TG------------------------------------------------
AS_Tasmanian_Ssa02_sdY      TT------------------------------------------------
AS_Tasmanian_Ssa03_sdY      TT------------------------------------------------
AS_European_sdY             TT------------------------------------------------
AS_Tasmanian_Ssa06_sdY      TT------------------------------------------------
Chinook_sdY                 TTTCATCCTTCCACAAGATATAGTCCCGACACAACCATATGGTTGCTACC
RT_sdY                      TTTCATCCTTCCACAAGATATAGTCCCGACACAACCATATGGTTGCTACC
                            *

BT_sdY                      --------------------------------------------------
AS_Tasmanian_Ssa02_sdY      --------------------------------------------------
AS_Tasmanian_Ssa03_sdY      --------------------------------------------------
AS_European_sdY             --------------------------------------------------
AS_Tasmanian_Ssa06_sdY      --------------------------------------------------
Chinook_sdY                 CAAACTGGATGGTCATCCATTGGTTTTGGTTGCCAAAGACACAACCCAGT
RT_sdY                      CAAACTGGATGGTCATCCATTGG-TTTGGTTGCCAAAGACGCTACCCAGT

BT_sdY                      --------------------------------------------------
AS_Tasmanian_Ssa02_sdY      --------------------------------------------------
AS_Tasmanian_Ssa03_sdY      --------------------------------------------------
AS_European_sdY             --------------------------------------------------
AS_Tasmanian_Ssa06_sdY      --------------------------------------------------
Chinook_sdY                 TGTTCAGATTTTTTGTTCTGTATCTATGGACACGACCTGGTTGTTCATTT
RT_sdY                      TGTTCAGATTTTTTGTTCTGTAACTATGGACACGACCCGGTTGTTCATTT
```

```
BT_sdY                      --------------------------------------------------
AS_Tasmanian_Ssa02_sdY      --------------------------------------------------
AS_Tasmanian_Ssa03_sdY      --------------------------------------------------
AS_European_sdY             --------------------------------------------------
AS_Tasmanian_Ssa06_sdY      --------------------------------------------------
Chinook_sdY                 TAAATGTTCCATTGCCATTCTGGCTGGCAACATTCTTATCCTTTGCTTGC
RT_sdY                      TAAATGTTCCATTGCCATTCTGGCTGGCAACATTCTTATCCCTTG-TTGC

BT_sdY                      --------------------------------------------------
AS_Tasmanian_Ssa02_sdY      --------------------------------------------------
AS_Tasmanian_Ssa03_sdY      --------------------------------------------------
AS_European_sdY             --------------------------------------------------
AS_Tasmanian_Ssa06_sdY      --------------------------------------------------
Chinook_sdY                 TAGCTAGCCAACTGCGGCTAACTTACAGTTACGTCAAACAGTGCAGTCAG
RT_sdY                      TAGCTAGTCAACTATGGCTAACTTACAGTTACGTCAAACAGTGCAGTGAG

BT_sdY                      --------------------------------------------------
AS_Tasmanian_Ssa02_sdY      --------------------------------------------------
AS_Tasmanian_Ssa03_sdY      --------------------------------------------------
AS_European_sdY             --------------------------------------------------
AS_Tasmanian_Ssa06_sdY      --------------------------------------------------
Chinook_sdY                 CAACAAAGTATATTGCATTTGCATTTGTTTAAGATGTTTTCTAATGACAT
RT_sdY                      CACAAGTATATGCATTTGCATTGTTAG------------ACTTTTCTAAT

BT_sdY                      --------------------------------------------------
AS_Tasmanian_Ssa02_sdY      --------------------------------------------------
AS_Tasmanian_Ssa03_sdY      --------------------------------------------------
AS_European_sdY             --------------------------------------------------
AS_Tasmanian_Ssa06_sdY      --------------------------------------------------
Chinook_sdY                 TTATTTGGATATAACAATGACTTACACAGTTTGGCCTGCCATAGAAAATG
RT_sdY                      TACATTTATTGGATGTAACATGACTCATGGTTGGCCTGCCATAGAAAATG
```

```
BT_sdY                     --------------------------------------------------
AS_Tasmanian_Ssa02_sdY     --------------------------------------------------
AS_Tasmanian_Ssa03_sdY     --------------------------------------------------
AS_European_sdY            --------------------------------------------------
AS_Tasmanian_Ssa06_sdY     --------------------------------------------------
Chinook_sdY                TTCTCTCTCATCAGGACACTGTTTACAGAGGAG-----CAACAACACAGC
RT_sdY                     TTCTCTCTCGTCAGGACACTGTTTACAGAGGAGCTAGTCAACAATACAGC

BT_sdY                     ----------------AAGGGAATCTG-----------------------
AS_Tasmanian_Ssa02_sdY     ----------------AAGGGAATCTG-----------------------
AS_Tasmanian_Ssa03_sdY     ----------------AAGGGAATCTG-----------------------
AS_European_sdY            ----------------AAGGGAATCTG-----------------------
AS_Tasmanian_Ssa06_sdY     ----------------AAGGGAATCTG-----------------------
Chinook_sdY                TAACACAATCACTTCAAACGGAAGCTGAAAAGACTGAAAACTGGCTGCAC
RT_sdY                     TAACACAATCACTTCAAACGGAAGCTGGAAAGACTCAAAACTGGCTGCAC
                                           ** **** ***
BT_sdY                     --------------------------------------------------
AS_Tasmanian_Ssa02_sdY     --------------------------------------------------
AS_Tasmanian_Ssa03_sdY     --------------------------------------------------
AS_European_sdY            --------------------------------------------------
AS_Tasmanian_Ssa06_sdY     --------------------------------------------------
Chinook_sdY                TTTGTTTCCTTTTACCTGTTTTTTATTGATATTTTTTGTATATACAGTAT
RT_sdY                     TTTGTTTCCTTTTACCTGTTTTTTATTGATATTTTTTGTATATAGAGTAT

BT_sdY                     --------------------------------------------------
AS_Tasmanian_Ssa02_sdY     --------------------------------------------------
AS_Tasmanian_Ssa03_sdY     --------------------------------------------------
AS_European_sdY            --------------------------------------------------
AS_Tasmanian_Ssa06_sdY     --------------------------------------------------
Chinook_sdY                CCATAAAAATTATGCTGATTCATGATTTTGACTGGCTGAGAAAAGCTGCC
RT_sdY                     CCATAAAAATTATGCTGATTCGTGATTTTGACTGGCTGAGAAAAGCTGCC
```

```
BT_sdY                      --------------------------------------------------
AS_Tasmanian_Ssa02_sdY      --------------------------------------------------
AS_Tasmanian_Ssa03_sdY      --------------------------------------------------
AS_European_sdY             --------------------------------------------------
AS_Tasmanian_Ssa06_sdY      --------------------------------------------------
Chinook_sdY                 AGCCTATCTGTCTCTTCCCGACTCCCGACACATTCGTTACTATGGGACAG
RT_sdY                      TGCCTATCTGTCTCTTCCCGACTCCCGACACATTCGTTACTATGGGACAG

BT_sdY                      --------------------------------------------------
AS_Tasmanian_Ssa02_sdY      --------------------------------------------------
AS_Tasmanian_Ssa03_sdY      --------------------------------------------------
AS_European_sdY             --------------------------------------------------
AS_Tasmanian_Ssa06_sdY      --------------------------------------------------
Chinook_sdY                 CTGGAGAAATAATTTGAATTTTGAAACAATGTTGCAATATATCAGAGAGA
RT_sdY                      CTAGAGAAATAATTGGAATTTTGAAACAATGTTCCAATATATCAGAGAGA

BT_sdY                      --------------------------------------------------
AS_Tasmanian_Ssa02_sdY      --------------------------------------------------
AS_Tasmanian_Ssa03_sdY      --------------------------------------------------
AS_European_sdY             --------------------------------------------------
AS_Tasmanian_Ssa06_sdY      --------------------------------------------------
Chinook_sdY                 CAGATAGTAAGGGTTATACAAATCTCCGCTGTTTAAAACTAAATGTTAGT
RT_sdY                      CAGATAGTAAGGTTTATACAAATCTCCGCTGTTTAAAACTAAA-------

BT_sdY                      --------------------------------------------------
AS_Tasmanian_Ssa02_sdY      --------------------------------------------------
AS_Tasmanian_Ssa03_sdY      --------------------------------------------------
AS_European_sdY             --------------------------------------------------
AS_Tasmanian_Ssa06_sdY      --------------------------------------------------
Chinook_sdY                 CTAAATGGAATGTGAGATAATGTCTAGATGCTTTTAATATTGGAGGTCAA
RT_sdY                      --------------------------------------------------
```

```
BT_sdY                     --------------------------------------------------
AS_Tasmanian_Ssa02_sdY     --------------------------------------------------
AS_Tasmanian_Ssa03_sdY     --------------------------------------------------
AS_European_sdY            --------------------------------------------------
AS_Tasmanian_Ssa06_sdY     --------------------------------------------------
Chinook_sdY                GTTTATACATTGCCTGGCTGGGCTGATGAGACAGTGGATTGTGCTGTCAG
RT_sdY                     -TTTATACATTGCCTGGCTGGGCTGATGAGACAGTGGATTGTGCTGTCAG

BT_sdY                     --------------------------------------------------
AS_Tasmanian_Ssa02_sdY     --------------------------------------------------
AS_Tasmanian_Ssa03_sdY     --------------------------------------------------
AS_European_sdY            --------------------------------------------------
AS_Tasmanian_Ssa06_sdY     --------------------------------------------------
Chinook_sdY                ATGGAACAGAGTAAATAGGCATTTTAACAACATAGATTTAGCTGGTAGTA
RT_sdY                     ATGGAGCAGAGTAAATAGGCATTTTACATAGATAGATTTAGCTGGTAGTA

BT_sdY                     --------------------------------------------------
AS_Tasmanian_Ssa02_sdY     --------------------------------------------------
AS_Tasmanian_Ssa03_sdY     --------------------------------------------------
AS_European_sdY            --------------------------------------------------
AS_Tasmanian_Ssa06_sdY     --------------------------------------------------
Chinook_sdY                ACTTGTGGAATAGATACTGGCTTGAATGCATTTTTAACCAATCAGCATTC
RT_sdY                     ACTTGTGGAATAGATACTGGCTTGAATGCATTTTTAACCCATCAGCATTC

BT_sdY                     --------------------------------------------------
AS_Tasmanian_Ssa02_sdY     --------------------------------------------------
AS_Tasmanian_Ssa03_sdY     --------------------------------------------------
AS_European_sdY            --------------------------------------------------
AS_Tasmanian_Ssa06_sdY     --------------------------------------------------
Chinook_sdY                ATGATTACACACACACGTTGTATAAAACGTATATTGCCAACGATAATGTT
RT_sdY                     ATGATTACACCCACACGTTGTATAAAACGTATATTGCCAACTATAATGTT
```

```
BT_sdY                      ----TTACGGGTAAT------TCACAACAGATTCCACGAAACACTAATTG
AS_Tasmanian_Ssa02_sdY      ----TTACGGGTAAT------TCACAACAGATTTCACGAAACACTAATTG
AS_Tasmanian_Ssa03_sdY      ----TTACGGGTAAT------TCACAACAGATTTCACGAAACACTAATTG
AS_European_sdY             ----TTACGGGTAAT------TCACAACAGATTTCACGAAACACTAATTG
AS_Tasmanian_Ssa06_sdY      ----TTACGGGTAAT------TCACAACAGATTTCACGAAACACTAATTG
Chinook_sdY                 GATATTATGGTTAGTCAGTCCTCACAACAGGTTTCACAAAACACTAAGTG
RT_sdY                      GATATTATGGTTAGTCAGTCCTCACAACAGGTTTCACAAAACACTAAGTG
                                *** ** ** *      ********* ** *** ********* **

BT_sdY                      TGTGCCCTCAGGCCCCTACTCCGCTACCACGTAACTACAACACAAAATCA
AS_Tasmanian_Ssa02_sdY      TGTGCCCTCAGACCCTTACTCCGCTACCACGTAACTACAACACAAAATCA
AS_Tasmanian_Ssa03_sdY      TGTGCCCTCAGACCCTTACTCCGCTACCACGTAACTACAACACAAAATCA
AS_European_sdY             TGTGCCCTCAGACCCTTACTCCGCTACCACGTAACTACAACACAAAATCA
AS_Tasmanian_Ssa06_sdY      TGTGCCCTCAGACCCTTACTCCGCTACCACGTAACTACAACACAAAATCA
Chinook_sdY                 TGTGCCCTCAGACCCCTACTCCACTACCCTATATCTACAACACAAAATSC
RT_sdY                      TGTGCCCTCAGACCCCTACTCCACTACCCTATATCTACAACACAAAATCC
                            *********** *** ****** *****   ** **************

BT_sdY                      ATGTGTAAATTTTTGTATTGTGCGTATGTTATCTTGTGTGTGTATGCGTG
AS_Tasmanian_Ssa02_sdY      ATGTGTACATTTTTGTATTGTGCGTATGTTATCTTGTGTGTGTATGCATG
AS_Tasmanian_Ssa03_sdY      ATGTGTACATTTTTGTATTGTGCGTATGTTATCTTGTGTGTGTATGCATG
AS_European_sdY             ATGTGTACATTTTTGTATTGTGCGTATGTTATCTTGTGTGTGTATGCATG
AS_Tasmanian_Ssa06_sdY      ATGTGTACATTTTTGTATTGTGCGTATGTTATCTTGTGTGTGTATGCATG
Chinook_sdY                 ATGTGTACGTGTGTGTATTGTGAGTATGTTATCGTGTGCGTTTATGCATG
RT_sdY                      ATGTGTACGTGTGTGTATTGTGAGTATGTTATTGTGTGCGTTTATGCATG
                            *******  * * ********* *********  **** ** ***** **
```

K. P. Lubieniecki *et al.*

```
BT_sdY                    TGTCGGTGCCTATGATTGTGTTGCTTCACAGACCCCGCTGTTCCATAATG
AS_Tasmanian_Ssa02_sdY    TGTCGGTGCCTATGATTGTGTTGCTTCACAGACCCCGCTGTTCCATAATG
AS_Tasmanian_Ssa03_sdY    TGTCGGTGCCTATGATTGTGTTGCTTCACAGACCCCGCTGTTCCATAATG
AS_European_sdY           TGTCGGTGCCTATGATTGTGTTGCTTCACAGACCCCGCTGTTCCATAATG
AS_Tasmanian_Ssa06_sdY    TGTCGGTGCCTATGATTGTGTTGCTTCACAGACCCCGCTGTTCCATAATG
Chinook_sdY               TGTCAGTGCCTATSTTTGTGTTGCTTCACAGTCCCCGCTGTTCCATAAGG
RT_sdY                    TGTCAGTACCTATGTTTGTGTTGCTTCACAGTCCCCGCTGTTCCATAAGG
                          *** ** *****   **************** **************** *

BT_sdY                    TGTATTTTT------------------ATCTGATTTTATTGA-TGCATCA
AS_Tasmanian_Ssa02_sdY    TGTATTTTT------------------ATCTGATTTTATTGC-TGCATCA
AS_Tasmanian_Ssa03_sdY    TGTATTTTT------------------ATCTGATTTTATTGC-TGCATCA
AS_European_sdY           TGTATTTTT------------------ATCTGATTTTATTGC-TGCATCA
AS_Tasmanian_Ssa06_sdY    TGTATTTTT------------------ATCTGATTTTATTGC-TGCATCA
Chinook_sdY               TGTATTTTTTAAATCTTTTTTTTTT---ATCTGATTCT--TGCATTTATCA
RT_sdY                    TGTATTTTTTTATCTGTTTTTTTTTTAAATCTGATTCTACTGCTTGCATCA
                          *********                  ******** *  **  *  ****

BT_sdY                    GTTACC-------TGATGTGGAATAGTGTTCCATGTAGTCATGGCTCTAT
AS_Tasmanian_Ssa02_sdY    GTTACC-------TGATGTGGAATAGAGTTCCATGTAGTCATGGCTCTAT
AS_Tasmanian_Ssa03_sdY    GTTACC-------TGATGTGGAATAGAGTCCCATGTAGTCATGGCTCTGT
AS_European_sdY           GTTACC-------TGATGTGGAATAGAGTTCCATGTAGTCATGGCTCTAT
AS_Tasmanian_Ssa06_sdY    GTTACC-------TGATGTGGAATAGAGTTCCATGTAGTCATGGCTCTAT
Chinook_sdY               TTTACCACACATTTTATGTGGAATAGAGTTCCATGTAGTCATGGCTCTAT
RT_sdY                    TTTACC-------TGATGTGGAATAAAGTTCCATGTAGCCATGGCTCTAT
                           *****       * **********  ** ******** ********* *
```

```
BT_sdY                     GTAGTACTGTGCTCCTCCCATAG-----TCTGTTCTGGACTTGGGGACTG
AS_Tasmanian_Ssa02_sdY     ATAGTACTGTGCTCCTCCCATAA-----TCTGTTCTGGACTTGAGGACTG
AS_Tasmanian_Ssa03_sdY     ATAGTACTGTGCTCCTCCCATAA-----TCTGTTCTGGACTTGAGGACTG
AS_European_sdY            ATAGTACTGTGCTCCTCCCATAA-----TCTGTTCTGGACTTGAGGACTG
AS_Tasmanian_Ssa06_sdY     ATAGTACTGTGCTCCTCCCATA------TCTGTTCTGGACTTGAGGACTG
Chinook_sdY                GTAGTAATGATTGCCTCTCATGGTCTGTTCTGTTCTGGACTTGGGGACTG
RT_sdY                     GTAGTAATGATCGCCTCCCATGG-----TCTGTTCTGGACTTGGGGACTG
                            ***** **    **** ***       *************** ******

BT_sdY                     TGAAGAGACCTCTGGTGACATGTCTTGTGGGGTATGGATGGGTGTCTGAG
AS_Tasmanian_Ssa02_sdY     TGAAGAGACCTCTGGTGGCATGTCTTGTGGGGTA----------------
AS_Tasmanian_Ssa03_sdY     TGAAGAGACCTCTGGTGGCATGTCTTGTGGGGTA----------------
AS_European_sdY            TGAAGAGACCTCTGGTGGCATGTCTTGTGGGGTA----------------
AS_Tasmanian_Ssa06_sdY     TGAAGAGACCTCTGGTGGCATGTCTTGTGGGGTA----------------
Chinook_sdY                TGAAGAGACCTCTGGTGGCATGTTTTG-----------------------
RT_sdY                     TGAAGAGACCTCTGGTGGCATGTTTTG-----------------------
                           ***************** ***** ***

BT_sdY                     CTGTGAGCCAGTAGTTTAAACAGACCTCTGGTGGCATGTCTTGTGGGGTA
AS_Tasmanian_Ssa02_sdY     --------------------------------------------------
AS_Tasmanian_Ssa03_sdY     --------------------------------------------------
AS_European_sdY            --------------------------------------------------
AS_Tasmanian_Ssa06_sdY     --------------------------------------------------
Chinook_sdY                --------------------------------------------------
RT_sdY                     --------------------------------------------------
```

```
BT_sdY                      TGGATGGGTGTCTGAGCTGTGAGCCAGTAGTTTAAACAGACCTCTGGTGA
AS_Tasmanian_Ssa02_sdY      --------------------------------------------------
AS_Tasmanian_Ssa03_sdY      --------------------------------------------------
AS_European_sdY             --------------------------------------------------
AS_Tasmanian_Ssa06_sdY      --------------------------------------------------
Chinook_sdY                 --------------------------------------------------
RT_sdY                      --------------------------------------------------

BT_sdY                      CATGTCTTGTGGGGTATGGATGGGTGTCTGAGCTGTGTGCCAGTAGTTCA
AS_Tasmanian_Ssa02_sdY      ---------TGGGGTATGCATGGGTGTCCGAGCTGTGTGCCAGTAGTTCA
AS_Tasmanian_Ssa03_sdY      ---------TGGGGTATGCACGGGTGTCCGAGCTGTGTGCCAGTAGTTCA
AS_European_sdY             ---------TGGGGTATGCATGGGTGTCCGAGCTGTGTGCCAGTAGTTCA
AS_Tasmanian_Ssa06_sdY      ---------TGGGGTATGCGTGGGTGTCCGAGCTGTGTGCCAGTAGTTCA
Chinook_sdY                 ---------TGAGTTTTGCATGGGTGTCCACGCTGTGTGCCAGTTGTTCA
RT_sdY                      ---------TGAGTTTTGCAT--GTGTCCACGCTGTGTGCCAGTTGTTCA
                                     ** * * **     *****   ************* *****

BT_sdY                      AACAGACAGTTCAGTACATTCAACATGTCAGTACATTTCACAAATACAAG
AS_Tasmanian_Ssa02_sdY      AACAGACAATTCAGTACATTCAACATGTCAGTACATTTCACAAATACAAG
AS_Tasmanian_Ssa03_sdY      AACAGACAATTCAGTACATTCAACATGTCAGTACATTTCACAAATACAAG
AS_European_sdY             AACAGACAATTCAGTACATTCAACATGTCAGTACATTTCACAAATACAAG
AS_Tasmanian_Ssa06_sdY      AACAGACAATTCAGTACATTCAACATGTCAGTACATTTCACAAATACAAG
Chinook_sdY                 AACAGACAGCTTGGTACATTCAACATTTCAATACATTTCATAAATAAAAG
RT_sdY                      AACAGACAGCTTGGTACATTCAACATTTCAATACATTTCATAAATACAAG
                            ********  *  ************* *** ********* ***** ***
```

```
BT_sdY                      TAGTGATGAAGTCAATCTCTCCTCCACTTTGAGCCATGAGAGATTTACAT
AS_Tasmanian_Ssa02_sdY      TAGTGATGAAGTCAATCTCTCCTCCACTTTGAGCCATGAGAGATTTACAT
AS_Tasmanian_Ssa03_sdY      TAGTGATGAAGTCAATCTCTCCTCCACTTTGAGCCATGAGAGATTTACAT
AS_European_sdY             TAGTGATGAAGTCAATCTCTCCTCCACTTTGAGCCATGAGAGATTTACAT
AS_Tasmanian_Ssa06_sdY      TAGTGATGAAGTCAATCTCTCCTCCACTTTGAGCCATGAGAGATTTACAT
Chinook_sdY                 TAGCGATGAAGTCAATCTCTCCTCTGCTTTGAGCCTGGAGAGATTGACAT
RT_sdY                      TAGCAATGAAGTCAATCTCTCCTCTGCTTTGAGCCTGGAGAGATTGACAT
                            ***  *******************  *********  ******** ****

BT_sdY                      GCATATTATTAATGTTAGCTCTCCATGTACATTTAAGGGCCAGCCGTGCT
AS_Tasmanian_Ssa02_sdY      GCATATTATTAATGTTAGCTCTC--TCTACATTTAAGG----GCCGTGCT
AS_Tasmanian_Ssa03_sdY      GCATATTATTAATGTTAGCTCTC--TCTACATTTAAGG----GCCGTGCT
AS_European_sdY             GCATATTATTAATGTTAGCTCTC--TCTACATTTAAGG----GCCGTGCT
AS_Tasmanian_Ssa06_sdY      GCATATTATTAATGTTAGCTCTC--TCTACATTTAAGG----GCCGTGCT
Chinook_sdY                 GCACAT-----ATATCAGCT---------GATGTAAGA------AGGGCT
RT_sdY                      GCATAT-----ATATCAGCT---------GATGTAAGA------AGGGCT
                            *** **     ** * ****          ** ****        * ***

BT_sdY                      GCCCTGTTCTAGGCCAGTTGTTATTTTCCCAAGTCCCTCTTTGTGGAGAG
AS_Tasmanian_Ssa02_sdY      GCCCTGTTCTAGGCCAGTTGTTATTTTCCCAAGTCCCTCTTTGTGGAGAG
AS_Tasmanian_Ssa03_sdY      GCCCTGTTCTAGGCCAGTTGTTATTTTCCCAAGTCCCTCTTTGTGGAGAG
AS_European_sdY             GCCCTGTTCTAGGCCAGTTGTTATTTTCCCAAGTCCCTCTTTGTGGAGAG
AS_Tasmanian_Ssa06_sdY      GCCCTGTTCTAGGCCAGTTGTTATTTTCCCAAGTCCCTCTTTGTGGAGAG
Chinook_sdY                 ATATAAATATATTTGATTTGAAATTT-----------------------G
RT_sdY                      ATATAAATACATTTGATTTGAAATTT-----------------------G
                                   *  *    * ***  ****                       *
```

K. P. Lubieniecki *et al.*

```
BT_sdY                      ATTGACATATTATTAATATTAGGTGACCCCTTTGTTATTGGTTCAATTAC
AS_Tasmanian_Ssa02_sdY      ATTGACATATTATTAT-------------ATTTGTTTTTGGTTCAATTAC
AS_Tasmanian_Ssa03_sdY      ATTGACATATTATTAT-------------ATTTGTTTTTGGTTCAATTAC
AS_European_sdY             ATTGACATATTATTAT-------------ATTTGTTTTTGGTTCAATTAC
AS_Tasmanian_Ssa06_sdY      ATTGACATATTATTAT-------------ATTTGTTTTTGGTTCAATTAC
Chinook_sdY                 ATTAGCATATTATTATTATTCGATGAACACTTTGTTATTGGTTCATTTAC
RT_sdY                      ATTTGCATATTATTATTATTCGATGAACACTTTGTTGTTGGTTCATTTAC
                            ***  **********               ****** ******** ****

BT_sdY                      GGATAATAGCTTAAAGTCCATTTTTCACTAAGGGCTCGATTCAATTTGT-
AS_Tasmanian_Ssa02_sdY      GGATAATAGCTTAAAGTCCATTTTTCACTAAGGGCTCGATTCAATTTGT-
AS_Tasmanian_Ssa03_sdY      GGATAATAGCTTAAAGTCCATTTTTCACTAAGGGCTCGATTCAATTTGT-
AS_European_sdY             GGATAATAGCTTAAAGTCCATTTTTCACTAAGGGCTCGATTCAATTTGT-
AS_Tasmanian_Ssa06_sdY      GGATAATAGCTTAAAGTCCATTTTTCACTAAGGGCTCGATTCAATTTGT-
Chinook_sdY                 AGATAATTGCTTAAATTCAATGTTTCACTAAGGGC-CGATTCAATCTGTA
RT_sdY                      AGATAATTGCTTGAATTCAATGTTTCACTAAGGGCTCGATTCAATCTGTA
                             ****** **** ** ** ** ************* ********* ***

BT_sdY                      -------AGTTCGGGGCTAATGCTGAATTACATTTAAAGGTAA-ATTCCG
AS_Tasmanian_Ssa02_sdY      -------AGTTCGGGGCTAATGCTGAATTACATTTAAAGGTAA-ATTCTG
AS_Tasmanian_Ssa03_sdY      -------AGTTCGGGGCTAATGCTGAATTACATTTAAAGGTAA-ATTCTG
AS_European_sdY             -------AGTTCGGGGCTAATGCTGAATTACATTTAAAGGTAA-ATTCTG
AS_Tasmanian_Ssa06_sdY      -------AGTTCGGGGCTAATGCTGAATTACATTTAAAGGTAA-ATTCTG
Chinook_sdY                 TTGCGGAAGTTGAAGGCTAATGCTTGATTACATTTGAAGGTAA-TTTCCG
RT_sdY                      TTGCGGAAGTTCAAGGCTAATGCTTGATTACATTTGAAGGTAATTTTCCG
                                   ****   **********  ********* *******  *** *
```

```
BT_sdY                      ATTTAGCTGACATACTGTTTGCAGCATATACTGTAAATGCA------ACA
AS_Tasmanian_Ssa02_sdY      ATTTAGCTGACATACTGTTTGCAGCGTATACTGTAAATGCA------ACA
AS_Tasmanian_Ssa03_sdY      ATTTAGCTGACATACTGTTTGCAGCGTATACTGTAAATGCA------ACA
AS_European_sdY             ATTTAGCTGACATACTGTTTGCAGCGTATACTGTAAATGCA------ACA
AS_Tasmanian_Ssa06_sdY      ATTTAGCTGACATACTGTTTGCAGCGTATACTGTAAATGCA------ACA
Chinook_sdY                 ATTGAGCCGACATACTGTTTGCAGCATATAATGTAAATGCAGTCTCCACA
RT_sdY                      ATTGAGCCGACATACTGTTTGCAGCATATACTGCAAATGCAGTCTCCACA
                            *** *** ***************** **** ** *******      ***

BT_sdY                      AAATGCAGGATCATTGCCTTAAATGTATATTGCGCATTAGAACTGCTTTG
AS_Tasmanian_Ssa02_sdY      AAATGCAGGATCATTGCC------------------TTAGCACTGTTTTG
AS_Tasmanian_Ssa03_sdY      AAATGCAGGATCATTGCC------------------TTAGCACTGTTTTG
AS_European_sdY             AAATGCAGGATCATTGCC------------------TTAGCACTGTTTTG
AS_Tasmanian_Ssa06_sdY      AAATGCAGGATCATTGCC------------------TTAGCACTGTTTTG
Chinook_sdY                 AAATGCAGGAGCATTTCCTTAAATGTATTTTTGTCATTAGCACTGCATTG
RT_sdY                      AAATGCAGGAGCATTTCCTTAAATGTATTTTTGTCATTAGCACTGCATTG
                            ********** **** **                  **** ****  ***

BT_sdY                      ATGTTAATGTGAGCATCCCTCTCTTGTCCTACTGAATCTGGCTGATAATA
AS_Tasmanian_Ssa02_sdY      ATGTTAATGTGAGCATCCCTCTCTTGTCCTACTGAATCAGGCTGATAATA
AS_Tasmanian_Ssa03_sdY      ATGTTAATGTGAGCATCCCTCTCTTGTCCTACTGAATCTGGCTGATAATA
AS_European_sdY             ATGTTAATGTGAGCATCCCTCTCTTGTCCTACTGAATCTGGCTGATAATA
AS_Tasmanian_Ssa06_sdY      ATGTTAATGTGAGCATCCCTCTCTTGTCCTACTGAATCTGGCTGATAATA
Chinook_sdY                 ATGTTATTGTGAGCATCACTCTCTTGTCCTACTGAATTTGTCTGATTATA
RT_sdY                      ATGTTAATGTGAGCATCACTCTCTCGTACTACTGAATTTGTCTGATTATA
                            ****** ********** ****** ** *********  * ***** ***
```

```
BT_sdY                    TTACTGATTGTATCACTGATGAAATTACAGTAGTGTGTTTATCATGTGAT
AS_Tasmanian_Ssa02_sdY    TTACTGATTGTATCACTGATGAAATTACAGTAGTGTGTTTATCATGTGAT
AS_Tasmanian_Ssa03_sdY    TTACTGATTGTATCACTGATGAAATTACAGTAGTGTGTTTATCATGTGAT
AS_European_sdY           TTACTGATTGTATCACTGATGAAATTACAGTAGTGTGTTTATCATGTGAT
AS_Tasmanian_Ssa06_sdY    TTACTGATTGTATCACTGATGAAATTACAGTAGTGTGTTTATCATGTGAT
Chinook_sdY               TTACTGATTGTATCACTGATGATATTGCAGCAGTGTGTATGTCATGTGAT
RT_sdY                    TTACTGATTGTATCACTGATGATATTGCAGCGGTGTGTTTGTCATGTGAT
                          ********************** *** ***  ****** * *********

                                  |          exon3
                                                    |---------Exon 3F--------
BT_sdY                    GGATTACTGACTTTGTGTGTCCTTAG|AGTTGGAACGCTTCAGCCGAGCAG
AS_Tasmanian_Ssa02_sdY    GGATTACTGACTTTGTGTGTCCTTAG|AGTTGGAACGCTTCAGCAGAGCAG
AS_Tasmanian_Ssa03_sdY    GGATTACTGACTTTGTGTGTCCTTAG|AGTTGGAACGCTTCAGCAGAGCAG
AS_European_sdY           GGATTACTGACTTTGTGTGTCCTTAG|AGTTGGAACGCTTCAGCAGAGCAG
AS_Tasmanian_Ssa06_sdY    GGATTACTGACTTTGTGTGTCCTTAG|AGTTGGAACGCTTCAGCAGAGCAG
Chinook_sdY               GTATTACTGACTCTCTGTGTCCATAG|AGTTGGAACGCTTCGCCAGAGCAG
RT_sdY                    GTATTACTGACTCTGTGTGTCCTTAG|AGTTGGACCGCTTCGCCAGAGCAG
                          * ********** * ******* ***|******* ******  * ******

                             exon3
                          --->                                      <-------
BT_sdY                    ATGGCTTCCAACCGCAAATTGGGTTCAGCCTATGGTTCGGACAAGACTCA
AS_Tasmanian_Ssa02_sdY    ATGGCTTCCAACCGCAAATTGGGTTCAGCCTATGGTTCGGACAAGACTCA
AS_Tasmanian_Ssa03_sdY    ATGGCTTCCAACCGCAAATTGGGTTCAGCCTATGGTTCGGACAAGACTCA
AS_European_sdY           ATGGCTTCCAACCGCAAATTGGGTTCAGCCTATGGTTCGGACAAGACTCA
AS_Tasmanian_Ssa06_sdY    ATGGCTTCCAACCGCAAATTGGGTTCAGCCTATGGTTCGGACAAGACTCA
Chinook_sdY               ATGGCTCCCAACCTCAATGTGGGTTCAGCCTATGGTTTGGACAAGACTCA
RT_sdY                    ATGGCTCCCAACCTCAATGTGGGTTCAGCCTATGGTTTGGACAAGACTCA
                          ****** ****** ***  ***************** ************
```

```
                          exon3          |
                            ---Exon 3R---------          |
BT_sdY                      TCACTCAGTGCACCAATCTCTATATCG|GTACAGATTATTATTATTATATA
AS_Tasmanian_Ssa02_sdY      TCACTCAGTGCACCAATCTCTATATCG|GTACAGATTATTATTATTATATA
AS_Tasmanian_Ssa03_sdY      TCACTCAGTGCACCAATCTCTATATCG|GTACAGATTATTATTATTATATA
AS_European_sdY             TCACTCAGTGCACCAATCTCTATATCG|GTACAGATTATTATTATTATATA
AS_Tasmanian_Ssa06_sdY      TCACTCAGTGCACCAATCTCTATATCG|GTACAGATTATTATTATTATATA
Chinook_sdY                 TCACTCAGTGCACCAAACTTTATATCG|GTACAGATT----CTCTTAC---
RT_sdY                      TCACTCAGTGCACCAATCTTTATATCG|GTACAGATT----CTCTTAC---
                            **************** ** *******|*********     * ***

BT_sdY                      TAGATTATCTGGTGCCTTGAATACATTAACACAGCGCCGTCCTCAGAAAT
AS_Tasmanian_Ssa02_sdY      TAGATTATCTGGTGCCTTGAATACATTAACACAGTGCCGGCCTCAGAAAT
AS_Tasmanian_Ssa03_sdY      TAGATTATCTGGTGCCTTGAATACATTAACACAGTGCCGGCCTCAGAAAT
AS_European_sdY             TAGATTATCTGGTGCCTTGAATACATTAACACAGTGCCGGCCTCAGAAAT
AS_Tasmanian_Ssa06_sdY      TAGATTATCTGGTGCCTTGAATACATTAACACAGTGCCGGCCTCAGAAAT
Chinook_sdY                 -------TCTGGGGCATTGTACACATTAACACAGGGCCGACGCTAGAAGG
RT_sdY                      -------TCTGGGGCATTGTACACATTAACACAGGGCCGACGCTAGAAGG
                                   ***** ** *** * ************ **** *   ****

                                           <---AS&BT 10bo gap R--
BT_sdY                      AACCATTGGGAAGGGCAATTGATTTCCATAGGCGGACATGTTTTTTTTCA
AS_Tasmanian_Ssa02_sdY      AACCA--AGGAAGGGCAATTGATTTCCATAGGCGGACATGTTTTTTTTCA
AS_Tasmanian_Ssa03_sdY      AACCATTGGGAAGGGCAATTGATTTCCATAGGCGGACATGTTTTTTTTCA
AS_European_sdY             AACCATTGGGAAGGGCAATTGATTTCCATAGGCGGACATGTTTTTTTTCA
AS_Tasmanian_Ssa06_sdY      AACCA--AGGAAGGGCAATTGATTTCCATAGGCGGACATGTTTTTTTTCA
Chinook_sdY                 AATCATTGAGAAGGGCAATTGATTTCCATAGG-----ATG-TTTTTTTCA
RT_sdY                      AATCATTGAGAAG-------GATTTCCATAGG-----ATG-TTTTTTTCA
                            ** **    ****       ************     *** *********
```

K. P. Lubieniecki *et al.*

```
BT_sdY                     TGGCTTTTATAGTAAAGACATGTCATTTATCTTAACAAATGTATTACCTT
AS_Tasmanian_Ssa02_sdY     TGGCTTTTATAGTAAAGACATGTCATTTATCTTAACAAATGTATTACCTT
AS_Tasmanian_Ssa03_sdY     TGGCTTTTATAGTAAAGACATGTCATTTATCTTAACAAATGTATTACCTT
AS_European_sdY            TGGCTTTTATAGTAAAGACATGTCATTTATCTTAACAAATGTATTACCTT
AS_Tasmanian_Ssa06_sdY     TGGGTTTTATAGTAAAGACATGTCATTTATCTTAACAAATGTATTACCTT
Chinook_sdY                TGGTTTTTATAGTAAAG--ATGTAATTTATCTTTAAAAATATATTTCCTT
RT_sdY                     TGGTTTTTATAGTAAAG--ATGTAATTTTTCTTTAAAAATATATTCCCTT
                           *** *************  **** **** **** * **** **** ****

                                   -Exon 3-intron-end F2->
BT_sdY                     TTAATGTGGTATGAACACAACCAGTCATGATGTTTTCATCTGATTGTCAA
AS_Tasmanian_Ssa02_sdY     TTAATGTGGTATGAACACAACCAGTCATGATGTTTTCATCTGATTGTCAA
AS_Tasmanian_Ssa03_sdY     TTAATGTGGTATGAACACAACCAGTCATGATGTTTTCATCTGATTGTCAA
AS_European_sdY            TTAATGTGGTATGAACACAACCAGTCATGATGTTTTCATCTGATTGTCAA
AS_Tasmanian_Ssa06_sdY     TTAATGTGGTATGAACACAACCAGTCATGATGTTTTCATCTGATTGTCAA
Chinook_sdY                TTAATGTGGCATGTACACAACCAGTCATGATGTTTTCATCTGATTGTCAA
RT_sdY                     TTAATGTGGCACGTACACAACCAGTCATGATGTTTTCATCTGATTGTCAA
                           ********* * * ************************************

BT_sdY                     AC------------------------------------------------
AS_Tasmanian_Ssa02_sdY     ACGAATCTTTGTAAGTCGCTCTGGATAAGAGCGTCTGCTAAATGACTTAA
AS_Tasmanian_Ssa03_sdY     ACGAATCTTTGTAAGTCGCTCTGGATAAGAGCGTCTGCTAAATGACTTAA
AS_European_sdY            ACGAATCTTTGTAAGTCGCTCTGGATAAGAGCGTCTGCTAAATGACTTAA
AS_Tasmanian_Ssa06_sdY     ACGAATCTTTGTAAGTCGCTCTGGATAAGAGCGTCTGCTAAATGACTTAA
Chinook_sdY                AA------------------------------------------------
RT_sdY                     AA------------------------------------------------
                           *
```

```
BT_sdY                      ---------------GAATCACTTCAAAAAGTAGGTTACCTTTGCACGTT
AS_Tasmanian_Ssa02_sdY      ATGTTAAATGTAAATGAATCACTTCAAAAAGTAGGTTACCTTCGCACGTT
AS_Tasmanian_Ssa03_sdY      ATGTTAAATGTAAATGAATCACTTCAAAAAGTAGGTTACCTTCGCACGTT
AS_European_sdY             ATGTTAAATGTAAATGAATCACTTCAAAAAGTAGGTTACCTTCGCACGTT
AS_Tasmanian_Ssa06_sdY      ATGTTAAATGTAAATGAATCACTTCAAAAAGTAGGTTACCTTCGCACGTT
Chinook_sdY                 ---------------AAATAATTTCAAAAAGTATGTTACCTTCGCATGTT
RT_sdY                      ---------------AAATAATTTCAAAAAGTAGGTTACCTTCGCATGTT
                                            *** * *********** ******** *** ***

BT_sdY                      CGTCCAAAATAATTCTAGCATCCGAAGACTGCACTGTGTACCCGCCAACT
AS_Tasmanian_Ssa02_sdY      CGTCCAAAATAATTCTAGCATCCGAAGACTGCACTGTGTACCCGCCAACT
AS_Tasmanian_Ssa03_sdY      CGTCCAAAATAATTCTAGCATCCGAAGACTGCACTGTGTACCCGCCAACT
AS_European_sdY             CGTCCAAAATAATTCTAGCATCCGAAGACTGCACTGTGTACCCGCCAACT
AS_Tasmanian_Ssa06_sdY      CGTCCAAAATAATTCTAGCATCCGAAGACTGCACTGTGTACCCGCCAACT
Chinook_sdY                 CATCCAAAATAATTCTAGCATTTGAACTGGGCACTGTGGACCTGCCAATT
RT_sdY                      CATCCAAAATAATTCTAGCATTTGAAC-TGGCACTGTGGACCTGCCAATT
                            * *******************  ***    ******** *** ***** *

BT_sdY                      TTTGTTCAATTTGCAAGTGGT--------------TGGAGAAAACATCAG
AS_Tasmanian_Ssa02_sdY      TTTGTTCAATTTGCAAGTGGT--------------TGGAGAAAACATCTG
AS_Tasmanian_Ssa03_sdY      TTTGTTCAATTTGCAAGTGGT--------------TGGAGAAAACATCAG
AS_European_sdY             TTTGTTCAATTTGCAAGTGGT--------------TGGAGAAAACATCAG
AS_Tasmanian_Ssa06_sdY      TTTGTTCAATTTGCAAGTGGT--------------TGGAGAAAACATCTG
Chinook_sdY                 TTCTTTCAATTTGCACGTGGTTGAAACTATCTTACTGGAGAAAACATCAG
RT_sdY                      TTCTTTCAATTTGCAAGTGGCTGAAACTATCTTACTGGAGAAAACATCAG
                            **  *********** ****               ************* *
```

```
BT_sdY                      AGTGAGCAAAACAGCGTCTTG-----------------------------
AS_Tasmanian_Ssa02_sdY      AGCGAGCAAAAGAGCGTCTTG-----------------------------
AS_Tasmanian_Ssa03_sdY      AGCGAGCAAAAGAGCGTCTTG-----------------------------
AS_European_sdY             AGCGAGCAAAAGAGCGTCTTG-----------------------------
AS_Tasmanian_Ssa06_sdY      AGCGAGCAAAAGAGCGTCTTG-----------------------------
Chinook_sdY                 ATAGAGCAAAACAGTGCCTTACTATATGTAGCCCATGTTTCTGATGCTAT
RT_sdY                      ATAGAGCAAAACAGTGCCTTACTATATGTAGCCCATGTTTCTGATGCTAT
                            *  ******** ** * ***

BT_sdY                      ---------------------------GCTATTGTGGGCATGATAGCATC
AS_Tasmanian_Ssa02_sdY      ---------------------------GCTATTGTGGGCTAGACAGCATC
AS_Tasmanian_Ssa03_sdY      ---------------------------GCTATTGTGGGCTAGACAGCATC
AS_European_sdY             ---------------------------GCTATTGTGGGCTAGACAGCATC
AS_Tasmanian_Ssa06_sdY      ---------------------------GCTATTGTGGGCTAGACAGCATC
Chinook_sdY                 TTGGCTAAAAATAGTATGACATGTCATACTATTGTGGGCCAGACAGCATC
RT_sdY                      CTGGCTAAAAATAGTATGACATGTCATACTATTGTGGGCCAGACAGCATC
                                                        ***********  ** ******

BT_sdY                      AGA--------CTAGATACAGCAAGA--------CGCTGTTTCGCTCACT
AS_Tasmanian_Ssa02_sdY      AGG--------CTACGTATAGTAAGA--------CGCTGTTTCGCTCACT
AS_Tasmanian_Ssa03_sdY      AGG--------CTACATATAGTAAGA--------CGCTGTTTCGCTCACC
AS_European_sdY             AGG--------CTACATATAGTAAGA--------CGCTGTTTCGCTCACT
AS_Tasmanian_Ssa06_sdY      AGG--------CTACATATAGTAAGA--------CGCTGTTTCGCTCACT
Chinook_sdY                 AGACACATGGCCTACACACTCTGAGACAGAGGGGCGCTGTCTGGCTCACT
RT_sdY                      AGACACATGGCCTACACACTCTGAGACAGAGGGGCGCTGTCTGGCTCACT
                            **         ***   *     ***        ****** * ******
```

```
BT_sdY                      CGAATACTTTATCTGAGATTTACGTGCTTTTCTGTCGGCACGAGTCTCG-
AS_Tasmanian_Ssa02_sdY      CGAATACTTTATCTGAGATTTACGTGGTTTTCTGTCGGCACGAGTCTCGG
AS_Tasmanian_Ssa03_sdY      CGAATACTTTATCTGAGATTTACGTGGTTTTCTGTCGGCACGAGTCTCGG
AS_European_sdY             CGAATACTTTATCTGAGATTTACGTGGTTTTCTGTCGGCACGAGTCTCGG
AS_Tasmanian_Ssa06_sdY      CGAATACTTTATCTGAGATTTACGTGGTTTTCTGTCGGCACGAGTCTCGG
Chinook_sdY                 CAGATGCTTTATCTGAGATTGATGTGTCTTTCT----------GTCTCGG
RT_sdY                      CGGAAGCTTTATCTGAGATTGATGTGTCTTTCT----------GTCTCGG
                            *  *  ************** * ***  *****          ******

BT_sdY                      --------------------------------------------------
AS_Tasmanian_Ssa02_sdY      TCAAATTTAATTTGGAAGGGTAAGGACGTATGGTGGG------------G
AS_Tasmanian_Ssa03_sdY      TCAAATTTAATTTGGAAGGGTAAGGACGTATGGTGGG------------G
AS_European_sdY             TCAAATTTAATTTGGAAGGGTAAGGACGTATGGTGGG------------G
AS_Tasmanian_Ssa06_sdY      TCAAATTTAATTTGGAAGGGTAAGGACGTATGGTGGG------------G
Chinook_sdY                 TCAAATTTAATTTGGA--GGTACG---GTGGGGTGGGCCCGCCAAAACCA
RT_sdY                      TCAAATTTAATTTGGATGGGCAAG---GTGGGGTGGGCCCACCAAAACCA

BT_sdY                      --------------------------------------------------
AS_Tasmanian_Ssa02_sdY      CGGGCCCTGCATTAACTACAACAGTTATGATAACATTATGTTTTCCTCCA
AS_Tasmanian_Ssa03_sdY      CGGGCCCTGCATTAACTACAACAGTTATGATAACATTATGTTTTCCTCCA
AS_European_sdY             CGGGCCCTGCATTAACTACAACAGTTATGATAACATTATGTTTTCCTCCA
AS_Tasmanian_Ssa06_sdY      CGGGCCCTGCATTAACTACAACAGTTATGATAACATTATGTTTTCCTCCA
Chinook_sdY                 CAGGCCCTGCATTAACTTCAACAGTTATGATAACATTACATTTTCCTCTA
RT_sdY                      CAGGCCCTGCATTAACTTCAACAGTTATGATAACATTACATTTTCCTCTA
```

```
BT_sdY                      --------------------------------------------------
AS_Tasmanian_Ssa02_sdY      TTTTCCTGTAATTTTC----------------------------------
AS_Tasmanian_Ssa03_sdY      TTTTCCTGTAATTTTC----------------------------------
AS_European_sdY             TTTTCCTGTAATTTTC----------------------------------
AS_Tasmanian_Ssa06_sdY      TTTTCCTGTAATTTTC----------------------------------
Chinook_sdY                 TATTACTGTAATTTTCCACCATACAGTGCATTCGGAAAATATTCAGACCC
RT_sdY                      TATTACTGTAATTTTCCACCATACAGTGCATTCGGAAAGTATTCAAACCC

BT_sdY                      --------------------------------------------------
AS_Tasmanian_Ssa02_sdY      -------------------------GTTACAGCCTCATTCTAAAATTGAT
AS_Tasmanian_Ssa03_sdY      -------------------------GTTACAGCCTTATTCTAAAATTGAT
AS_European_sdY             -------------------------GTTACAGCCTTATTCTAAAATTGAT
AS_Tasmanian_Ssa06_sdY      -------------------------GTTACAGCCTTATTCTAAAATTGAT
Chinook_sdY                 ATTCCCTTTTCCCACATTTTGTTATGCTACAGCCTTATTCTTATATTGAT
RT_sdY                      ATTCCCTTTTCCCACATTTTGTTATGCTACAGCCTTATTCTAATATTGAT

BT_sdY                      --------------------------------------------------
AS_Tasmanian_Ssa02_sdY      TAAATAAATGTTTTTCCTCATCAATCTACACACAATACCTTATAATGGTA
AS_Tasmanian_Ssa03_sdY      TAAATAAATGTTTTTCCTCATCAATCTACACACAATACCTTATAATGGTA
AS_European_sdY             TAAATAAATGTTTTTCCTCATCAATCTACACACAATACCTTATAATGGTA
AS_Tasmanian_Ssa06_sdY      TAAATAAATGTTTTTCCTCATCAATCTACACACAATACCTTATAATGGTA
Chinook_sdY                 TAAAT---CGGTTTTCCTCATCAATCTACACACAATACCCCATAATGACA
RT_sdY                      TAAAT---CGTTTTTCCTCATCAATCTACACACAATACCCCATAATGACA
```

```
BT_sdY                    --------------------------------------------------
AS_Tasmanian_Ssa02_sdY    TATATAACAGATTTAAAGATTTTTTACAAATGT-------ATAAAAAAAT
AS_Tasmanian_Ssa03_sdY    TATATAACAGATTTAAAGATTTTTTACAAATGT-------ATAAAAAAAT
AS_European_sdY           TATATAACAGATTTAAAGATTTTTTACAAATGT-------ATAAAAAAAT
AS_Tasmanian_Ssa06_sdY    TATATAACAGATTTAAAGATTTTTTACAAATGT-------ATAAAAAAAT
Chinook_sdY               --------AGGTGAAAACAGGTTTTAAGAAAGTTTTGCTGATTAAAAAAC
RT_sdY                    --------AGGTGAAAACAGGTTTTAAGAAAGTTTTGCTGATTAAAAAAC


BT_sdY                    --------------------------------------------------
AS_Tasmanian_Ssa02_sdY    GT----------------TTATTTACATAAGTATTTAGACCCTTTGCTAT
AS_Tasmanian_Ssa03_sdY    GT----------------TTATTTACATAAGTATTTAGACCCTTTGCTAT
AS_European_sdY           GT----------------TTATTTACATAAGTATTTAGACCCTTTGCTAT
AS_Tasmanian_Ssa06_sdY    GT----------------TTATTTACATAAGTATTTAGACCCTTTGCTAT
Chinook_sdY               ATCAACAACCTATATATCTTATTTACATAAGATTTCAGACCCTTTGCTAT
RT_sdY                    ATCAACAACCTATACATCTTATTTACATAAGATTTCAGACCCTTTGCTAT


BT_sdY                    --------------------------------------------------
AS_Tasmanian_Ssa02_sdY    GAAACTCGAAATTAAGCTCAGCTGCATCCGGTTTC--CATTGATAATATT
AS_Tasmanian_Ssa03_sdY    GAAACTCGAAATTAAGCTCAGCTGCATCCGGTTTC--CATTGATAATATT
AS_European_sdY           GAAACTCGAAATTAAGCTCAGCTGCATCCGGTTTC--CATTGATAATATT
AS_Tasmanian_Ssa06_sdY    GAAACTCGAAATTAAGCTCAGCTGCATCCGGTTTC--CATTGATAATATT
Chinook_sdY               GAGAATCAAAATTAATATCAGCAGAATCCGGTTTCTGCTTTGATAATATT
RT_sdY                    GAGAATCAAAATTAATCTCAGCAGAATCCGGTTTC--CATTGATAATATT
```

```
                                                            ---Exon 3-4
BT_sdY                    ---------------------------------------------------
AS_Tasmanian_Ssa02_sdY    TGAGATGTTTCTACAACTTGATTGGAGTCCACCTCTGGTAAATTCAATTT
AS_Tasmanian_Ssa03_sdY    TGAGATGTTTCTACAACTTGATTGGAGTCCACCTCTGGTAAATTCAATTT
AS_European_sdY           TGAGATGTTTCTACAACTTGATTGGAGTCCACCTCTGGTAAATTCAATTT
AS_Tasmanian_Ssa06_sdY    TGAGATGTTTCTACAACTTGATTGGAGTCCACCTCTGGTAAATTCAATTT
Chinook_sdY               TTAGATGTTTCTACAACTTGAGTGGAGTCCACCTGTGGTGGATTCAATTC
RT_sdY                    TTAGATGTTTCTACAACTTGAGTGGAGTCCACCTGTGGTAGATTAAATTC


                          F2a------->
BT_sdY                    ---------------------------------------------------
AS_Tasmanian_Ssa02_sdY    A-TGGGACATGATTTGGAAAGGCACACTCCTGTCTATATAAGGTCCCACA
AS_Tasmanian_Ssa03_sdY    A-TGGGACATGATTTGGGAAGGCACACTCCTGTCTATATAAGGTCCCACA
AS_European_sdY           A-TGGGACATGATTTGGAAAGGCACACTCCTGTCTATATAAGGTCCCACA
AS_Tasmanian_Ssa06_sdY    A-TGGGACATGATTTGGAAAGGCACACTCCTGTCTATATAAGGTCCCACA
Chinook_sdY               ATTGGGGCATGATATTGAAAGGCACACACCTGTCTATACAAGGTCCCACA
RT_sdY                    ATTGGGGCATGATATTGAAAGGCACACACCTGTCTATACAAGGTCCCACA


BT_sdY                    ---------------------------------------------------
AS_Tasmanian_Ssa02_sdY    GTTGACAGTGCATTTCAGAGCAAAAACCAAGTCATGAGGGCAAAGGAATT
AS_Tasmanian_Ssa03_sdY    GTTGACAGTGCATTTCAGAGCAAAAACCAAGTCATGAGGGCAAAGGAATT
AS_European_sdY           GTTGACAGTGCATTTCAGAGCAAAAACCAAGTCATGAGGGCAAAGGAATT
AS_Tasmanian_Ssa06_sdY    GTTGACAGTGCATTTCAGAGCAAAAACCAAGTCATGAGGGCAAAGGAATT
Chinook_sdY               GTTGACAGTGCATGTCAGGGCAAAAACAAAGCCATGAGATTGAAAGAATT
RT_sdY                    GTTGACAGTGCATGTCAGGGCAAAAACCAAGCCATGAGATTGAAAGAATT
```

```
BT_sdY                      --------------------------------------------------
AS_Tasmanian_Ssa02_sdY      ATCCGTAGGGCTCCAAAACAGGATTGTGTATAGGCACAGATCAGGGGAAG
AS_Tasmanian_Ssa03_sdY      ATCCGTAGAGCTCCAAAACAGGATTGTGTATAGGCACAGATCAGGGGAAG
AS_European_sdY             ATCCGTAGAGCTCCAAAACAGGATTGTGTATAGGCACAGATCAGGGGAAG
AS_Tasmanian_Ssa06_sdY      ATCCGTAGAGCTCCAAAACAGGATTGTGTATAGGCACAGATCAGGGGAAG
Chinook_sdY                 ATCCG----------AAACAAGATTGTGTTGAGGCACAGATC-TGGGAAG
RT_sdY                      ATCCG----------AAACAAGATTGTGTTGAGGCACAGATC-TGGGAAG


BT_sdY                      --------------------------------------------------
AS_Tasmanian_Ssa02_sdY      GGTACCAAAACATTTCTGAAGCATTGAAGGTCCCCAAGAACACAGTGGTC
AS_Tasmanian_Ssa03_sdY      GGTACCAAAACATTTCTGAAGCATTGAAGGTCCCCAAGAACACAGTGGTC
AS_European_sdY             GGTACCAAAACATTTCTGAAGCATTGAAGGTCCCCAAGAACACAGTGGTC
AS_Tasmanian_Ssa06_sdY      GGTACCAAAACATTTCTGAAGCATTGAAGGTCCCCAAGAACACAGTGGTC
Chinook_sdY                 GGTACCAAAACATTTCTGAAGCATTGAAGGTCCCCAAGA--TCAGTGGTC
RT_sdY                      GGTACCAAAACGTTTCTGAA-----------CCCCAAGA--TCAATGGTC


BT_sdY                      --------------------------------------------------
AS_Tasmanian_Ssa02_sdY      TCCATCATTCTTAAATGGAAGAAGTCTGGAAACACCAAGATTCTTCCTAG
AS_Tasmanian_Ssa03_sdY      TCCATCATTCTTAAATGGAAGAAGTTTGGAAACACCAAGATTCTTCCTAG
AS_European_sdY             TCCATCATTCTTAAATGGAAGAAGTTTGGAAACACCAAGATTCTTCCTAG
AS_Tasmanian_Ssa06_sdY      TCCATCATTCTTAAATGGAAGAAGTTTGGAAACACCAAGATTCTTCCTAG
Chinook_sdY                 TCCATCATTCTGAAATGGAAGAAGTTTGGAACCACCGAAACTCATCCTAG
RT_sdY                      TCCATCATTCTGAAATGGAAGAAGTTTGGAACCACTGAAACTCATCCTAG
```

```
BT_sdY                    --------------------------------------------------
AS_Tasmanian_Ssa02_sdY    AGCTGGCCACCCG-------------------------------------
AS_Tasmanian_Ssa03_sdY    AGCTGGCCACCCG-------------------------------------
AS_European_sdY           AGCTGGCCACCCG-------------------------------------
AS_Tasmanian_Ssa06_sdY    AGCTGGCCACCCG-------------------------------------
Chinook_sdY               AGTTGGCCGCCCAGAGCTGGACTCCTGAGTGGAGCAGTGGTCTAAGGCAC
RT_sdY                    AGTTGGCCGCCCA------------------GAGCAGCGGTCTAAGGCAC

BT_sdY                    --------------------------------------------------
AS_Tasmanian_Ssa02_sdY    --------------------------------------------------
AS_Tasmanian_Ssa03_sdY    --------------------------------------------------
AS_European_sdY           --------------------------------------------------
AS_Tasmanian_Ssa06_sdY    --------------------------------------------------
Chinook_sdY               TGCATCTTAGTGCTAGATTGGTCACTACAGTCCCGGTTTCGATCCCGGGC
RT_sdY                    TGCATCTCAGTGCTAGATTGGTCACTACAGTCCCGGTTTCGATCCCGGGC

BT_sdY                    --------------------------------------------------
AS_Tasmanian_Ssa02_sdY    --------------------------------------------------
AS_Tasmanian_Ssa03_sdY    --------------------------------------------------
AS_European_sdY           --------------------------------------------------
AS_Tasmanian_Ssa06_sdY    --------------------------------------------------
Chinook_sdY               TGTATCACAACCGGTCGTGATTTGGAGTCCCATAGGGCGCCGTACAATTT
RT_sdY                    TGTATCACAACCGGTCGTGATTTGGAGTCCCATAGGGCGGCGTACAATTT
```

```
BT_sdY                      ----------------------------------------GCCAAACTGA
AS_Tasmanian_Ssa02_sdY      ----------------------------------------GCCAACCTGA
AS_Tasmanian_Ssa03_sdY      ----------------------------------------GCCAACCTGA
AS_European_sdY             ----------------------------------------GCCAACCTGA
AS_Tasmanian_Ssa06_sdY      ----------------------------------------GCCAACCTGA
Chinook_sdY                 GGCCCAGCGTCGTCCGGGTTAGGGGAGGCCGGGGGAGGCCGTCATTGTAA
RT_sdY                      GGCCCAGCGTTGTCCGGGTTAGGGGAGGCCGGGGGAGGCCGTCATTGTAA
                                                                    * **   * *

BT_sdY                      GCA-----------------------------------------------
AS_Tasmanian_Ssa02_sdY      GCA-----------------------------------------------
AS_Tasmanian_Ssa03_sdY      GCA-----------------------------------------------
AS_European_sdY             GCA-----------------------------------------------
AS_Tasmanian_Ssa06_sdY      GCA-----------------------------------------------
Chinook_sdY                 GCAAGAATTTGTTTTTATATGACTTGCCTAGTTAAATAATGGTTAA--AT
RT_sdY                      ACAAGAATTTGTTTTTATATTACTTGCCTAGTTAAATAATGGCTAAATAT
                             **

BT_sdY                      ----------------ATCGGGGGAGAAGGGCCTTGGTCATGGAGGTGAT
AS_Tasmanian_Ssa02_sdY      ----------------ATCGGGGGAGAAGGGCCTTGGTCAGGGAGGTGAT
AS_Tasmanian_Ssa03_sdY      ----------------ATCGGGGGAGAAGGGCCTTGGTCAGGGAGGTGAT
AS_European_sdY             ----------------ATCGGGGGAGAAGGGCCTTGGTCAGGGAGGTGAT
AS_Tasmanian_Ssa06_sdY      ----------------ATCGGGGGAGAAGGGCCTTGGTCAGGGAGGTGAT
Chinook_sdY                 ATATTTATTTTTTATTATCAGGGGAGAAGGGCCTTGGTCAGGGAGGTGAC
RT_sdY                      ATATTTATTTTTTATTATCAGGGGAGAAGGGCCTTGGTCAGGGAGGTGAC
                                            *** ******************** ********
```

```
BT_sdY                    CAAGAACCCGATTGTCAATCGGACAGAGCTCCAGAGTTCCTCTGTGGAGA
AS_Tasmanian_Ssa02_sdY    CAAGAACCCGATTGTCAATCGGACAGAGCTCCAGAGTTCCTCTGTGGAGA
AS_Tasmanian_Ssa03_sdY    CAAGAACCCGATTGTCAATCGGACAGAGCTCCAGAGTTCCTCTGTGGAGA
AS_European_sdY           CAAGAACCCGATTGTCAATCGGACAGAGCTCCAGAGTTCCTCTGTGGAGA
AS_Tasmanian_Ssa06_sdY    CAAGAACCCGATTGTCAATCGGACAGAGCTCCAGAGTTCCTCTGTGGAGA
Chinook_sdY               CAATAACCTGATGGTCACTCTGACATAGCTCCAAAGTTCCTCTGTGGAAA
RT_sdY                    CAATAACCTGATGGTC-CTCTGACATAGCTCCAAAGTTCCTCTGTGGAGA
                          *** **** *** ***  ** **** ******* ************** *

BT_sdY                    TGGGAGAACCTTCCAGAAGGACAACCATCTCTGCAGCACTCCACCAATCA
AS_Tasmanian_Ssa02_sdY    TGGGAGAACCTTCCAGAAGGACAACCATCTCTGCAGCACTCCACCAATCA
AS_Tasmanian_Ssa03_sdY    TGGGAGAACCTTCCAGAAGGACAACCATCTCTGCAGCACTCCACCAATCA
AS_European_sdY           TGGGAGAACCTTCCAGAAGGACAACCATCTCTGCAGCACTCCACCAATCA
AS_Tasmanian_Ssa06_sdY    TGGGAGAACCTTCCAGAAGGACAACCATCTCTGCAGCACTCCACCAATCA
Chinook_sdY               TGGGAGAATCTTCCAGAATGACAGCCATCTCTGCAACACTCCAATAATCA
RT_sdY                    TGGGAGAATCTTCCAGAATGACAGCCATCTCTGCAGCACTCCACTAATCA
                          ******** ********* **** *********** *******  *****

BT_sdY                    GGCCTTTATGGTAGAGTGTCCAGACGGAAGCCACTCCTCAGTAAAAGGCA
AS_Tasmanian_Ssa02_sdY    GGCCTTCATGGTAGAGTGTCCAGACGGGAGCCACTCCTCAGTAAAAGGCA
AS_Tasmanian_Ssa03_sdY    GGCCTTCATGGTAGAGTGTCCAGACGGGAGCCACTCCTCAGTAAAAGGCA
AS_European_sdY           GGCCTTCATGGTAGAGTGTCCAGACGGGAGCCACTCCTCAGTAAAAGGCA
AS_Tasmanian_Ssa06_sdY    GGCCTTCATGGTAGAGTGTCCAGACGGGAGCCACTCCTCAGTAAAAGGCA
Chinook_sdY               GGCCTTTATAGTAGAGTGGCCAGACGGAAGCCACTCCTCAGTAAAAGGCA
RT_sdY                    GGCCTTTTTGGTAGAGTGGCCAGACGGAAGCCACTCCTCAGTAAAAGGCA
                          ******  * ******** ******** **********************
```

```
BT_sdY                    CATGACAGCCCGCTTGTAGTTTGCCAAAAGGAACCTAAAGGACTCTCAGA
AS_Tasmanian_Ssa02_sdY    CATGACAGCCCTCTTGTAGTTTGCCAAAAGGAACCTAAAGGACTCTCAGA
AS_Tasmanian_Ssa03_sdY    CATGACAGCCCGCTTGTAGTTTGCCAAAAGGAATCTAAAGGACTCTCAGA
AS_European_sdY           CATGACAGCCCGCTTGTAGTTTGCCAAAAGGAACCTAAAGGACTCTCAGA
AS_Tasmanian_Ssa06_sdY    CATGACAGCCCTCTTGTAGTTTGCCAAAAGGAACCTAAAGGACTCTCAGA
Chinook_sdY               CATGACAGCCCACTTGGAGTTTGCCAAAAGGAACATAAAGCACTCTCAGA
RT_sdY                    CATGACAGCCCACTTGGAGTTTGCCAAAAGGAACATAAAGCACTCTCAGA
                          *********** **** ****************  ***** *********

BT_sdY                    CCATGAGAAACAAGATTCTCTTGTCTGAATAAACCAAGATTGAACACTTT
AS_Tasmanian_Ssa02_sdY    CCATGAGAAACAAGATTCTCTTGTCTGAATAAACCAAGATTGAACACTTT
AS_Tasmanian_Ssa03_sdY    CCATGAGAAACAAGATTCTCTTGTCTGAATAAACCAAGATTGAACACTTT
AS_European_sdY           CCATGAGAAACAAGATTCTCTTGTCTGAATAAACCAAGATTGAACACTTT
AS_Tasmanian_Ssa06_sdY    CCATGAGAAACAAGATTCTCTTGTCTGAATAAACCAAGATTGAACACTTT
Chinook_sdY               CCATGAGAAACAAGATTCTCTGCTCTGATTTAATCAAGATTTAACTCTAT
RT_sdY                    CCATGAGAAACAAGATTCTCTGCTCTGATTAAATCAAGATTTAACTCTAT
                          *********************  ***** * ** ******* *** ** *

BT_sdY                    GGCCT-GAATGCCAAGTGTCACATCTGGAGGAAAGCTGGCACCATCCCTA
AS_Tasmanian_Ssa02_sdY    GGCCT-GAATGCCAAGTGACACATCTGGAGGAAAGCTGGCACCATCCCTA
AS_Tasmanian_Ssa03_sdY    GGCCT-GAATGCCAAGTGTCACATCTGGAGGAAAGCTGGCACCATCCCTA
AS_European_sdY           GGCCT-GAATGCCAAGTGTCACATCTGGAGGAAAGCTGGCACCATCCCTA
AS_Tasmanian_Ssa06_sdY    GGCCT-GAATGCCAAGTGTCACATCTGGAGGAAAGCTGGCACCATCCCTA
Chinook_sdY               GGCCT-AAATTACAAGCGTCACATCTGGTGGAAAGCTGGCACCATCCCTG
RT_sdY                    GGCCTAAAATTACAAGCGTCACATCTGGTGGAAAGCTGGCACCATCCCTA
                          *****  ***  **** * ********* ********************
```

```
BT_sdY                    CAGTGAAGCATGGTGGGAGCAGCATCATGCTGTGGGGATGTTTTTCAGCG
AS_Tasmanian_Ssa02_sdY    CGGTGAAGCATGGTGGGGGCAGCATCATGCTGTGGGGATGTTTTTCAGCG
AS_Tasmanian_Ssa03_sdY    CAGTGAAGCATGGTGGGGGCAGCATCATGCTGCGGGGATGTTTTTCAGCG
AS_European_sdY           CAGTGAAGCATGGTGGGGGCAGCATCATGCTGTGGGGATGTTTTTCAGCG
AS_Tasmanian_Ssa06_sdY    CGGTGAAGCATGGTGGGGGCAGCATCATGCTGTGGGGATGTTTTTCAGCG
Chinook_sdY               TGA-----------------------------------------------
RT_sdY                    TGA-----------------------------------------------

BT_sdY                    GCAGGGACTGGGATCGAGGGAAAGATTAACAGAGCAAAGTACAGAGAGAT
AS_Tasmanian_Ssa02_sdY    GCAGGGACTGGGATCGAGGGAAAGATTAACAGAGCAAAGTACAGAGAGAT
AS_Tasmanian_Ssa03_sdY    GCAGGGACTGGGATCGAGGGAAAGATTAACAGAGCAAAGTACGGAGAGAT
AS_European_sdY           GCAGGGACTGGGATCGAGGGAAAGATTAACAGAGCAAAGTACAGAGAGAT
AS_Tasmanian_Ssa06_sdY    GCAGGGACTGGGATCGAGGGAAAGATTAACAGAGCAAAGTACAGAGAGAT
Chinook_sdY               --------------------------------------------------
RT_sdY                    --------------------------------------------------

BT_sdY                    TCTTGATCCAGAGCGCTCAGGACCTCAGACTGGGGCTAAGGTTCACCTTC
AS_Tasmanian_Ssa02_sdY    TCTTGATCCAGAGCGCTCAGGACCTCAGACTGGGGCTAAGGTTCACCTTC
AS_Tasmanian_Ssa03_sdY    TCTTGATCCAGAGCGCTCAGGACCTCAGACTGGGGCTAAGGTTTACCTTC
AS_European_sdY           TCTTGATCCAGAGCGCTCAGGACCTCAGACTGGGGCTAAGGTTCACCTTC
AS_Tasmanian_Ssa06_sdY    TCTTGATCCAGAGCGCTCAGGACCTCAGACTGGGGCTAAGGTTCACCTTC
Chinook_sdY               --------------------------------------------------
RT_sdY                    --------------------------------------------------
```

```
BT_sdY                    CAACAGGACAACGACCGTAAACACACAGCCAAGAAAACGCAGGAGTGGCT
AS_Tasmanian_Ssa02_sdY    CAACAGGACAACGACCGTAAACACACAGCCAAGACAACGCAGGAGTGGCT
AS_Tasmanian_Ssa03_sdY    CAACAGGACGACGACCGTAAACACACAGCCAAGACAACGCAGGAGTGGCT
AS_European_sdY           CAACAGGACAACGACCGTAAACACACAGCCAAGACAACGCAGGAGTGGCT
AS_Tasmanian_Ssa06_sdY    CAACAGGACAACGACCGTAAACACACAGCCAAGACAACGCAGGAGTGGCT
Chinook_sdY               --------------------------------------------------
RT_sdY                    --------------------------------------------------

BT_sdY                    TCGGGACAAGTCTCTGAATTTCATTCACGAGCACGGACTTGAACCCGATC
AS_Tasmanian_Ssa02_sdY    TCAGGACAAGTCTCTGAATTTCATTCATGAGCACGGACTTGAACCCGATC
AS_Tasmanian_Ssa03_sdY    TCAGGACAAGTCTCTGAATTTCATTCATGAGCACGGACTTGAACCCGATC
AS_European_sdY           TCAGGACAAGTCTCTGAATTTCATTCATGAGCACGGACTTGAACCCGATC
AS_Tasmanian_Ssa06_sdY    TCAGGACAAGTCTCTGAATTTCATTCATGAGCACGGACTTGAACCCGATC
Chinook_sdY               --------------------------------------------------
RT_sdY                    --------------------------------------------------

BT_sdY                    TAACATCTCTGTAGAGACCTGAAAATAGCTGTGCAGCGACATTTCCCATC
AS_Tasmanian_Ssa02_sdY    TAACATCTCTGTAGAGACCTGAAAATAGCTGTGCAGCGACATTTCCCATT
AS_Tasmanian_Ssa03_sdY    TAACATCTCTGTAGAGACCTGAAAATAGCTGTGCAGCGACATTTCCCATT
AS_European_sdY           TAACATCTCTGTAGAGACCTGAAAATAGCTGTGCAGCGACATTTCCCATT
AS_Tasmanian_Ssa06_sdY    TAACATCTCTGTAGAGACCTGAAAATAGCTGTGCAGCGACATTTCCCATT
Chinook_sdY               --------------------------------------------------
RT_sdY                    --------------------------------------------------
```

```
BT_sdY                   CAACCTGACAGAGCTTGAGAGGATCTGCAGAGAATAATGAGAGAAACTCC
AS_Tasmanian_Ssa02_sdY   CAACCTGACAGAGCTTGAGAGGATCTGCAGAGAAGAATGAGAGAAACTCC
AS_Tasmanian_Ssa03_sdY   CAACCTGACAGAGCTTGAGAGGATCTGCAGAGAAGAATGAGAGAAACTCC
AS_European_sdY          CAACCTGACAGAGCTTGAGAGGATCTGCAGAGAAGAATGAGAGAAACTCC
AS_Tasmanian_Ssa06_sdY   CAACCTGACAGAGCTTGAGAGGATCTGCAGAGAAGAATGAGAGAAACTCC
Chinook_sdY              --------------------------------------------------
RT_sdY                   --------------------------------------------------


BT_sdY                   CCAAATACAGGTGTGCCAAGCTTGTAGCGTCATACCCAAGAAGACTCGAT
AS_Tasmanian_Ssa02_sdY   CCAAATACAGGTGTGCCAAGCTTGTAGCGTCATACCCAAGAAGACTTGAT
AS_Tasmanian_Ssa03_sdY   CCAAATACAGGTGTGCCAAGCTTGTAGCGTCATACCCAAGAAGACTTGAT
AS_European_sdY          CCAAATACAGGTGTGCCAAGCTTGTAGCGTCATACCCAAGAAGACTTGAT
AS_Tasmanian_Ssa06_sdY   CCAAATACAGGTGTGCCAAGCTTGTAGCGTCATACCCAAGAAGACTTGAT
Chinook_sdY              --------------------------------------------------
RT_sdY                   --------------------------------------------------


BT_sdY                   GCTGTAATCGCTGCCAAAGGTGTTTCAACAAAGTACTGAGTAAAGGGTCT
AS_Tasmanian_Ssa02_sdY   GCTGTAATCGCTGCCAAAGGTGCTTCAACAAAGTACTGAGTAAAGGGTCT
AS_Tasmanian_Ssa03_sdY   GCTGTAATCGCTGCCAAAGGTGCTTCAACAAAGTACTGAGTAAAGGGTCT
AS_European_sdY          GCTGTAATCGCTGCCAAAGGTGCTTCAACAAAGTACTGAGTAAAGGGTCT
AS_Tasmanian_Ssa06_sdY   GCTGTAATCGCTGCCAAAGGTGCTTCAACAAAGTACTGAGTAAAGGGTCT
Chinook_sdY              -------------------------------------------------T
RT_sdY                   -------------------------------------------------T
                                                                          *
```

```
BT_sdY                      GAATACTTATCTAAATGTGATATTTC---------CGTTTTAA----AT-
AS_Tasmanian_Ssa02_sdY      GAATACTTATCTAAATGTGATATTTC---------CGTTTTAA----AT-
AS_Tasmanian_Ssa03_sdY      GAATACTTATCTAAATGTGATATTTC---------CGTTTAAA----AT-
AS_European_sdY             GAATACTTATCTAAATGTGATATTTC---------CGTTTAAA----AT-
AS_Tasmanian_Ssa06_sdY      GAATACTTATCTAAATGTGATATTTC---------CGTTTTAA----AT-
Chinook_sdY                 GAATACTCATGTAAATGTAATATTTCAATTTTTTTATTTCAATTTTTT-
RT_sdY                      GAATACTCATGTAAATGTAATATTTCAAAAACAAATATTTCAA----ATG
                            ******* ** ******* *******           *** **     *

BT_sdY                      TTGTAAT--GAATTGGCTAATATTTCTAAAAACCTGTTTTTGCTTTGTCA
AS_Tasmanian_Ssa02_sdY      TTGTAAT--GAATTGGCTAATATTTCTAAAAACCTGTTTTTGCTTTGTCA
AS_Tasmanian_Ssa03_sdY      TTGTAAT--GAATTGGCTAATATTTCTAAAAACCTGTTTTTGCTTTGTCA
AS_European_sdY             TTGTAAT--GAATTGGCTAATATTTCTAAAAACCTGTTTTTGCTTTGTCA
AS_Tasmanian_Ssa06_sdY      TTGTAAT--GAATTGGCTAATATTTCTAAAAACCTGTTTTTGCTTTGTCA
Chinook_sdY                 TTTAAAT--AAATTGGCTAATATTTCTACACACCTGTTTTTTCTTTGTCA
RT_sdY                      TTTTAATAGAAATTGGCTAATATTTCTACACACCTGTTTTTTCTTTGTCA
                            **  ***   ****************** * ********** ********

BT_sdY                      TTATGGGGTATTGTGTGTAGAATAATGA-GGGAAAAAACTATTTAATCCA
AS_Tasmanian_Ssa02_sdY      TTATGGGGTA-TGTGTGTAGATTAATGAGGGGAAAAAACTATTTAATCCA
AS_Tasmanian_Ssa03_sdY      TTATGGGGTA-TGTGTGTAGATTAATGAGGGGAAAAAACAATTTAATCCA
AS_European_sdY             TTATGGGGTA-TGTGTGTAGATTAATGAGGGGAAAAAACAATTTAATCCA
AS_Tasmanian_Ssa06_sdY      TTATGGGGTA-TGTGTGTAGATTAATGAGGGGAAAAAACTATTTAATCCA
Chinook_sdY                 TTGTGGGGTATTGTGTGTAGATTAATGA-GGGAAAAAGCAATTTAATCAA
RT_sdY                      TTGTGGGGTATTGTGTGTAGATTAATGAGGGGAAAAGA-AATTTAATCAA
                            ** ******* ********** ****** *******    ******** *
```

```
BT_sdY                  TTTTAGAATAAGGCTATAATGTAACAAAACGTGGAAAAAGTCAAGGGGTC
AS_Tasmanian_Ssa02_sdY  TTTTAGAATAAGGCTATAATGTAACAAAACGTGGAAAAAGTCGAGGTGTC
AS_Tasmanian_Ssa03_sdY  TTTTAGAATAAGGCTATAATGTAACAAAACGTGGAAAAAGTCAAGGTGTC
AS_European_sdY         TTTTAGAATAAGGCTATAATGTAACAAAACGTGGAAAAAGTCAAGGTGTC
AS_Tasmanian_Ssa06_sdY  TTTTAGAATAAGGCTATAATGTAACAAAACGTGGAAAAAGTCAAGGTGTC
Chinook_sdY             TTTTGGAATAATGCTGAAATGTAACACAATTTGGAAAATGTCAAGGAG--
RT_sdY                  TTTTGGAATAATGCTGAAATGTAACACAATTTGGAAAATGTCAAGGAGAC
                        **** ****** ***  ********* **  ******* *** *** *

BT_sdY                  TGAATCCTTTCCCAATGCACTGTATATGTGTGAATGTGTATGAGGCATTA
AS_Tasmanian_Ssa02_sdY  TGAATACTTTCCCAATGCACTGTATATGTGTGAATGTGTATGAGGCGTTA
AS_Tasmanian_Ssa03_sdY  TGAATACTTTCCCAATGCACTGTATATGTGTGAATGTGTATGAGGCGTTA
AS_European_sdY         TGAATACTTTCCCAATGCACTGTATATGTGTGAATGTGTATGAGGCGTTA
AS_Tasmanian_Ssa06_sdY  TGAATACTTTCCCAATGCACTGTATATGTGTGAATGTGTATGAGGCGTTA
Chinook_sdY             -----ACTTTCCCAATGC-----ATATGTGTAAATGTGTATGGGGCCTTA
RT_sdY                  TGAATACTTTCCCAATGC-----ATATGTGTAAACGTGTATGGGGCCTTA
                              ************     ******** ** ******* *** ***

BT_sdY                  CAGA-----GCCACTTGATGTTGATTATAATTAGATGTT-AACAATCCTT
AS_Tasmanian_Ssa02_sdY  CAGA-----GCCACTTGATGTTGATTATAATTAGATGTT-CACAATCCTT
AS_Tasmanian_Ssa03_sdY  CAGA-----GCCACTTGATGTTGATTATAATTAGATGTT-CACAATCCTT
AS_European_sdY         CAGA-----GCCACTTGATGTTGATTATAATTAGATGTT-CACAATCCTT
AS_Tasmanian_Ssa06_sdY  CAGA-----GCCACTTGATGTTGATTATAATTAGATGTT-CACAATCCTT
Chinook_sdY             CTTACTTACTCCACTTGGTGTTGATTAT---------------AATCTTT
RT_sdY                  CTTACTTACTCCACTTGGTGTTGATTATAATTAGATGTTGAACAATCTTT
                        *  *      ******* **********               **** **
```

```
                          |             exon4
BT_sdY                      CCTTCTCCAG|ATTAAATTGCCATGGGCTCAGCAGCTATTCAAGCAAGCTC
AS_Tasmanian_Ssa02_sdY      CCTTCTCCAG|ATTAAATTGCCATGGGCTCAGCAGCTATTCAAGCAAGCTC
AS_Tasmanian_Ssa03_sdY      CCTTCTCCAG|ATTAAATTGCCATGGGCTCAGCAGCTATTCAAGCAAGCTC
AS_European_sdY             CCTTCTCCAG|ATTAAATTGCCATGGGCTCAGCAGCTATTCAAGCAAGCTC
AS_Tasmanian_Ssa06_sdY      CCTTCTCCAG|ATTAAATTGCCATGGGCTCAGCAGCTATTCAAGCAAGCTC
Chinook_sdY                 CCTTCTCCAG|ATTAAATTGCCATGGGCTCAGCAGCTATTCAAGGAAGTTC
RT_sdY                      CCTTCTCCAG|ATTAAATTACCATGGGCCCAGCAGCTATTCAAGGAAGTTC
                            **********|******** ******** *************** *** **

                              exon4                      |
BT_sdY                      ACGACTTCAGGATCTGGCTTGAGTCCTCCCCTGTCTCTCCTGGAGTCTGA|
AS_Tasmanian_Ssa02_sdY      ACGACTTCAGGATCTGGCTTGAGTCCTCCCCTGTCTCTCCTGGAGTCTGA|
AS_Tasmanian_Ssa03_sdY      ACGACTTCAGGATCTGGCTTGAGTCCTCCCCTGTCTCTCCTGGAGTCTGA|
AS_European_sdY             ACGACTTCAGGATCTGGCTTGAGTCCTCCCCTGTCTCTCCTGGAGTCTGA|
AS_Tasmanian_Ssa06_sdY      ACGACTTCAGGATCTGGCTTGAGTCCTCCCCTGTCTCTCCTGGAGTCTGA|
Chinook_sdY                 ATGACTTCAGGATCTGGCTTGAGTCCTCCCCTGTCTCTCCTGGAGTCTGA|
RT_sdY                      ATGACTTCAGGATCTGGCTTGAGTCCTCCCCTGTCTCTCCTGGAGTCTGA|
                            * ************************************************|

BT_sdY                      AAATATGGTTG--GTCCCAACAGGACTCCAATTGCTCCCAATATTAGCCT
AS_Tasmanian_Ssa02_sdY      AAATATGGTTG--GTCCCAACAGGACTCCAATTGCTCCCAATATTAGCCT
AS_Tasmanian_Ssa03_sdY      AAATATGGTTG--GTCCCAACAGGACTCCAATTGCTCCCAATATTAGCCT
AS_European_sdY             AAATATGGTTG--GTCCCAACAGGACTCCAATTGCTCCCAATATTAGCCT
AS_Tasmanian_Ssa06_sdY      AAATATGGTTG--GTCCCAACAGGACTCCAATTGCTCCCAATATTAGCCT
Chinook_sdY                 AGAACCGGTTGGAGTCCCAACAGGA---------CTCCCAATATGAGCCT
RT_sdY                      AGAACTGGTTGGAGTCCCAACAGGACCCCAACTGCTCCCAATATGAGCCT
                            * *   *****  ************         ********** *****
```

```
BT_sdY                      ATGCCACAGCCCTTTCCACCACACCTGTTCCCACACCCTAATCACC-TCT
AS_Tasmanian_Ssa02_sdY      ATGCCACAGCCCTTTCCACCACACCTGTTCCCACACCCTAATCACC-TCT
AS_Tasmanian_Ssa03_sdY      ATGCCACAGCCCTTTCCACCACACCTGTTCCCACACCCTAATCACC-TCT
AS_European_sdY             ATGCCACAGCCCTTTCCACCACACCTGTTCCCACACCCTAATCACC-TCT
AS_Tasmanian_Ssa06_sdY      ATGCCACAGCCCTTTCCACCACACCTGTTCCCACACCCTAATCACC-TCT
Chinook_sdY                 AGGCCACAGCCTTTCCCACCAAACTTGT------ACCCTAATCACC-TCT
RT_sdY                      AGGCCACAGCCTTTCCCACCAAACCTCT------ACCCTAATCACCTTCT
                            * ********* ** ****** ** * *      ************ ***

BT_sdY                      CCTCCACCTTTTTACCTCTTTTCATTCCCTATGTAAGCAGCATGCTTCTT
AS_Tasmanian_Ssa02_sdY      CCTCCACCTTTTTACCTCTTTTCATTCCCTATATAAGCAGTATGCTTCTT
AS_Tasmanian_Ssa03_sdY      CCTCCACCTTTTTACCTCTTTTCATTCCCTATATAAGCAGTATGCTTCTT
AS_European_sdY             CCTCCACCTTTTTACCTCTTTTCATTCCCTATATAAGCAGTATGCTTCTT
AS_Tasmanian_Ssa06_sdY      CCTCCACCTTTTTACCTCTTTTCATTCCCTATATAAGCAGTATGCTTCTT
Chinook_sdY                 CCTCAACCTTTTCACCTATTTTCATTCCCTATGTAAACAG-------CTT
RT_sdY                      CCTAAACCTTTTCACCTATTTTCATTCCCTATATAAACAG-------CTT
                            ***  ******* **** ************** *** ***       ***

                                                                          <---
BT_sdY                      CCTGAGCTATAGTA-TGGCAC-----------------------------
AS_Tasmanian_Ssa02_sdY      CCTGAGCTATAGTATTGGCACTAT--------------------------
AS_Tasmanian_Ssa03_sdY      CCTGAGCTATAGTATTGGCACTAT--------------------------
AS_European_sdY             CCTGAGCTATAGTATTGGCACTATATTTCTTCCACCTCACACTAAGTAGA
AS_Tasmanian_Ssa06_sdY      CCTGAGCTATAGTATTGGCACTAT--------------------------
Chinook_sdY                 ATTGA------------GCACTATATTTATTCCATCTCACACTAAATAGA
RT_sdY                      ATTGA------------GCACTATATTTATTCCATCTCACACTAAATAGA
                              ***            ****
```

K. P. Lubieniecki *et al.*

```
                            --sdY 3'UTR--------
BT_sdY                      --------------------------------------------------
AS_Tasmanian_Ssa02_sdY      --------------------------------------------------
AS_Tasmanian_Ssa03_sdY      --------------------------------------------------
AS_European_sdY             AGACAATCAGCCAGTAAATAAA----------------------------
AS_Tasmanian_Ssa06_sdY      --------------------------------------------------
Chinook_sdY                 AGACATTCAGACAGTAAATTAATAAATACCCTTCAAATACATAATTTTAA
RT_sdY                      AGACAATCAGACAGTAAATTAATAAATACCCTTCA---------------


BT_sdY                      --------------------------------------------------
AS_Tasmanian_Ssa02_sdY      --------------------------------------------------
AS_Tasmanian_Ssa03_sdY      --------------------------------------------------
AS_European_sdY             --------------------------------------------------
AS_Tasmanian_Ssa06_sdY      --------------------------------------------------
Chinook_sdY                 TGTTGCTTTTTTGTCCATTTCAGATTAATGCTTACTTCATGCTGTTTGTT
RT_sdY                      --------------------------------------------------


BT_sdY                      ------------
AS_Tasmanian_Ssa02_sdY      ------------
AS_Tasmanian_Ssa03_sdY      ------------
AS_European_sdY             ------------
AS_Tasmanian_Ssa06_sdY      ------------
Chinook_sdY                 CTGTTACAGTTG
RT_sdY                      ------------
```

Figure S1 CLUSTAL 2.1 multiple sequence alignment of sdY from brown trout (BT), three Tasmanian and one European Atlantic salmon, Chinook salmon and rainbow trout (RT). The boundaries of exons and locations of primers are shown.

```
sdY                     MADREARFQAQHSFLVSVEYCEEEVLSHEVMGSDVRIAYKPFSLMMDGIPVISLPKPPDT
AS_Tasmanian_Ssa02      MVDREARFQAQHSFLVSVEYCEEEVLSHEVMGSDVRIAYKPFSLMMDGIPVISLPKPPDT
AS_Tasmanian_Ssa03      MVDREARFQAQHSFLVSVEYCEEEVLSHEVMGSDVRIAYKPFSLMMDGIPVISLPKPPDT
AS_European             MVDREARFQAQHSFLVSVEYCEEEVLSHEVMGSDVRIAYKPFSLMMDGIPVISLPKPPDT
AS_Tasmanian_Ssa06      MVDREARFQAQHSFLVSVEYCEEEVLSHEVMGSDVRIAYKPFSLMMDGIPVISLPKPPDT
Chinook_sdY             MADREARIQAQHCFLVSVEYCEEEVLSHEVMGGDVRIAHKT-SLMMDGIPFISLPKPPNT
RT_sdY                  MADREARIQAQHCFLVSVEYCEEEVLSHEVMGGDVRIAHKT-SLMMDGIPFISLPKPPNT
                        *.*****:****.*******************.*****:*. ********.*******:*

BT_sdY                  IPISSDRSTLSNLLSLMEGGVVLSSREEGIYAERHSQAIVSWMGGTGDEMHVMERDVDPV
AS_Tasmanian_Ssa02      IPISSDRSILSNLLSLMEGGVVLSSKEEGIYAERHSQAIVSWMGGTGDEMHVMERDVDPV
AS_Tasmanian_Ssa03      IPISSDRSILSNLLSLMEGGVVLSSKEEGIYAERHSQAIVSWMGGTGDEMHVMERDVDPV
AS_European             IPISSDRSILSNLLSLMEGGVVLSSKEEGIYAERHSQAIVSWMGGTGDEMHVMERDVDPV
AS_Tasmanian_Ssa06      IPISSDRSILSNLLSLMEGGVVLSSKEEGIYAERHSQAIVSWMGGTGDEMHVMERDVDPV
Chinook_sdY             LPISSDRSILSNLLSLMEGGVVLSSREEGIYAERHSQATVSWMGGTGDEMHVMDRDVDPV
RT_sdY                  LPISSDRSILSNLLSLMEGGVVLSSREEGIYAERHSQATVSWMGGTGDEMHVMERDVDPV
                        :******* ****************:************ **************:******

BT_sdY                  MLFNRETFRQELERFSRADGFQPQIGFSLWFGQDSSLSAPISISIKLPWAQQLFKQAHDF
AS_Tasmanian_Ssa02      MLFNRETFRQELERFSRADGFQPQIGFSLWFGQDSSLSAPISISIKLPWAQQLFKQAHDF
AS_Tasmanian_Ssa03      MLFNRETFRQELERFSRADGFQPQIGFSLWFGQDSSLSAPISISIKLPWAQQLFKQAHDF
AS_European             MLFNRETFRQELERFSRADGFQPQIGFSLWFGQDSSLSAPISISIKLPWAQQLFKQAHDF
AS_Tasmanian_Ssa06      MLFNRETFRQELERFSRADGFQPQIGFSLWFGQDSSLSAPISISIKLPWAQQLFKQAHDF
Chinook_sdY             MLFNRETFRQELERFARADGSQPQCGFSLWFGQDSSLSAPNFISIKLPWAQQLFKEVHDF
RT_sdY                  MLFNREHFRQELDRFARADGSQPQCGFSLWFGQDSSLSAPIFISIKLPWAQQLFKEVHDF
                        ****** *****:**:**** *** ***************  *************:.***
```

```
BT_sdY                  RIWLESSPVSPGV
AS_Tasmanian_Ssa02      RIWLESSPVSPGV
AS_Tasmanian_Ssa03      RIWLESSPVSPGV
AS_European             RIWLESSPVSPGV
AS_Tasmanian_Ssa06      RIWLESSPVSPGV
Chinook_sdY             RIWLESSPVSPGV
RT_sdY                  RIWLESSPVSPGV
                        *************
```

Figure S2 CLUSTAL 2.1 multiple sequence alignment of sdY from brown trout (BT), three Tasmanian and one European Atlantic salmon, Chinook Ssalmon and rainbow trout (RT).

```
Atlantic Salmon Ctg_2    TTGT-TCTTAG--GCAAC------------GAATGG-AT-------CATTTCTAGTGA--
Rainbow Trout            TTGTATTTTAACAGCAACAGTTCCAACCTAGACAGGTATATAAGGGTGTTTTTAATGAAA
Chinook Salmon           TTGTATTTTAACAGCAACAGTTCCAACCTAGACAGGTATATAAGGGTGTTTTTAATGAAA
                         **** * ***   *****            **  ** **         *** ** ***

Atlantic Salmon Ctg_2    ---TATT-CAGAGCAT------GGCTGTGATAAATTACATGTGGGTGCATTAGGAAACAG
Rainbow Trout            ATATATTTCAAGGTATTTCATTGTTTGTGTTGCATTTCTTG--GGGGCATAAGGGCAATG
Chinook Salmon           ATATATTTCAAGGTATTTCACTGTTTGTGTTGCATTTCTT--GGGGGCATAAGGGCAATG
                            **** **  * **      *  **** *   **   *   ** **** ***  *  *

Atlantic Salmon Ctg_2    AGGGTGCACCCAAATGGA----------------ACCCTATTC--GATATATAGTGCACT
Rainbow Trout            AAA-TGTACCAATATTGACATATAGTTGCACATTTTCCTACGCTTGGCTCCCAGGAAAAC
Chinook Salmon           AAAACGTACCAATATTGACATATAGTTGCATATTTTCCTACGCTTGGCTCCCAGGAAAAC
                         *    * *** *  * **                  ****  *  *      **   *

Atlantic Salmon Ctg_2    ATTTTTGGTTTA------------CAAAGAATTATAT--------TGTAGTGTCTTCTGT
Rainbow Trout            ATTTCTCATTTGGGTCCTAGGCTGAAAAAGTTTAAGAACCC----TGTAGTGTCTTCTGT
Chinook Salmon           ATTTCTCATTTGGGTCCTAGGCTGAAAAAGTTTAAGAACCCCTGCTGTAGTGTTTTCTGT
                         **** *  ***              ***   ***           ******** ******

Atlantic Salmon Ctg_2    CTTTATCCTGTCTGGTACAAGATAAAGTCTGCAAAGAGGAGGAGAAGGGAGAGAGAGTGA
Rainbow Trout            CTTTATCCTCCCTGGTACAAGATCAAGTCTGGAGAGAGGAG---AAGGGAGAGAG--TGA
Chinook Salmon           CTTTATCCTCCCTGGTACAAGATCAAGTCTGGAGAGTGGAG---AAGGGAGAGAGAGTGA
                         *********  ************ ******* * ** ****   ***********  ***

Atlantic Salmon Ctg_2    GCAAGGCCTTGGCCTTCACCTGACAAGAGAGGATTGAGAGAGAAACACTCCCCTTAACTA
Rainbow Trout            GCAAGGCCTTGGCCTTCACCTGACAAGAGAGGATTGAGAGAGAAACACTCCCCTTAACTA
Chinook Salmon           GCAAGGCCTTGGCCTTCACCTGACAAGAGAGGATTGAGAGAGAAACACTCCCCTTAACTA
                         ************************************************************

Atlantic Salmon Ctg_2    ATCATTTTACATGAAAAAATGCATATCAAAAACTCCAGCGCATGTTTAAAAACAGTCCTT
Rainbow Trout            ATACTTTTACATGAAAACATGCTCATAAAAAACTCCAGCTCATGTTTAAAAGCCGTCCTT
Chinook Salmon           ATACTTTTACATGAAAACATGCACATAAAAAACTCCAGCTCATGTTTAAAAGCCGTCCTT
                         **  ************* ****  ** ************ *********** * ******

                                                    |Exon 1
                                                    ATGGCTGACAGAGAGGCCAGAATCCAAG
Atlantic Salmon Ctg_2    AGAGGTTTTGAATCCACCTGCCCTTCAATGGTTGACAGAGAGGCCAGATTCCAAGGTACA
Rainbow Trout            AGAATATTTGAGTCCATCTGCCCTTCAATGGCTGACAGAGAGGCCAGAATCCAAGGTACA
Chinook Salmon           AGAATAGTTGAGTCCATCTGCCCTTCAATGGCTGACAGAGAGGCCAGAATCCAAGGTACA
                         ***   ***** ******************
```

Figure S3 CLUSTAL multiple sequence alignment by Kalign (2.0) of 5’ sdY sequences of Atlantic salmon, rainbow trout and Chinook salmon.

Table S1 Nucleotide sequences of primers and probes.

Primer name	Sequence 5' - 3'	Purpose
SDY exon1 F	ATGGTTGACAGAGAGGCCAGA	
SDY exon 1 R	CCATCATCAGGGAGAAGGGC	
SDY exon2 F probe	TGATGGATGGGATCCCCGTCATCTCTCTCCCAAAG	
SDY exon2 R	TAGAGCTTAAAACCACTCCACCCTCCATGAGGGA	
SDY exon3 F	AGTTGGAACGCTTCAGCAGAGCAGATGG	
SDY exon3 R	AGATTGGTGCACTGAGTGATGAGTCTTGTCC	
SDY exon4 F probe	GACTTCAGGATCTGGCTTGAGTCCTCCCCTGTCTCTCCTGGAG	
SDY exon4 R	ATTTACTGGCTGATTGTCTTCTA	
Contig 2 FISH 858 F	TGTGGGTGCATTAGGAAACA	cloning for FISH plasmid
Contig 2 FISH 5976 R	GACTGTGGGGCTGAGACAAT	cloning for FISH plasmid
Contig 2 6155 F	GGTAGCACTTGTAGCCAGAC	male-female boundary check
Contig 2 6450 R	TGGTGACATGGTGAGTAGTG	male-female boundary check
Contig 2 8297 F	CTTAGAATGTTGGGTTGGAG	male-female boundary check
Contig 2 8473 R	CATTCACAGATCCCAGTGC	male-female boundary check
Contig 2 14711 F	GGCATGTTTGTACAAGAACC	male-female boundary check
Contig 2 15014 R	ACAGGTGATGAAGTGAAAGC	male-female boundary check
Contig 2 18353 F	GAATGCAGCTTTCTTACCTG	male-female boundary check
Contig 2 18680 R	ATATGGTATGACCCAACCAG	male-female boundary check
Contig 1 95417 F	ATTGAACAGGTTACCAGGTG	male-female boundary check
Contig 1 95712 R	TCCTCACTCACACAAGACAA	male-female boundary check
Contig 1 86142 F	GATGCTGGGTAAGTCTGAAG	male-female boundary check
Contig 1 86552 R	CCATTTCCTCCAGGTAACTA	male-female boundary check

Contig 1 42083 F	AGGGAACCGTTGATCTACTT	male-female boundary check
Contig 1 42446 R	TACTGCACATTCTGGTGAAA	male-female boundary check
Contig 1 12665 F	AGAAATGTGGAGCAAGAAGA	microsatellite marker
Contig 1 12947 R	TGTAAAACGACGGCCAGTTGTGTATGCGTTTACTGCTT	microsatellite marker
Exon 1 F	ATGGTTGACAGAGAGGCCAGA	sequencing primer
Exon 1 R	CCATCATCAGGGAGAAGGGC	sequencing primer
Exon 2 F	CCCAGCACTCTTTTCTTGTCTC	sequencing primer
Exon 2 R	CCTGTCTGAAGGTCTCCCTG	sequencing primer
Exon 3 R	AGATTGGTGCACTGAGTGATGAGTCTT	sequencing primer
Exon 3 F	AGTTGGAACHCTTCGCCAGAGCAGATGG	sequencing primer
Exon 3-4 F2a	AAATTCAATTTATGGGACATG	sequencing primer
sdY 3' UTR R (Exon 4 R)	ATTTACTGGCTGATTGTCTTCTA	sequencing primer
sdY 5' UTR F (Exon 1 F)	AGAGTGAGCAAGGCCTTGG	sequencing primer
Salmon & BT 10bp gap R	GTCCGCCTATGGAAATCAATTG	sequencing primer
sdY Exon 3-intron-end F2	TATGAACACAACCAGTCATGATG	sequencing primer
