# Supplementary material for: Genomic Instability of the Sex-Determining Locus in Atlantic Salmon (Salmo salar)
Source: G3 (Bethesda). 2015 Sep 22;5(11):2513–22. doi: 10.1534/g3.115.020115 (PMC4632069; doi:10.1534/g3.115.020115)
Supplement: Supporting Information [file supp_g3.115.020115_FigureS1.pdf]

```
BT_sdY                      ----------CTCCCTT--ACTAATCATTTTACATGAAAAAATGCATATC
AS_Tasmanian_Ssa02_sdY      ----------CTCCCCTTAACTAATCATTTTACATGAAAAAATGCATATC
AS_Tasmanian_Ssa03_sdY      ---------ACTCCCCTT-ACTAATCATTTTACATGAAAAAATGCATATC
AS_European_sdY             GAGAGAAACACTCCCCTTAACTAATCATTTTACATGAAAAAATGCATATC
AS_Tasmanian_Ssa06_sdY      ---------ACTCCCCTTAACTAATCATTTTACATGAAGAAATGCATATC
Chinook_sdY                 ----------CTCCCCTTAACTAATACTTTTACATGAAAACATGCACATA
RT_sdY                      ------AACACTCCCCTTAACTAATACTTTTACATGAAAACATGCTCATA
                                      ***** *  ******  *********** * ****  **

BT_sdY                      AAAAACTCCAGCGCATGTTTAAAAGCAGTCCTTAGAGGTTTTGAATCCAC
AS_Tasmanian_Ssa02_sdY      AAAAACTCCAGCGCATGTTTAAAAACAGTCCTTAGAGGTTTTGAATCCAC
AS_Tasmanian_Ssa03_sdY      AAAAACTCCAGCGCATGTTTAAAAACAGTCCTTAGAGGTTTTGAATCCAC
AS_European_sdY             AAAAACTCCAGCGCATGTTTAAAAACAGTCCTTAGAGGTTTTGAATCCAC
AS_Tasmanian_Ssa06_sdY      AAAAACTCCAGCGCATGTTTAAAAACAGTCCTTAGAGGTTTTGAATCCAC
Chinook_sdY                 AAAAACTCCAGCTCATGTTTAAAAGCCGTCCTTAGAATAGTTGAGTCCAT
RT_sdY                      AAAAACTCCAGCTCATGTTTAAAAGCCGTCCTTAGAATATTTGAGTCCAT
                            ************ *********** * *********    **** ****
                                      |      exon1             |
                                      |-------Exon 1F------>       |
BT_sdY                      CTGCCCTTCA|ATGGCTGACAGAGAGGCCAGATTCCAAG|GTACAGAACACA
AS_Tasmanian_Ssa02_sdY      CTGCCCTTCA|ATGGTTGACAGAGAGGCCAGATTCCAAG|GTACAGAACACA
AS_Tasmanian_Ssa03_sdY      CTGCCCTTCA|ATGGTTGACAGAGAGGCCAGATTCCAAG|GTACAGAACACA
AS_European_sdY             CTGCCCTTCA|ATGGTTGACAGAGAGGCCAGATTCCAAG|GTACAGAACACA
AS_Tasmanian_Ssa06_sdY      CTGCCCTTCA|ATGGTTGACAGAGAGGCCAGATTCCAAG|GTACAGAACACA
Chinook_sdY                 CTGCCCTTCA|ATGGCTGACAGAGAGGCCAGAATCCAAG|GTACAGAACACA
RT_sdY                      CTGCCCTTCA|ATGGCTGACAGAGAGGCCAGAATCCAAG|GTACAGAACACA
                            **********|**** **************** ******|************
```

K. P. Lubieniecki *et al.*

```
BT_sdY                    CAAATATTCAGTATGTTATCTAAGAGTGTTTTGATTTTCATTATTTTGGG
AS_Tasmanian_Ssa02_sdY    CAAATATTCAGTATGTTATCTAAGAGTGTTTTGATTTTCATTATTTTGGG
AS_Tasmanian_Ssa03_sdY    CAAATATTCAGTATGTTATCTAAGAGTGTTTTGATTTTCATTATTTTGGG
AS_European_sdY           CAAATATTCAGTATGTTATCTAAGAGTGTTTTGATTTTCATTATTTTGGG
AS_Tasmanian_Ssa06_sdY    CAAATATTCAGTATGTTATCTAAGAGTGTTTTGATTTTCATTATTTTGGG
Chinook_sdY               CAAACATTCAGTATGTTATGTAAGACTG-TTTGGTTTTCATTATTTTGGG
RT_sdY                    CAAACATTCAGTATGTTATGTAAGACTG-TTTGGTTTTCATTATTTTGGG
                          **** ************** ***** ** **** ****************

BT_sdY                    CCTATGAATTTCTGATGTTAA----------GGACTGTATCTATGTGGTT
AS_Tasmanian_Ssa02_sdY    CCTATGCATTTCTGATGTTGAACAGCTTAA-GGACTGTGTCTATGTGGTT
AS_Tasmanian_Ssa03_sdY    CCTATGCATTTCTGATGTTGAACAGCTTAA-GGACTGTGTCTATGTGGTT
AS_European_sdY           CCTATGCATTTCTGATGTTGAACAG---AA-GGACTGTGTCTATGTGGTT
AS_Tasmanian_Ssa06_sdY    CCTATGCATTTCTGATGTTGAACAGCTTAA-GGACTGTGTCTATGTGGTT
Chinook_sdY               CCTATGAATGTCTGATGTTATACAGCTTACTGTGCTGTGTACATGTGGTT
RT_sdY                    CCTATGAATGTCTGATGTTGTACAGCTTACTGTGCTGTGTATATGTGGTT
                          ****** ** *********            *  **** *  ********

BT_sdY                    ATTTCCATGTCTGATCTTTTGAGGGAAATGCTCAAAATGAGTATATAGCA
AS_Tasmanian_Ssa02_sdY    ATTTCCATGTCTGATCTTTTGAGGGA-------AAAATGAGTATATAGCG
AS_Tasmanian_Ssa03_sdY    ATTTCCATGTCTGATCTTTTGAGGGA-------AAAATGAGTATATAGCG
AS_European_sdY           ATTTCCATGTCTGATCTTTTGAGGGAAATGCTCAAAATGAGTATATAGCG
AS_Tasmanian_Ssa06_sdY    ATTTCCATGTCTGATCTTTTGAGGGAAATGCTCAAAATGAGTATATAGCG
Chinook_sdY               ATTTCCATGTCTGATTTC-------------------------CATGTCT
RT_sdY                    ATTTCCATGTCTGA------------------------------------
                          **************
```

```
BT_sdY                      TTTTGGAATTAG---CTTCAGTTGTACAATTTCATTACTATACGTTTTTT
AS_Tasmanian_Ssa02_sdY      TTTTGGAATTAG---CTTCAGTTGTACAATTTCATTACTATACTTTTTTT
AS_Tasmanian_Ssa03_sdY      TTTTGGAATTAG---CTTCAGTTGTACAATTTCATTACTATACTTTTTTT
AS_European_sdY             TTTTGGAATTAG---CTTCAGTTGTACAATTTCATTACTATACTTTTTTT
AS_Tasmanian_Ssa06_sdY      TTTTGGAATTAG---CTTCAGTTGTACAATTTCATTACTATACTTTTTTT
Chinook_sdY                 AATTGGAATGAAACTTTTCAGTTGTACAATTTCATTACTTAACTTTTTTT
RT_sdY                      --TTGGAATGAAACTCTTCAGTTGTACAATTTCATTATTTAACTTTTTTT
                              ******* *     ********************* *  ** ******

BT_sdY                      GTGCTAATTTTATAATGTTCATGTCGCAAATCTGTTATAAAAAGTCAGGA
AS_Tasmanian_Ssa02_sdY      GTGCTAATTTTATAATGTTCATGTTGCAAATCTGTTATAAAAAGTCAGGA
AS_Tasmanian_Ssa03_sdY      GTGCTAATTTTATAATGTTCATGTTGCAAATCTGTTATAAAAAGTCAGGA
AS_European_sdY             GTGCTAATTTTATAATGTTCATGTTGCAAATCTGTTATAAAAAGTCAGGA
AS_Tasmanian_Ssa06_sdY      GTGCTAATTTTATAATGTTCATGTTGCAAATCTGTTATAAAAAGTCAGGA
Chinook_sdY                 GTGCTCATT------GGATCATATCACAAATCTCTTATAAAAAGTCAGGA
RT_sdY                      GTGCTCATT------GGATCATATCACAAATCTCTTATAAAAAGTCAGGA
                            ***** ***       * **** *  ******* ****************

BT_sdY                      GCTGTTCTTCTGCGAGA---CTCATTGAAATTATTTTTTTCTCTCTCTCC
AS_Tasmanian_Ssa02_sdY      GCTGTTCTTCTGCGGGACTGCTCATTGAAATTTTGTTTTTCTCTCTCTCT
AS_Tasmanian_Ssa03_sdY      GCTGTTCTTCTGCGGGACTGCTCATTGAAATTTTGTTTTTCTCTCTCTCT
AS_European_sdY             GCTGTTCTTCTGCGAGACTGCTCATTGAAATTTTGTTTTTCTCTCTCTCT
AS_Tasmanian_Ssa06_sdY      GCTGTTCTTCTGCGAGACTGCTCATTGAAACTTTGTTTTTCTCTCTCTCT
Chinook_sdY                 GCTGTTCTTCTGCATAACTGCTCATTGCAAGTA---TTTTTTATCTC---
RT_sdY                      GCTGTTCTTCTGCATAACTGCTCATTGCAAGTATTTTTTTTTATCTC---
                            *************   *   ******* ** *    **** * ****
```

```
BT_sdY                    CCC-----------------------------------------------
AS_Tasmanian_Ssa02_sdY    CCCTCTCTCCCCCCTCTCTCTCTCTCTCTCTCTCTCTCTCCCTAACTCTC
AS_Tasmanian_Ssa03_sdY    CCCTCTCTCCCCC--CTCTCTCTCTCTCTCTCTCTCTCTCCCTAACTCTC
AS_European_sdY           CCCTCTCTCCCCA-------------------------------------
AS_Tasmanian_Ssa06_sdY    CCCTCTCTCCCCC--------CCCTCTCTCTCTCTCTCTCCCTAACTCTC
Chinook_sdY               --------------------------------------------------
RT_sdY                    --------------------------------------------------

BT_sdY                    -----CCCTCTCTCTCTCTCTCTATTTCTCTGTCTCTCCCTAACTCTCTC
AS_Tasmanian_Ssa02_sdY    TCTCTCCCTCTCTCTCTCTCTTTCTCTCTCTCTCTCTCCCTCTCTCTCTC
AS_Tasmanian_Ssa03_sdY    TCTCTCCCTCTCTCTCTCTCTTTCTCTCTCTCTCTCTCCCTCTCTCTCTC
AS_European_sdY           -------------CTCTCTCTCTCTCTCTCTCTCTCTCCCTAACTCTCTC
AS_Tasmanian_Ssa06_sdY    TCTC-CCCTCTCTCTCTCTCTTTCTCTCTCTCTCTCTCCCTCTCTCTCTC
Chinook_sdY               --------------------------------------------------
RT_sdY                    --------------------------------------------------

BT_sdY                    TCTCCCTCTCTCTCTCT----------------------------CTCCC
AS_Tasmanian_Ssa02_sdY    TCTCTCTCTCTCTCTCTCTCTCTATCACTCTCCCTCTCTCTCTCTCTCCC
AS_Tasmanian_Ssa03_sdY    TCTCTCTCTCTCTCTCTCTCTCTATCACTCTCCCTCTCTCTCTCTCTCCC
AS_European_sdY           TCTCCCTCTCTCTCTCT---------------------------------
AS_Tasmanian_Ssa06_sdY    TCTCTCTCTCTCTCTCT----------CTCTCACTCTCCCTCTCTCTCTC
Chinook_sdY               --------------------------------------------------
RT_sdY                    --------------------------------------------------

BT_sdY                    TCTCTCTCTCTCTCTCCCTCCCTCTCTCTCTCTTTCTCTCTCTCTCTCTC
AS_Tasmanian_Ssa02_sdY    TCCCT--CTCTCTCTCTCTCTCTCTCTCTCTCTCTCTCTCTCTCTCTCTC
AS_Tasmanian_Ssa03_sdY    TCCCTCTCTCTCTCTCTCTCTCTCTCTCTCTCTCTCTCTCTCTCTCTCTC
AS_European_sdY           ---------------------------CTCTCTCTCTCTCTCTCTCTCTC
AS_Tasmanian_Ssa06_sdY    TCCCTCCCTCTCTCTCTCTCTCTCTCTCTCTCTCTCTCTCTCTCTCTCTC
Chinook_sdY               ---------------------------------CTCCCTCTA--------
RT_sdY                    ---------------------------------CTCCCTCTATCTCTGTC
```

```
                                                                              |----
BT_sdY                            TCCTCCCTCTCTTTCTATGTCCCTCTGTCACTCTCTCTCTCCCCAG|CCCA
AS_Tasmanian_Ssa02_sdY            TCCTCCCTCTCTTTCTATGTCCCTCTGTCACTCTCTCTCTCCCCAG|CCCA
AS_Tasmanian_Ssa03_sdY            TCCTCCCTCTCTTTCTATGTCCCTCTGTCACTCTCTCTCTCCCCAG|CCCA
AS_European_sdY                   TCCTCCCTCTCTTTCTATGTCCCTCTGTCACTCTCTCTCTCCCCAG|CCCA
AS_Tasmanian_Ssa06_sdY            TCCTCCCTCTCTTTCTATGTCCCTCTGTCACTCTCTCTCTCCCCAG|CCCA
Chinook_sdY                       -----------------------------------TCTCTCCCCAG|CCCA
RT_sdY                            TGTTCCCTCTGTT--------------------TCTCTCTCCCCAG|CCCA
                                                                     ***********|****

                                    exon2
                                  --Exon 2F-------->
BT_sdY                            GCACTCTTTTCTTGTCTCAGTGGAGTACTGCGAAGAGGAGGTGCTTAGTC
AS_Tasmanian_Ssa02_sdY            GCACTCTTTTCTTGTCTCAGTGGAGTACTGCGAAGAGGAGGTGCTTAGTC
AS_Tasmanian_Ssa03_sdY            GCACTCTTTTCTTGTCTCAGTGGAGTACTGCGAAGAGGAGGTGCTTAGTC
AS_European_sdY                   GCACTCTTTTCTTGTCTCAGTGGAGTACTGCGAAGAGGAGGTGCTTAGTC
AS_Tasmanian_Ssa06_sdY            GCACTCTTTTCTTGTCTCAGTGGAGTACTGCGAAGAGGAGGTGCTTAGTC
Chinook_sdY                       GCACTGTTTTCTTGTCTCAGTGGAGTACTGCGAAGAGGAGGTGCTTAGTC
RT_sdY                            GCACTGTTTTCTTGTCTCAGTGGAGTACTGCGAAGAGGAGGTGCTTAGTC
                                  ***** ********************************************


                                    exon2
                                                                       <----Exon 1R-
BT_sdY                            ATGAGGTTATGGGGAGTGATGTCAGAATTGCCTACAAGCCCTTCTCCCTG
AS_Tasmanian_Ssa02_sdY            ATGAGGTTATGGGGAGTGATGTCAGAATTGCCTACAAGCCCTTCTCCCTG
AS_Tasmanian_Ssa03_sdY            ATGAGGTTATGGGGAGTGATGTCAGAATTGCCTACAAGCCCTTCTCCCTG
AS_European_sdY                   ATGAGGTTATGGGGAGTGATGTCAGAATTGCCTACAAGCCCTTCTCCCTG
AS_Tasmanian_Ssa06_sdY            ATGAGGTTATGGGGAGTGATGTCAGAATTGCCTACAAGCCCTTCTCCCTG
Chinook_sdY                       ATGAGGTCATGGGGGGTGATGTCAGAATTGCCCACAAGAC---CTCCCTA
RT_sdY                            ATGAGGTCATGGGGGGTGATGTCAGAATTGCCCACAAGAC---CTCCCTA
                                  ******* ****** ***************** ***** *   ******
```

```
                              exon2
                            -------
BT_sdY                      ATGATGGATGGGATCCCCGTCATCTCTCTCCCAAAGCCCCCCGACACCAT
AS_Tasmanian_Ssa02_sdY      ATGATGGATGGCATCCCCGTCATCTCTCTCCCAAAGCCCCCCGACACCAT
AS_Tasmanian_Ssa03_sdY      ATGATGGATGGCATCCCCGTCATCTCTCTCCCAAAGCCCCCCGACACCAT
AS_European_sdY             ATGATGGATGGGATCCCCGTCATCTCTCTCCCAAAGCCCCCCGACACCAT
AS_Tasmanian_Ssa06_sdY      ATGATGGATGGGATCCCCGTCATCTCTCTCCCAAAGCCCCCCGACACCAT
Chinook_sdY                 ATGATGGATGGGATCCCCTTCATTTCTCTCCCAAAGCCCCCCAACACCCT
RT_sdY                      ATGATGGATGGGATCCCCTTCATCTCTCTCCCAAAGCCCCCCAACACCCT
                            *********** ****** **** ****************** ***** *

                              exon2
BT_sdY                      TCCCATCTCCTCTGACCGTTCAACCCTCTCCAACCTGCTTTCCCTCATGG
AS_Tasmanian_Ssa02_sdY      TCCCATCTCCTCTGACCGTTCAATCCTCTCCAACCTGCTTTCCCTCATGG
AS_Tasmanian_Ssa03_sdY      TCCCATCTCCTCTGACCGTTCAATCCTCTCCAACCTGCTTTCCCTCATGG
AS_European_sdY             TCCCATCTCCTCTGACCGTTCAATCCTCTCCAACCTGCTTTCCCTCATGG
AS_Tasmanian_Ssa06_sdY      TCCCATCTCCTCTGACCGTTCAATCCTCTCCAACCTGCTTTCCCTCATGG
Chinook_sdY                 TCCTATCTCCTCTGATCGTTCAATCCTCTCCAACCTGTTGTCCCTCATGG
RT_sdY                      TCCTATCTCCTCTGATCGTTCAATCCTCTCCAACCTGTTGTCCCTCATGG
                            *** *********** ******* ************* * **********

                              exon2
BT_sdY                      AGGGTGGAGTGGTTTTAAGCTCTAGGGAGGAAGGCATCTATGCTGAACGG
AS_Tasmanian_Ssa02_sdY      AGGGTGGAGTGGTTTTAAGCTCTAAGGAGGAAGGCATCTATGCTGAACGG
AS_Tasmanian_Ssa03_sdY      AGGGTGGAGTGGTTTTAAGCTCTAAGGAGGAAGGCATCTATGCTGAACGG
AS_European_sdY             AGGGTGGAGTGGTTTTAAGCTCTAAGGAGGAAGGCATCTATGCTGAACGG
AS_Tasmanian_Ssa06_sdY      AGGGTGGAGTGGTTTTAAGCTCTAAGGAGGAAGGCATCTATGCTGAACGG
Chinook_sdY                 AGGGTGGAGTAGTTTTAAGCTCTAGGGAGGAAGGTATCTATGCTGAACGG
RT_sdY                      AGGGTGGAGTGGTTTTAAGCTCTAGGGAGGAAGGTATCTATGCTGAACGG
                            ********** ************* ********* ***************
```

```
                              exon2
BT_sdY                      CATAGCCAAGCCATAGTCTCCTGGATGGGCGGCACGGGGGATGAGATGCA
AS_Tasmanian_Ssa02_sdY      CATAGCCAAGCCATAGTCTCCTGGATGGGCGGCACGGGGGATGAGATGCA
AS_Tasmanian_Ssa03_sdY      CATAGCCAAGCCATAGTCTCCTGGATGGGCGGCACGGGGGATGAGATGCA
AS_European_sdY             CATAGCCAAGCCATAGTCTCCTGGATGGGCGGCACGGGGGATGAGATGCA
AS_Tasmanian_Ssa06_sdY      CATAGCCAAGCCATAGTCTCCTGGATGGGCGGCACGGGGGATGAGATGCA
Chinook_sdY                 CATAGCCAAGCCACAGTCTCCTGGATGGGTGGCACCGGAGATGAGATGCA
RT_sdY                      CATAGCCAAGCCACAGTCTCCTGGATGGGTGGCACCGGAGATGAGATGCA
                            ************* *************** ***** ** ***********
                              exon2
                                                                       <--Exon 2R-
BT_sdY                      CGTGATGGAGCGTGATGTGGATCCTGTGATGCTCTTCAACAGGGAGACCT
AS_Tasmanian_Ssa02_sdY      CGTGATGGAGCGTGATGTGGATCCTGTGATGCTCTTCAACAGGGAGACCT
AS_Tasmanian_Ssa03_sdY      CGTGATGGAGCGTGATGTGGATCCTGTGATGCTCTTCAACAGGGAGACCT
AS_European_sdY             CGTGATGGAGCGTGATGTGGATCCTGTGATGCTCTTCAACAGGGAGACCT
AS_Tasmanian_Ssa06_sdY      CGTGATGGAGCGTGATGTGGATCCTGTGATGCTCTTCAACAGGGAGACCT
Chinook_sdY                 CGTGATGGACCGTGATGTGGACCCTGTGATGCTCTTCAACAGAGAGACCT
RT_sdY                      CGTGATGGAGCGTGATGTGGACCCTGTGATGCTCTTCAACAGAGAGCACT
                            ********* *********** ******************** ***  **


                            ---------|
BT_sdY                      TCAGACAGG|GTGAGTGA---------------------------------
AS_Tasmanian_Ssa02_sdY      TCAGACAGG|GTGAGTGA---------------------------------
AS_Tasmanian_Ssa03_sdY      TCAGACAGG|GTGAGTGA---------------------------------
AS_European_sdY             TCAGACAGG|GTGAGTGA---------------------------------
AS_Tasmanian_Ssa06_sdY      TCAGACAGG|GTGAGTGA---------------------------------
Chinook_sdY                 TCAGACAGG|GTGAGTGAACCTGTTAGTGGTAAATTCTAATGATTCTTGAA
RT_sdY                      TCAGACAGG|GTGAGTGAATCTGTTAGTGGTAAATTCTAATGATTCTTGAA
                            *********|********
```

```
BT_sdY                     --------------------------------------------------
AS_Tasmanian_Ssa02_sdY     --------------------------------------------------
AS_Tasmanian_Ssa03_sdY     --------------------------------------------------
AS_European_sdY            --------------------------------------------------
AS_Tasmanian_Ssa06_sdY     --------------------------------------------------
Chinook_sdY                GAGCATAACTTATAATAGGGAGGATGTTCCCTCGGGGGGATCAAATCAGC
RT_sdY                     GAGCATAACTTATAATAG--------------------------------

BT_sdY                     --------------------------------------------------
AS_Tasmanian_Ssa02_sdY     --------------------------------------------------
AS_Tasmanian_Ssa03_sdY     --------------------------------------------------
AS_European_sdY            --------------------------------------------------
AS_Tasmanian_Ssa06_sdY     --------------------------------------------------
Chinook_sdY                GGGTAGTCAGCGGATATTTTAGAGCGTCACTGATCGTTTTTCGATAAAAT
RT_sdY                     --------------------------------------------------

BT_sdY                     --------------------------------------------------
AS_Tasmanian_Ssa02_sdY     --------------------------------------------------
AS_Tasmanian_Ssa03_sdY     --------------------------------------------------
AS_European_sdY            --------------------------------------------------
AS_Tasmanian_Ssa06_sdY     --------------------------------------------------
Chinook_sdY                TCAAACTTTCATTAAAACACACATGCAAGGTATTGAATTAAAGCTACACT
RT_sdY                     --------------------------------------------------

BT_sdY                     --------------------------------------------------
AS_Tasmanian_Ssa02_sdY     --------------------------------------------------
AS_Tasmanian_Ssa03_sdY     --------------------------------------------------
AS_European_sdY            --------------------------------------------------
AS_Tasmanian_Ssa06_sdY     --------------------------------------------------
Chinook_sdY                CGTTGTGAATCTAGTCAGATTTGTAAAATGCTTTTCGGCGAAAGCATGAG
RT_sdY                     --------------------------------------------------
```

```
BT_sdY                     --------------------------------------------------
AS_Tasmanian_Ssa02_sdY     --------------------------------------------------
AS_Tasmanian_Ssa03_sdY     --------------------------------------------------
AS_European_sdY            --------------------------------------------------
AS_Tasmanian_Ssa06_sdY     --------------------------------------------------
Chinook_sdY                AAGCTATTATCTGATAGCATGTAACACCCCAAAAGACCCGTAGAGGATGT
RT_sdY                     --------------------------------------------------

BT_sdY                     --------------------------------------------------
AS_Tasmanian_Ssa02_sdY     --------------------------------------------------
AS_Tasmanian_Ssa03_sdY     --------------------------------------------------
AS_European_sdY            --------------------------------------------------
AS_Tasmanian_Ssa06_sdY     --------------------------------------------------
Chinook_sdY                AAACAAAAATAATTAGCGTAGTCGGCGCTACACAAACCGCACAATAAAAT
RT_sdY                     --------------------------------------------------

BT_sdY                     --------------------------------------------------
AS_Tasmanian_Ssa02_sdY     --------------------------------------------------
AS_Tasmanian_Ssa03_sdY     --------------------------------------------------
AS_European_sdY            --------------------------------------------------
AS_Tasmanian_Ssa06_sdY     --------------------------------------------------
Chinook_sdY                ATAAAACATTCATTACCTTTGACCATCTTCTTTCTTGGCACTCCTTGATG
RT_sdY                     --------------------------------------------------

BT_sdY                     --------------------------------------------------
AS_Tasmanian_Ssa02_sdY     --------------------------------------------------
AS_Tasmanian_Ssa03_sdY     --------------------------------------------------
AS_European_sdY            --------------------------------------------------
AS_Tasmanian_Ssa06_sdY     --------------------------------------------------
Chinook_sdY                TCCCATAATCAATACTGGGTCTTTTTTTGGATTAAATCGGTCCATATATA
RT_sdY                     --------------------------------------------------
```

```
BT_sdY                      --------------------------------------------------
AS_Tasmanian_Ssa02_sdY      --------------------------------------------------
AS_Tasmanian_Ssa03_sdY      --------------------------------------------------
AS_European_sdY             --------------------------------------------------
AS_Tasmanian_Ssa06_sdY      --------------------------------------------------
Chinook_sdY                 GCCTAGATATCGATCTATGAAGACTGTGTGATAAACGGAAAAAAATAGCG
RT_sdY                      --------------------------------------------------

BT_sdY                      --------------------------------------------------
AS_Tasmanian_Ssa02_sdY      --------------------------------------------------
AS_Tasmanian_Ssa03_sdY      --------------------------------------------------
AS_European_sdY             --------------------------------------------------
AS_Tasmanian_Ssa06_sdY      --------------------------------------------------
Chinook_sdY                 TTTCATAACGTAACGTCATTTTTTAAAAGTTAAAAAGTCGACGATAAACT
RT_sdY                      --------------------------------------------------

BT_sdY                      --------------------------------------------------
AS_Tasmanian_Ssa02_sdY      --------------------------------------------------
AS_Tasmanian_Ssa03_sdY      --------------------------------------------------
AS_European_sdY             --------------------------------------------------
AS_Tasmanian_Ssa06_sdY      --------------------------------------------------
Chinook_sdY                 TTCACAAAACACTTCGAAATACCTTTCTAATGCAACTTTAGGTATTACTA
RT_sdY                      --------------------------------------------------

BT_sdY                      --------------------------------------------------
AS_Tasmanian_Ssa02_sdY      --------------------------------------------------
AS_Tasmanian_Ssa03_sdY      --------------------------------------------------
AS_European_sdY             --------------------------------------------------
AS_Tasmanian_Ssa06_sdY      --------------------------------------------------
Chinook_sdY                 CACGTTAATAAGCTATAAAAATCATCAGGAGGCGATGTAAATTCGATAGT
RT_sdY                      --------------------------------------------------
```

```
BT_sdY                        --------------------------------------------------
AS_Tasmanian_Ssa02_sdY        --------------------------------------------------
AS_Tasmanian_Ssa03_sdY        --------------------------------------------------
AS_European_sdY               --------------------------------------------------
AS_Tasmanian_Ssa06_sdY        --------------------------------------------------
Chinook_sdY                   TGGCGTGTGGAAAAAATGTCCGGAAAAACACAGAGACAATGCCTCAGGTC
RT_sdY                        --------------------------------------------------

BT_sdY                        --------------------------------------------------
AS_Tasmanian_Ssa02_sdY        --------------------------------------------------
AS_Tasmanian_Ssa03_sdY        --------------------------------------------------
AS_European_sdY               --------------------------------------------------
AS_Tasmanian_Ssa06_sdY        --------------------------------------------------
Chinook_sdY                   GGTGGTCGGAGGGAATCGGTTCCCTTTGGTCGGATCTACCAAGAATCAAA
RT_sdY                        --------------------------------------------------

BT_sdY                        --------------------------------------------------
AS_Tasmanian_Ssa02_sdY        --------------------------------------------------
AS_Tasmanian_Ssa03_sdY        --------------------------------------------------
AS_European_sdY               --------------------------------------------------
AS_Tasmanian_Ssa06_sdY        --------------------------------------------------
Chinook_sdY                   TCAGAATCAAATGAGGAGACTCTATACATCCTGTGGAAGCTGTAGGTACT
RT_sdY                        --------------------------------------------------

BT_sdY                        --------------------------------------------------
AS_Tasmanian_Ssa02_sdY        --------------------------------------------------
AS_Tasmanian_Ssa03_sdY        --------------------------------------------------
AS_European_sdY               --------------------------------------------------
AS_Tasmanian_Ssa06_sdY        --------------------------------------------------
Chinook_sdY                   GCAAGCTCGGCCTCATTTAATACGGTTCACCTTTAACAATTCATGGAAGT
RT_sdY                        --------------------------------------------------
```

```
BT_sdY                   --------------------------------------------------
AS_Tasmanian_Ssa02_sdY   --------------------------------------------------
AS_Tasmanian_Ssa03_sdY   --------------------------------------------------
AS_European_sdY          --------------------------------------------------
AS_Tasmanian_Ssa06_sdY   --------------------------------------------------
Chinook_sdY              GGCGCATGGATATTTTTTTCCATCTCCAGTKATCAGATTTTCCTGCGCTT
RT_sdY                   --------------------------------------------------

BT_sdY                   --------------------------------------------------
AS_Tasmanian_Ssa02_sdY   --------------------------------------------------
AS_Tasmanian_Ssa03_sdY   --------------------------------------------------
AS_European_sdY          --------------------------------------------------
AS_Tasmanian_Ssa06_sdY   --------------------------------------------------
Chinook_sdY              TTCGATGAAACAGACGTTCTGTTATAGTCACAGCCGTGATTTAAACAGTT
RT_sdY                   --------------------------------------------------

BT_sdY                   --------------------------------------------------
AS_Tasmanian_Ssa02_sdY   --------------------------------------------------
AS_Tasmanian_Ssa03_sdY   --------------------------------------------------
AS_European_sdY          --------------------------------------------------
AS_Tasmanian_Ssa06_sdY   --------------------------------------------------
Chinook_sdY              TTAGAAACGTCTGAGTGTTTTCTATCCACACATACTAATCATATGCATAT
RT_sdY                   --------------------------------------------------

BT_sdY                   --------------------------------------------------
AS_Tasmanian_Ssa02_sdY   --------------------------------------------------
AS_Tasmanian_Ssa03_sdY   --------------------------------------------------
AS_European_sdY          --------------------------------------------------
AS_Tasmanian_Ssa06_sdY   --------------------------------------------------
Chinook_sdY              ACTATATTCCTGGAATGAGTAGCAGGGCGCTGAAATGTTGCGCGATTTTT
RT_sdY                   --------------------------------------------------
```

```
BT_sdY                    --------------------------------------------------
AS_Tasmanian_Ssa02_sdY    --------------------------------------------------
AS_Tasmanian_Ssa03_sdY    --------------------------------------------------
AS_European_sdY           --------------------------------------------------
AS_Tasmanian_Ssa06_sdY    --------------------------------------------------
Chinook_sdY               AACAAAAAGCTGCGAAAATTCGCAGCCTCCCCAAGAGGTTTTAATTAAAC
RT_sdY                    --------------------------------------------TTAAAC

BT_sdY                    --------------------------------------------------
AS_Tasmanian_Ssa02_sdY    --------------------------------------------------
AS_Tasmanian_Ssa03_sdY    --------------------------------------------------
AS_European_sdY           --------------------------------------------------
AS_Tasmanian_Ssa06_sdY    --------------------------------------------------
Chinook_sdY               AACCCAAAATATAAGCTTATTTAAATCCTATGTTTGTGAACTTTGTAAAT
RT_sdY                    AACCCAAAATATAAGCTTGTTTAAATCCTATGTTTGTGAACTTTGTAAAT

BT_sdY                    --------------------------------------------------
AS_Tasmanian_Ssa02_sdY    --------------------------------------------------
AS_Tasmanian_Ssa03_sdY    --------------------------------------------------
AS_European_sdY           --------------------------------------------------
AS_Tasmanian_Ssa06_sdY    --------------------------------------------------
Chinook_sdY               GTAAACAAACACTATATAGCCTTAAAAATGTTACTTATGTGATAATGCCT
RT_sdY                    GTAAACAAACACTATATAGCCTTAAAAATGTTACTTATGTGATAATGCCT

BT_sdY                    --------------------------------------------------
AS_Tasmanian_Ssa02_sdY    --------------------------------------------------
AS_Tasmanian_Ssa03_sdY    --------------------------------------------------
AS_European_sdY           --------------------------------------------------
AS_Tasmanian_Ssa06_sdY    --------------------------------------------------
Chinook_sdY               GAAAAAATGGTGTTTGGAGGGTATATTGGCACAGTGTTGTTAGGCCTATA
RT_sdY                    GAAAAAACAGTGTTTGGAGGGTATATTGGCGCAGTGTTGTTAGGCCAATA
```

```
BT_sdY                      ------------------------------------------------TA
AS_Tasmanian_Ssa02_sdY      ------------------------------------------------TA
AS_Tasmanian_Ssa03_sdY      ------------------------------------------------TA
AS_European_sdY             ------------------------------------------------TA
AS_Tasmanian_Ssa06_sdY      ------------------------------------------------TA
Chinook_sdY                 ATTTTGATAAATCTATTCATACTATTTCATCCTTCTATTTCATCCTTCTA
RT_sdY                      ATTTTGATAAATGTATTCATAC--------------------------TA
                                                                            **

BT_sdY                      TG------------------------------------------------
AS_Tasmanian_Ssa02_sdY      TT------------------------------------------------
AS_Tasmanian_Ssa03_sdY      TT------------------------------------------------
AS_European_sdY             TT------------------------------------------------
AS_Tasmanian_Ssa06_sdY      TT------------------------------------------------
Chinook_sdY                 TTTCATCCTTCCACAAGATATAGTCCCGACACAACCATATGGTTGCTACC
RT_sdY                      TTTCATCCTTCCACAAGATATAGTCCCGACACAACCATATGGTTGCTACC
                            *

BT_sdY                      --------------------------------------------------
AS_Tasmanian_Ssa02_sdY      --------------------------------------------------
AS_Tasmanian_Ssa03_sdY      --------------------------------------------------
AS_European_sdY             --------------------------------------------------
AS_Tasmanian_Ssa06_sdY      --------------------------------------------------
Chinook_sdY                 CAAACTGGATGGTCATCCATTGGTTTTGGTTGCCAAAGACACAACCCAGT
RT_sdY                      CAAACTGGATGGTCATCCATTGG-TTTGGTTGCCAAAGACGCTACCCAGT

BT_sdY                      --------------------------------------------------
AS_Tasmanian_Ssa02_sdY      --------------------------------------------------
AS_Tasmanian_Ssa03_sdY      --------------------------------------------------
AS_European_sdY             --------------------------------------------------
AS_Tasmanian_Ssa06_sdY      --------------------------------------------------
Chinook_sdY                 TGTTCAGATTTTTTGTTCTGTATCTATGGACACGACCTGGTTGTTCATTT
RT_sdY                      TGTTCAGATTTTTTGTTCTGTAACTATGGACACGACCCGGTTGTTCATTT
```

```
BT_sdY                      --------------------------------------------------
AS_Tasmanian_Ssa02_sdY      --------------------------------------------------
AS_Tasmanian_Ssa03_sdY      --------------------------------------------------
AS_European_sdY             --------------------------------------------------
AS_Tasmanian_Ssa06_sdY      --------------------------------------------------
Chinook_sdY                 TAAATGTTCCATTGCCATTCTGGCTGGCAACATTCTTATCCTTTGCTTGC
RT_sdY                      TAAATGTTCCATTGCCATTCTGGCTGGCAACATTCTTATCCCTTG-TTGC

BT_sdY                      --------------------------------------------------
AS_Tasmanian_Ssa02_sdY      --------------------------------------------------
AS_Tasmanian_Ssa03_sdY      --------------------------------------------------
AS_European_sdY             --------------------------------------------------
AS_Tasmanian_Ssa06_sdY      --------------------------------------------------
Chinook_sdY                 TAGCTAGCCAACTGCGGCTAACTTACAGTTACGTCAAACAGTGCAGTCAG
RT_sdY                      TAGCTAGTCAACTATGGCTAACTTACAGTTACGTCAAACAGTGCAGTGAG

BT_sdY                      --------------------------------------------------
AS_Tasmanian_Ssa02_sdY      --------------------------------------------------
AS_Tasmanian_Ssa03_sdY      --------------------------------------------------
AS_European_sdY             --------------------------------------------------
AS_Tasmanian_Ssa06_sdY      --------------------------------------------------
Chinook_sdY                 CAACAAAGTATATTGCATTTGCATTTGTTTAAGATGTTTTCTAATGACAT
RT_sdY                      CACAAGTATATGCATTTGCATTGTTAG------------ACTTTTCTAAT

BT_sdY                      --------------------------------------------------
AS_Tasmanian_Ssa02_sdY      --------------------------------------------------
AS_Tasmanian_Ssa03_sdY      --------------------------------------------------
AS_European_sdY             --------------------------------------------------
AS_Tasmanian_Ssa06_sdY      --------------------------------------------------
Chinook_sdY                 TTATTTGGATATAACAATGACTTACACAGTTTGGCCTGCCATAGAAAATG
RT_sdY                      TACATTTATTGGATGTAACATGACTCATGGTTGGCCTGCCATAGAAAATG
```

```
BT_sdY                  --------------------------------------------------
AS_Tasmanian_Ssa02_sdY  --------------------------------------------------
AS_Tasmanian_Ssa03_sdY  --------------------------------------------------
AS_European_sdY         --------------------------------------------------
AS_Tasmanian_Ssa06_sdY  --------------------------------------------------
Chinook_sdY             TTCTCTCTCATCAGGACACTGTTTACAGAGGAG-----CAACAACACAGC
RT_sdY                  TTCTCTCTCGTCAGGACACTGTTTACAGAGGAGCTAGTCAACAATACAGC

BT_sdY                  ----------------AAGGGAATCTG-----------------------
AS_Tasmanian_Ssa02_sdY  ----------------AAGGGAATCTG-----------------------
AS_Tasmanian_Ssa03_sdY  ----------------AAGGGAATCTG-----------------------
AS_European_sdY         ----------------AAGGGAATCTG-----------------------
AS_Tasmanian_Ssa06_sdY  ----------------AAGGGAATCTG-----------------------
Chinook_sdY             TAACACAATCACTTCAAACGGAAGCTGAAAAGACTGAAAACTGGCTGCAC
RT_sdY                  TAACACAATCACTTCAAACGGAAGCTGGAAAGACTCAAAACTGGCTGCAC
                                        ** **** ***
BT_sdY                  --------------------------------------------------
AS_Tasmanian_Ssa02_sdY  --------------------------------------------------
AS_Tasmanian_Ssa03_sdY  --------------------------------------------------
AS_European_sdY         --------------------------------------------------
AS_Tasmanian_Ssa06_sdY  --------------------------------------------------
Chinook_sdY             TTTGTTTCCTTTTACCTGTTTTTTATTGATATTTTTTGTATATACAGTAT
RT_sdY                  TTTGTTTCCTTTTACCTGTTTTTTATTGATATTTTTTGTATATAGAGTAT

BT_sdY                  --------------------------------------------------
AS_Tasmanian_Ssa02_sdY  --------------------------------------------------
AS_Tasmanian_Ssa03_sdY  --------------------------------------------------
AS_European_sdY         --------------------------------------------------
AS_Tasmanian_Ssa06_sdY  --------------------------------------------------
Chinook_sdY             CCATAAAAATTATGCTGATTCATGATTTTGACTGGCTGAGAAAAGCTGCC
RT_sdY                  CCATAAAAATTATGCTGATTCGTGATTTTGACTGGCTGAGAAAAGCTGCC
```

```
BT_sdY                     --------------------------------------------------
AS_Tasmanian_Ssa02_sdY     --------------------------------------------------
AS_Tasmanian_Ssa03_sdY     --------------------------------------------------
AS_European_sdY            --------------------------------------------------
AS_Tasmanian_Ssa06_sdY     --------------------------------------------------
Chinook_sdY                AGCCTATCTGTCTCTTCCCGACTCCCGACACATTCGTTACTATGGGACAG
RT_sdY                     TGCCTATCTGTCTCTTCCCGACTCCCGACACATTCGTTACTATGGGACAG

BT_sdY                     --------------------------------------------------
AS_Tasmanian_Ssa02_sdY     --------------------------------------------------
AS_Tasmanian_Ssa03_sdY     --------------------------------------------------
AS_European_sdY            --------------------------------------------------
AS_Tasmanian_Ssa06_sdY     --------------------------------------------------
Chinook_sdY                CTGGAGAAATAATTTGAATTTTGAAACAATGTTGCAATATATCAGAGAGA
RT_sdY                     CTAGAGAAATAATTGGAATTTTGAAACAATGTTCCAATATATCAGAGAGA

BT_sdY                     --------------------------------------------------
AS_Tasmanian_Ssa02_sdY     --------------------------------------------------
AS_Tasmanian_Ssa03_sdY     --------------------------------------------------
AS_European_sdY            --------------------------------------------------
AS_Tasmanian_Ssa06_sdY     --------------------------------------------------
Chinook_sdY                CAGATAGTAAGGGTTATACAAATCTCCGCTGTTTAAAACTAAATGTTAGT
RT_sdY                     CAGATAGTAAGGTTTATACAAATCTCCGCTGTTTAAAACTAAA-------

BT_sdY                     --------------------------------------------------
AS_Tasmanian_Ssa02_sdY     --------------------------------------------------
AS_Tasmanian_Ssa03_sdY     --------------------------------------------------
AS_European_sdY            --------------------------------------------------
AS_Tasmanian_Ssa06_sdY     --------------------------------------------------
Chinook_sdY                CTAAATGGAATGTGAGATAATGTCTAGATGCTTTTAATATTGGAGGTCAA
RT_sdY                     --------------------------------------------------
```

```
BT_sdY                    --------------------------------------------------
AS_Tasmanian_Ssa02_sdY    --------------------------------------------------
AS_Tasmanian_Ssa03_sdY    --------------------------------------------------
AS_European_sdY           --------------------------------------------------
AS_Tasmanian_Ssa06_sdY    --------------------------------------------------
Chinook_sdY               GTTTATACATTGCCTGGCTGGGCTGATGAGACAGTGGATTGTGCTGTCAG
RT_sdY                    -TTTATACATTGCCTGGCTGGGCTGATGAGACAGTGGATTGTGCTGTCAG

BT_sdY                    --------------------------------------------------
AS_Tasmanian_Ssa02_sdY    --------------------------------------------------
AS_Tasmanian_Ssa03_sdY    --------------------------------------------------
AS_European_sdY           --------------------------------------------------
AS_Tasmanian_Ssa06_sdY    --------------------------------------------------
Chinook_sdY               ATGGAACAGAGTAAATAGGCATTTTAACAACATAGATTTAGCTGGTAGTA
RT_sdY                    ATGGAGCAGAGTAAATAGGCATTTTACATAGATAGATTTAGCTGGTAGTA

BT_sdY                    --------------------------------------------------
AS_Tasmanian_Ssa02_sdY    --------------------------------------------------
AS_Tasmanian_Ssa03_sdY    --------------------------------------------------
AS_European_sdY           --------------------------------------------------
AS_Tasmanian_Ssa06_sdY    --------------------------------------------------
Chinook_sdY               ACTTGTGGAATAGATACTGGCTTGAATGCATTTTTAACCAATCAGCATTC
RT_sdY                    ACTTGTGGAATAGATACTGGCTTGAATGCATTTTTAACCCATCAGCATTC

BT_sdY                    --------------------------------------------------
AS_Tasmanian_Ssa02_sdY    --------------------------------------------------
AS_Tasmanian_Ssa03_sdY    --------------------------------------------------
AS_European_sdY           --------------------------------------------------
AS_Tasmanian_Ssa06_sdY    --------------------------------------------------
Chinook_sdY               ATGATTACACACACACGTTGTATAAAACGTATATTGCCAACGATAATGTT
RT_sdY                    ATGATTACACCCACACGTTGTATAAAACGTATATTGCCAACTATAATGTT
```

```
BT_sdY                      ----TTACGGGTAAT------TCACAACAGATTCCACGAAACACTAATTG
AS_Tasmanian_Ssa02_sdY      ----TTACGGGTAAT------TCACAACAGATTTCACGAAACACTAATTG
AS_Tasmanian_Ssa03_sdY      ----TTACGGGTAAT------TCACAACAGATTTCACGAAACACTAATTG
AS_European_sdY             ----TTACGGGTAAT------TCACAACAGATTTCACGAAACACTAATTG
AS_Tasmanian_Ssa06_sdY      ----TTACGGGTAAT------TCACAACAGATTTCACGAAACACTAATTG
Chinook_sdY                 GATATTATGGTTAGTCAGTCCTCACAACAGGTTTCACAAAACACTAAGTG
RT_sdY                      GATATTATGGTTAGTCAGTCCTCACAACAGGTTTCACAAAACACTAAGTG
                                *** ** ** *      ********* ** *** ********* **

BT_sdY                      TGTGCCCTCAGGCCCCTACTCCGCTACCACGTAACTACAACACAAAATCA
AS_Tasmanian_Ssa02_sdY      TGTGCCCTCAGACCCTTACTCCGCTACCACGTAACTACAACACAAAATCA
AS_Tasmanian_Ssa03_sdY      TGTGCCCTCAGACCCTTACTCCGCTACCACGTAACTACAACACAAAATCA
AS_European_sdY             TGTGCCCTCAGACCCTTACTCCGCTACCACGTAACTACAACACAAAATCA
AS_Tasmanian_Ssa06_sdY      TGTGCCCTCAGACCCTTACTCCGCTACCACGTAACTACAACACAAAATCA
Chinook_sdY                 TGTGCCCTCAGACCCCTACTCCACTACCCTATATCTACAACACAAAATSC
RT_sdY                      TGTGCCCTCAGACCCCTACTCCACTACCCTATATCTACAACACAAAATCC
                            *********** *** ****** *****   ** **************

BT_sdY                      ATGTGTAAATTTTTGTATTGTGCGTATGTTATCTTGTGTGTGTATGCGTG
AS_Tasmanian_Ssa02_sdY      ATGTGTACATTTTTGTATTGTGCGTATGTTATCTTGTGTGTGTATGCATG
AS_Tasmanian_Ssa03_sdY      ATGTGTACATTTTTGTATTGTGCGTATGTTATCTTGTGTGTGTATGCATG
AS_European_sdY             ATGTGTACATTTTTGTATTGTGCGTATGTTATCTTGTGTGTGTATGCATG
AS_Tasmanian_Ssa06_sdY      ATGTGTACATTTTTGTATTGTGCGTATGTTATCTTGTGTGTGTATGCATG
Chinook_sdY                 ATGTGTACGTGTGTGTATTGTGAGTATGTTATCGTGTGCGTTTATGCATG
RT_sdY                      ATGTGTACGTGTGTGTATTGTGAGTATGTTATTGTGTGCGTTTATGCATG
                            *******  * * ********* *********  **** ** ***** **
```

```
BT_sdY                      TGTCGGTGCCTATGATTGTGTTGCTTCACAGACCCCGCTGTTCCATAATG
AS_Tasmanian_Ssa02_sdY      TGTCGGTGCCTATGATTGTGTTGCTTCACAGACCCCGCTGTTCCATAATG
AS_Tasmanian_Ssa03_sdY      TGTCGGTGCCTATGATTGTGTTGCTTCACAGACCCCGCTGTTCCATAATG
AS_European_sdY             TGTCGGTGCCTATGATTGTGTTGCTTCACAGACCCCGCTGTTCCATAATG
AS_Tasmanian_Ssa06_sdY      TGTCGGTGCCTATGATTGTGTTGCTTCACAGACCCCGCTGTTCCATAATG
Chinook_sdY                 TGTCAGTGCCTATSTTTGTGTTGCTTCACAGTCCCCGCTGTTCCATAAGG
RT_sdY                      TGTCAGTACCTATGTTTGTGTTGCTTCACAGTCCCCGCTGTTCCATAAGG
                            *** ** *****  **************** **************** *

BT_sdY                      TGTATTTTT------------------ATCTGATTTTATTGA-TGCATCA
AS_Tasmanian_Ssa02_sdY      TGTATTTTT------------------ATCTGATTTTATTGC-TGCATCA
AS_Tasmanian_Ssa03_sdY      TGTATTTTT------------------ATCTGATTTTATTGC-TGCATCA
AS_European_sdY             TGTATTTTT------------------ATCTGATTTTATTGC-TGCATCA
AS_Tasmanian_Ssa06_sdY      TGTATTTTT------------------ATCTGATTTTATTGC-TGCATCA
Chinook_sdY                 TGTATTTTTTAAATCTTTTTTTTTT---ATCTGATTCT--TGCATTTATCA
RT_sdY                      TGTATTTTTTTATCTGTTTTTTTTTAAATCTGATTCTACTGCTTGCATCA
                            *********                  ******** *  **  *  ****

BT_sdY                      GTTACC-------TGATGTGGAATAGTGTTCCATGTAGTCATGGCTCTAT
AS_Tasmanian_Ssa02_sdY      GTTACC-------TGATGTGGAATAGAGTTCCATGTAGTCATGGCTCTAT
AS_Tasmanian_Ssa03_sdY      GTTACC-------TGATGTGGAATAGAGTCCCATGTAGTCATGGCTCTGT
AS_European_sdY             GTTACC-------TGATGTGGAATAGAGTTCCATGTAGTCATGGCTCTAT
AS_Tasmanian_Ssa06_sdY      GTTACC-------TGATGTGGAATAGAGTTCCATGTAGTCATGGCTCTAT
Chinook_sdY                 TTTACCACACATTTTATGTGGAATAGAGTTCCATGTAGTCATGGCTCTAT
RT_sdY                      TTTACC-------TGATGTGGAATAAAGTTCCATGTAGCCATGGCTCTAT
                             *****       * **********  ** ******** ********* *
```

K. P. Lubieniecki *et al.*

```
BT_sdY                    GTAGTACTGTGCTCCTCCCATAG-----TCTGTTCTGGACTTGGGGACTG
AS_Tasmanian_Ssa02_sdY    ATAGTACTGTGCTCCTCCCATAA-----TCTGTTCTGGACTTGAGGACTG
AS_Tasmanian_Ssa03_sdY    ATAGTACTGTGCTCCTCCCATAA-----TCTGTTCTGGACTTGAGGACTG
AS_European_sdY           ATAGTACTGTGCTCCTCCCATAA-----TCTGTTCTGGACTTGAGGACTG
AS_Tasmanian_Ssa06_sdY    ATAGTACTGTGCTCCTCCCATA------TCTGTTCTGGACTTGAGGACTG
Chinook_sdY               GTAGTAATGATTGCCTCTCATGGTCTGTTCTGTTCTGGACTTGGGGACTG
RT_sdY                    GTAGTAATGATCGCCTCCCATGG-----TCTGTTCTGGACTTGGGGACTG
                           ***** **    **** ***       *************** ******

BT_sdY                    TGAAGAGACCTCTGGTGACATGTCTTGTGGGGTATGGATGGGTGTCTGAG
AS_Tasmanian_Ssa02_sdY    TGAAGAGACCTCTGGTGGCATGTCTTGTGGGGTA----------------
AS_Tasmanian_Ssa03_sdY    TGAAGAGACCTCTGGTGGCATGTCTTGTGGGGTA----------------
AS_European_sdY           TGAAGAGACCTCTGGTGGCATGTCTTGTGGGGTA----------------
AS_Tasmanian_Ssa06_sdY    TGAAGAGACCTCTGGTGGCATGTCTTGTGGGGTA----------------
Chinook_sdY               TGAAGAGACCTCTGGTGGCATGTTTTG-----------------------
RT_sdY                    TGAAGAGACCTCTGGTGGCATGTTTTG-----------------------
                          ***************** ***** ***

BT_sdY                    CTGTGAGCCAGTAGTTTAAACAGACCTCTGGTGGCATGTCTTGTGGGGTA
AS_Tasmanian_Ssa02_sdY    --------------------------------------------------
AS_Tasmanian_Ssa03_sdY    --------------------------------------------------
AS_European_sdY           --------------------------------------------------
AS_Tasmanian_Ssa06_sdY    --------------------------------------------------
Chinook_sdY               --------------------------------------------------
RT_sdY                    --------------------------------------------------
```

```
BT_sdY                      TGGATGGGTGTCTGAGCTGTGAGCCAGTAGTTTAAACAGACCTCTGGTGA
AS_Tasmanian_Ssa02_sdY      --------------------------------------------------
AS_Tasmanian_Ssa03_sdY      --------------------------------------------------
AS_European_sdY             --------------------------------------------------
AS_Tasmanian_Ssa06_sdY      --------------------------------------------------
Chinook_sdY                 --------------------------------------------------
RT_sdY                      --------------------------------------------------

BT_sdY                      CATGTCTTGTGGGGTATGGATGGGTGTCTGAGCTGTGTGCCAGTAGTTCA
AS_Tasmanian_Ssa02_sdY      ---------TGGGGTATGCATGGGTGTCCGAGCTGTGTGCCAGTAGTTCA
AS_Tasmanian_Ssa03_sdY      ---------TGGGGTATGCACGGGTGTCCGAGCTGTGTGCCAGTAGTTCA
AS_European_sdY             ---------TGGGGTATGCATGGGTGTCCGAGCTGTGTGCCAGTAGTTCA
AS_Tasmanian_Ssa06_sdY      ---------TGGGGTATGCGTGGGTGTCCGAGCTGTGTGCCAGTAGTTCA
Chinook_sdY                 ---------TGAGTTTTGCATGGGTGTCCACGCTGTGTGCCAGTTGTTCA
RT_sdY                      ---------TGAGTTTTGCAT--GTGTCCACGCTGTGTGCCAGTTGTTCA
                                     ** * * **     *****   ************* *****

BT_sdY                      AACAGACAGTTCAGTACATTCAACATGTCAGTACATTTCACAAATACAAG
AS_Tasmanian_Ssa02_sdY      AACAGACAATTCAGTACATTCAACATGTCAGTACATTTCACAAATACAAG
AS_Tasmanian_Ssa03_sdY      AACAGACAATTCAGTACATTCAACATGTCAGTACATTTCACAAATACAAG
AS_European_sdY             AACAGACAATTCAGTACATTCAACATGTCAGTACATTTCACAAATACAAG
AS_Tasmanian_Ssa06_sdY      AACAGACAATTCAGTACATTCAACATGTCAGTACATTTCACAAATACAAG
Chinook_sdY                 AACAGACAGCTTGGTACATTCAACATTTCAATACATTTCATAAATAAAAG
RT_sdY                      AACAGACAGCTTGGTACATTCAACATTTCAATACATTTCATAAATACAAG
                            ********  *  ************* *** ********* ***** ***
```

```
BT_sdY                      TAGTGATGAAGTCAATCTCTCCTCCACTTTGAGCCATGAGAGATTTACAT
AS_Tasmanian_Ssa02_sdY      TAGTGATGAAGTCAATCTCTCCTCCACTTTGAGCCATGAGAGATTTACAT
AS_Tasmanian_Ssa03_sdY      TAGTGATGAAGTCAATCTCTCCTCCACTTTGAGCCATGAGAGATTTACAT
AS_European_sdY             TAGTGATGAAGTCAATCTCTCCTCCACTTTGAGCCATGAGAGATTTACAT
AS_Tasmanian_Ssa06_sdY      TAGTGATGAAGTCAATCTCTCCTCCACTTTGAGCCATGAGAGATTTACAT
Chinook_sdY                 TAGCGATGAAGTCAATCTCTCCTCTGCTTTGAGCCTGGAGAGATTGACAT
RT_sdY                      TAGCAATGAAGTCAATCTCTCCTCTGCTTTGAGCCTGGAGAGATTGACAT
                            ***  *******************  *********  ******** ****

BT_sdY                      GCATATTATTAATGTTAGCTCTCCATGTACATTTAAGGGCCAGCCGTGCT
AS_Tasmanian_Ssa02_sdY      GCATATTATTAATGTTAGCTCTC--TCTACATTTAAGG----GCCGTGCT
AS_Tasmanian_Ssa03_sdY      GCATATTATTAATGTTAGCTCTC--TCTACATTTAAGG----GCCGTGCT
AS_European_sdY             GCATATTATTAATGTTAGCTCTC--TCTACATTTAAGG----GCCGTGCT
AS_Tasmanian_Ssa06_sdY      GCATATTATTAATGTTAGCTCTC--TCTACATTTAAGG----GCCGTGCT
Chinook_sdY                 GCACAT-----ATATCAGCT---------GATGTAAGA------AGGGCT
RT_sdY                      GCATAT-----ATATCAGCT---------GATGTAAGA------AGGGCT
                            *** **     ** * ****          ** ****        * ***

BT_sdY                      GCCCTGTTCTAGGCCAGTTGTTATTTTCCCAAGTCCCTCTTTGTGGAGAG
AS_Tasmanian_Ssa02_sdY      GCCCTGTTCTAGGCCAGTTGTTATTTTCCCAAGTCCCTCTTTGTGGAGAG
AS_Tasmanian_Ssa03_sdY      GCCCTGTTCTAGGCCAGTTGTTATTTTCCCAAGTCCCTCTTTGTGGAGAG
AS_European_sdY             GCCCTGTTCTAGGCCAGTTGTTATTTTCCCAAGTCCCTCTTTGTGGAGAG
AS_Tasmanian_Ssa06_sdY      GCCCTGTTCTAGGCCAGTTGTTATTTTCCCAAGTCCCTCTTTGTGGAGAG
Chinook_sdY                 ATATAAATATATTTGATTTGAAATTT-----------------------G
RT_sdY                      ATATAAATACATTTGATTTGAAATTT-----------------------G
                                   *  *    * ***  ****                       *
```

```
BT_sdY                      ATTGACATATTATTAATATTAGGTGACCCCTTTGTTATTGGTTCAATTAC
AS_Tasmanian_Ssa02_sdY      ATTGACATATTATTAT-------------ATTTGTTTTTGGTTCAATTAC
AS_Tasmanian_Ssa03_sdY      ATTGACATATTATTAT-------------ATTTGTTTTTGGTTCAATTAC
AS_European_sdY             ATTGACATATTATTAT-------------ATTTGTTTTTGGTTCAATTAC
AS_Tasmanian_Ssa06_sdY      ATTGACATATTATTAT-------------ATTTGTTTTTGGTTCAATTAC
Chinook_sdY                 ATTAGCATATTATTATTATTCGATGAACACTTTGTTATTGGTTCATTTAC
RT_sdY                      ATTTGCATATTATTATTATTCGATGAACACTTTGTTGTTGGTTCATTTAC
                            ***  **********               ****** ******** ****

BT_sdY                      GGATAATAGCTTAAAGTCCATTTTTCACTAAGGGCTCGATTCAATTTGT-
AS_Tasmanian_Ssa02_sdY      GGATAATAGCTTAAAGTCCATTTTTCACTAAGGGCTCGATTCAATTTGT-
AS_Tasmanian_Ssa03_sdY      GGATAATAGCTTAAAGTCCATTTTTCACTAAGGGCTCGATTCAATTTGT-
AS_European_sdY             GGATAATAGCTTAAAGTCCATTTTTCACTAAGGGCTCGATTCAATTTGT-
AS_Tasmanian_Ssa06_sdY      GGATAATAGCTTAAAGTCCATTTTTCACTAAGGGCTCGATTCAATTTGT-
Chinook_sdY                 AGATAATTGCTTAAATTCAATGTTTCACTAAGGGC-CGATTCAATCTGTA
RT_sdY                      AGATAATTGCTTGAATTCAATGTTTCACTAAGGGCTCGATTCAATCTGTA
                             ****** **** ** ** ** ************* ********* ***

BT_sdY                      -------AGTTCGGGGCTAATGCTGAATTACATTTAAAGGTAA-ATTCCG
AS_Tasmanian_Ssa02_sdY      -------AGTTCGGGGCTAATGCTGAATTACATTTAAAGGTAA-ATTCTG
AS_Tasmanian_Ssa03_sdY      -------AGTTCGGGGCTAATGCTGAATTACATTTAAAGGTAA-ATTCTG
AS_European_sdY             -------AGTTCGGGGCTAATGCTGAATTACATTTAAAGGTAA-ATTCTG
AS_Tasmanian_Ssa06_sdY      -------AGTTCGGGGCTAATGCTGAATTACATTTAAAGGTAA-ATTCTG
Chinook_sdY                 TTGCGGAAGTTGAAGGCTAATGCTTGATTACATTTGAAGGTAA-TTTCCG
RT_sdY                      TTGCGGAAGTTCAAGGCTAATGCTTGATTACATTTGAAGGTAATTTTCCG
                                   ****   **********  ********* *******  *** *
```

```
BT_sdY                    ATTTAGCTGACATACTGTTTGCAGCATATACTGTAAATGCA------ACA
AS_Tasmanian_Ssa02_sdY    ATTTAGCTGACATACTGTTTGCAGCGTATACTGTAAATGCA------ACA
AS_Tasmanian_Ssa03_sdY    ATTTAGCTGACATACTGTTTGCAGCGTATACTGTAAATGCA------ACA
AS_European_sdY           ATTTAGCTGACATACTGTTTGCAGCGTATACTGTAAATGCA------ACA
AS_Tasmanian_Ssa06_sdY    ATTTAGCTGACATACTGTTTGCAGCGTATACTGTAAATGCA------ACA
Chinook_sdY               ATTGAGCCGACATACTGTTTGCAGCATATAATGTAAATGCAGTCTCCACA
RT_sdY                    ATTGAGCCGACATACTGTTTGCAGCATATACTGCAAATGCAGTCTCCACA
                          *** *** ***************** **** ** *******      ***

BT_sdY                    AAATGCAGGATCATTGCCTTAAATGTATATTGCGCATTAGAACTGCTTTG
AS_Tasmanian_Ssa02_sdY    AAATGCAGGATCATTGCC------------------TTAGCACTGTTTTG
AS_Tasmanian_Ssa03_sdY    AAATGCAGGATCATTGCC------------------TTAGCACTGTTTTG
AS_European_sdY           AAATGCAGGATCATTGCC------------------TTAGCACTGTTTTG
AS_Tasmanian_Ssa06_sdY    AAATGCAGGATCATTGCC------------------TTAGCACTGTTTTG
Chinook_sdY               AAATGCAGGAGCATTTCCTTAAATGTATTTTTGTCATTAGCACTGCATTG
RT_sdY                    AAATGCAGGAGCATTTCCTTAAATGTATTTTTGTCATTAGCACTGCATTG
                          ********** **** **                  **** ****   ***

BT_sdY                    ATGTTAATGTGAGCATCCCTCTCTTGTCCTACTGAATCTGGCTGATAATA
AS_Tasmanian_Ssa02_sdY    ATGTTAATGTGAGCATCCCTCTCTTGTCCTACTGAATCAGGCTGATAATA
AS_Tasmanian_Ssa03_sdY    ATGTTAATGTGAGCATCCCTCTCTTGTCCTACTGAATCTGGCTGATAATA
AS_European_sdY           ATGTTAATGTGAGCATCCCTCTCTTGTCCTACTGAATCTGGCTGATAATA
AS_Tasmanian_Ssa06_sdY    ATGTTAATGTGAGCATCCCTCTCTTGTCCTACTGAATCTGGCTGATAATA
Chinook_sdY               ATGTTATTGTGAGCATCACTCTCTTGTCCTACTGAATTTGTCTGATTATA
RT_sdY                    ATGTTAATGTGAGCATCACTCTCTCGTACTACTGAATTTGTCTGATTATA
                          ****** ********** ****** ** *********  * ***** ***
```

```
BT_sdY                      TTACTGATTGTATCACTGATGAAATTACAGTAGTGTGTTTATCATGTGAT
AS_Tasmanian_Ssa02_sdY      TTACTGATTGTATCACTGATGAAATTACAGTAGTGTGTTTATCATGTGAT
AS_Tasmanian_Ssa03_sdY      TTACTGATTGTATCACTGATGAAATTACAGTAGTGTGTTTATCATGTGAT
AS_European_sdY             TTACTGATTGTATCACTGATGAAATTACAGTAGTGTGTTTATCATGTGAT
AS_Tasmanian_Ssa06_sdY      TTACTGATTGTATCACTGATGAAATTACAGTAGTGTGTTTATCATGTGAT
Chinook_sdY                 TTACTGATTGTATCACTGATGATATTGCAGCAGTGTGTATGTCATGTGAT
RT_sdY                      TTACTGATTGTATCACTGATGATATTGCAGCGGTGTGTTTGTCATGTGAT
                            ********************** *** ***  ****** * *********

                                    |          exon3
                                                      |---------Exon 3F--------
BT_sdY                      GGATTACTGACTTTGTGTGTCCTTAG|AGTTGGAACGCTTCAGCCGAGCAG
AS_Tasmanian_Ssa02_sdY      GGATTACTGACTTTGTGTGTCCTTAG|AGTTGGAACGCTTCAGCAGAGCAG
AS_Tasmanian_Ssa03_sdY      GGATTACTGACTTTGTGTGTCCTTAG|AGTTGGAACGCTTCAGCAGAGCAG
AS_European_sdY             GGATTACTGACTTTGTGTGTCCTTAG|AGTTGGAACGCTTCAGCAGAGCAG
AS_Tasmanian_Ssa06_sdY      GGATTACTGACTTTGTGTGTCCTTAG|AGTTGGAACGCTTCAGCAGAGCAG
Chinook_sdY                 GTATTACTGACTCTCTGTGTCCATAG|AGTTGGAACGCTTCGCCAGAGCAG
RT_sdY                      GTATTACTGACTCTGTGTGTCCTTAG|AGTTGGACCGCTTCGCCAGAGCAG
                            * ********** * ******* ***|******* ******  * ******

                               exon3
                            --->                                          <-------
BT_sdY                      ATGGCTTCCAACCGCAAATTGGGTTCAGCCTATGGTTCGGACAAGACTCA
AS_Tasmanian_Ssa02_sdY      ATGGCTTCCAACCGCAAATTGGGTTCAGCCTATGGTTCGGACAAGACTCA
AS_Tasmanian_Ssa03_sdY      ATGGCTTCCAACCGCAAATTGGGTTCAGCCTATGGTTCGGACAAGACTCA
AS_European_sdY             ATGGCTTCCAACCGCAAATTGGGTTCAGCCTATGGTTCGGACAAGACTCA
AS_Tasmanian_Ssa06_sdY      ATGGCTTCCAACCGCAAATTGGGTTCAGCCTATGGTTCGGACAAGACTCA
Chinook_sdY                 ATGGCTCCCAACCTCAATGTGGGTTCAGCCTATGGTTTGGACAAGACTCA
RT_sdY                      ATGGCTCCCAACCTCAATGTGGGTTCAGCCTATGGTTTGGACAAGACTCA
                            ****** ****** ***  ****************** ************
```

```
                          exon3               |
                            ---Exon 3R---------        |
BT_sdY                      TCACTCAGTGCACCAATCTCTATATCG|GTACAGATTATTATTATTATATA
AS_Tasmanian_Ssa02_sdY      TCACTCAGTGCACCAATCTCTATATCG|GTACAGATTATTATTATTATATA
AS_Tasmanian_Ssa03_sdY      TCACTCAGTGCACCAATCTCTATATCG|GTACAGATTATTATTATTATATA
AS_European_sdY             TCACTCAGTGCACCAATCTCTATATCG|GTACAGATTATTATTATTATATA
AS_Tasmanian_Ssa06_sdY      TCACTCAGTGCACCAATCTCTATATCG|GTACAGATTATTATTATTATATA
Chinook_sdY                 TCACTCAGTGCACCAAACTTTATATCG|GTACAGATT----CTCTTAC---
RT_sdY                      TCACTCAGTGCACCAATCTTTATATCG|GTACAGATT----CTCTTAC---
                            **************** ** *******|*********     * ***

BT_sdY                      TAGATTATCTGGTGCCTTGAATACATTAACACAGCGCCGTCCTCAGAAAT
AS_Tasmanian_Ssa02_sdY      TAGATTATCTGGTGCCTTGAATACATTAACACAGTGCCGGCCTCAGAAAT
AS_Tasmanian_Ssa03_sdY      TAGATTATCTGGTGCCTTGAATACATTAACACAGTGCCGGCCTCAGAAAT
AS_European_sdY             TAGATTATCTGGTGCCTTGAATACATTAACACAGTGCCGGCCTCAGAAAT
AS_Tasmanian_Ssa06_sdY      TAGATTATCTGGTGCCTTGAATACATTAACACAGTGCCGGCCTCAGAAAT
Chinook_sdY                 -------TCTGGGGCATTGTACACATTAACACAGGGCCGACGCTAGAAGG
RT_sdY                      -------TCTGGGGCATTGTACACATTAACACAGGGCCGACGCTAGAAGG
                                   ***** ** *** * ************ **** *   ****

                                           <---AS&BT 10bo gap R--
BT_sdY                      AACCATTGGGAAGGGCAATTGATTTCCATAGGCGGACATGTTTTTTTTCA
AS_Tasmanian_Ssa02_sdY      AACCA--AGGAAGGGCAATTGATTTCCATAGGCGGACATGTTTTTTTTCA
AS_Tasmanian_Ssa03_sdY      AACCATTGGGAAGGGCAATTGATTTCCATAGGCGGACATGTTTTTTTTCA
AS_European_sdY             AACCATTGGGAAGGGCAATTGATTTCCATAGGCGGACATGTTTTTTTTCA
AS_Tasmanian_Ssa06_sdY      AACCA--AGGAAGGGCAATTGATTTCCATAGGCGGACATGTTTTTTTTCA
Chinook_sdY                 AATCATTGAGAAGGGCAATTGATTTCCATAGG-----ATG-TTTTTTTTCA
RT_sdY                      AATCATTGAGAAG-------GATTTCCATAGG-----ATG-TTTTTTTTCA
                            ** **    ****       ************     *** *********
```

```
BT_sdY                      TGGCTTTTATAGTAAAGACATGTCATTTATCTTAACAAATGTATTACCTT
AS_Tasmanian_Ssa02_sdY      TGGCTTTTATAGTAAAGACATGTCATTTATCTTAACAAATGTATTACCTT
AS_Tasmanian_Ssa03_sdY      TGGCTTTTATAGTAAAGACATGTCATTTATCTTAACAAATGTATTACCTT
AS_European_sdY             TGGCTTTTATAGTAAAGACATGTCATTTATCTTAACAAATGTATTACCTT
AS_Tasmanian_Ssa06_sdY      TGGGTTTTATAGTAAAGACATGTCATTTATCTTAACAAATGTATTACCTT
Chinook_sdY                 TGGTTTTTATAGTAAAG--ATGTAATTTATCTTTAAAAATATATTTCCTT
RT_sdY                      TGGTTTTTATAGTAAAG--ATGTAATTTTTCTTTAAAAATATATTCCCTT
                            *** *************  **** **** **** * **** **** ****

                                     -Exon 3-intron-end F2->
BT_sdY                      TTAATGTGGTATGAACACAACCAGTCATGATGTTTTCATCTGATTGTCAA
AS_Tasmanian_Ssa02_sdY      TTAATGTGGTATGAACACAACCAGTCATGATGTTTTCATCTGATTGTCAA
AS_Tasmanian_Ssa03_sdY      TTAATGTGGTATGAACACAACCAGTCATGATGTTTTCATCTGATTGTCAA
AS_European_sdY             TTAATGTGGTATGAACACAACCAGTCATGATGTTTTCATCTGATTGTCAA
AS_Tasmanian_Ssa06_sdY      TTAATGTGGTATGAACACAACCAGTCATGATGTTTTCATCTGATTGTCAA
Chinook_sdY                 TTAATGTGGCATGTACACAACCAGTCATGATGTTTTCATCTGATTGTCAA
RT_sdY                      TTAATGTGGCACGTACACAACCAGTCATGATGTTTTCATCTGATTGTCAA
                            ********* * * ************************************

BT_sdY                      AC------------------------------------------------
AS_Tasmanian_Ssa02_sdY      ACGAATCTTTGTAAGTCGCTCTGGATAAGAGCGTCTGCTAAATGACTTAA
AS_Tasmanian_Ssa03_sdY      ACGAATCTTTGTAAGTCGCTCTGGATAAGAGCGTCTGCTAAATGACTTAA
AS_European_sdY             ACGAATCTTTGTAAGTCGCTCTGGATAAGAGCGTCTGCTAAATGACTTAA
AS_Tasmanian_Ssa06_sdY      ACGAATCTTTGTAAGTCGCTCTGGATAAGAGCGTCTGCTAAATGACTTAA
Chinook_sdY                 AA------------------------------------------------
RT_sdY                      AA------------------------------------------------
                            *
```

```
BT_sdY                      ---------------GAATCACTTCAAAAAGTAGGTTACCTTTGCACGTT
AS_Tasmanian_Ssa02_sdY      ATGTTAAATGTAAATGAATCACTTCAAAAAGTAGGTTACCTTCGCACGTT
AS_Tasmanian_Ssa03_sdY      ATGTTAAATGTAAATGAATCACTTCAAAAAGTAGGTTACCTTCGCACGTT
AS_European_sdY             ATGTTAAATGTAAATGAATCACTTCAAAAAGTAGGTTACCTTCGCACGTT
AS_Tasmanian_Ssa06_sdY      ATGTTAAATGTAAATGAATCACTTCAAAAAGTAGGTTACCTTCGCACGTT
Chinook_sdY                 ---------------AAATAATTTCAAAAAGTATGTTACCTTCGCATGTT
RT_sdY                      ---------------AAATAATTTCAAAAAGTAGGTTACCTTCGCATGTT
                                            *** * *********** ******** *** ***

BT_sdY                      CGTCCAAAATAATTCTAGCATCCGAAGACTGCACTGTGTACCCGCCAACT
AS_Tasmanian_Ssa02_sdY      CGTCCAAAATAATTCTAGCATCCGAAGACTGCACTGTGTACCCGCCAACT
AS_Tasmanian_Ssa03_sdY      CGTCCAAAATAATTCTAGCATCCGAAGACTGCACTGTGTACCCGCCAACT
AS_European_sdY             CGTCCAAAATAATTCTAGCATCCGAAGACTGCACTGTGTACCCGCCAACT
AS_Tasmanian_Ssa06_sdY      CGTCCAAAATAATTCTAGCATCCGAAGACTGCACTGTGTACCCGCCAACT
Chinook_sdY                 CATCCAAAATAATTCTAGCATTTGAACTGGGCACTGTGGACCTGCCAATT
RT_sdY                      CATCCAAAATAATTCTAGCATTTGAAC-TGGCACTGTGGACCTGCCAATT
                            * *******************  ***    ******** *** ***** *

BT_sdY                      TTTGTTCAATTTGCAAGTGGT--------------TGGAGAAAACATCAG
AS_Tasmanian_Ssa02_sdY      TTTGTTCAATTTGCAAGTGGT--------------TGGAGAAAACATCTG
AS_Tasmanian_Ssa03_sdY      TTTGTTCAATTTGCAAGTGGT--------------TGGAGAAAACATCAG
AS_European_sdY             TTTGTTCAATTTGCAAGTGGT--------------TGGAGAAAACATCAG
AS_Tasmanian_Ssa06_sdY      TTTGTTCAATTTGCAAGTGGT--------------TGGAGAAAACATCTG
Chinook_sdY                 TTCTTTCAATTTGCACGTGGTTGAAACTATCTTACTGGAGAAAACATCAG
RT_sdY                      TTCTTTCAATTTGCAAGTGGCTGAAACTATCTTACTGGAGAAAACATCAG
                            **  *********** ****               ************* *
```

```
BT_sdY                   AGTGAGCAAAACAGCGTCTTG-----------------------------
AS_Tasmanian_Ssa02_sdY   AGCGAGCAAAAGAGCGTCTTG-----------------------------
AS_Tasmanian_Ssa03_sdY   AGCGAGCAAAAGAGCGTCTTG-----------------------------
AS_European_sdY          AGCGAGCAAAAGAGCGTCTTG-----------------------------
AS_Tasmanian_Ssa06_sdY   AGCGAGCAAAAGAGCGTCTTG-----------------------------
Chinook_sdY              ATAGAGCAAAACAGTGCCTTACTATATGTAGCCCATGTTTCTGATGCTAT
RT_sdY                   ATAGAGCAAAACAGTGCCTTACTATATGTAGCCCATGTTTCTGATGCTAT
                         *  ******** ** * ***

BT_sdY                   ---------------------------GCTATTGTGGGCATGATAGCATC
AS_Tasmanian_Ssa02_sdY   ---------------------------GCTATTGTGGGCTAGACAGCATC
AS_Tasmanian_Ssa03_sdY   ---------------------------GCTATTGTGGGCTAGACAGCATC
AS_European_sdY          ---------------------------GCTATTGTGGGCTAGACAGCATC
AS_Tasmanian_Ssa06_sdY   ---------------------------GCTATTGTGGGCTAGACAGCATC
Chinook_sdY              TTGGCTAAAAATAGTATGACATGTCATACTATTGTGGGCCAGACAGCATC
RT_sdY                   CTGGCTAAAAATAGTATGACATGTCATACTATTGTGGGCCAGACAGCATC
                                                     ***********  ** ******

BT_sdY                   AGA--------CTAGATACAGCAAGA--------CGCTGTTTCGCTCACT
AS_Tasmanian_Ssa02_sdY   AGG--------CTACGTATAGTAAGA--------CGCTGTTTCGCTCACT
AS_Tasmanian_Ssa03_sdY   AGG--------CTACATATAGTAAGA--------CGCTGTTTCGCTCACC
AS_European_sdY          AGG--------CTACATATAGTAAGA--------CGCTGTTTCGCTCACT
AS_Tasmanian_Ssa06_sdY   AGG--------CTACATATAGTAAGA--------CGCTGTTTCGCTCACT
Chinook_sdY              AGACACATGGCCTACACACTCTGAGACAGAGGGGCGCTGTCTGGCTCACT
RT_sdY                   AGACACATGGCCTACACACTCTGAGACAGAGGGGCGCTGTCTGGCTCACT
                         **         ***   *     ***        ****** * ******
```

K. P. Lubieniecki *et al.*

```
BT_sdY                      CGAATACTTTATCTGAGATTTACGTGCTTTTCTGTCGGCACGAGTCTCG-
AS_Tasmanian_Ssa02_sdY      CGAATACTTTATCTGAGATTTACGTGGTTTTCTGTCGGCACGAGTCTCGG
AS_Tasmanian_Ssa03_sdY      CGAATACTTTATCTGAGATTTACGTGGTTTTCTGTCGGCACGAGTCTCGG
AS_European_sdY             CGAATACTTTATCTGAGATTTACGTGGTTTTCTGTCGGCACGAGTCTCGG
AS_Tasmanian_Ssa06_sdY      CGAATACTTTATCTGAGATTTACGTGGTTTTCTGTCGGCACGAGTCTCGG
Chinook_sdY                 CAGATGCTTTATCTGAGATTGATGTGTCTTTCT----------GTCTCGG
RT_sdY                      CGGAAGCTTTATCTGAGATTGATGTGTCTTTCT----------GTCTCGG
                            *  *  ************** * ***  *****          ******

BT_sdY                      --------------------------------------------------
AS_Tasmanian_Ssa02_sdY      TCAAATTTAATTTGGAAGGGTAAGGACGTATGGTGGG------------G
AS_Tasmanian_Ssa03_sdY      TCAAATTTAATTTGGAAGGGTAAGGACGTATGGTGGG------------G
AS_European_sdY             TCAAATTTAATTTGGAAGGGTAAGGACGTATGGTGGG------------G
AS_Tasmanian_Ssa06_sdY      TCAAATTTAATTTGGAAGGGTAAGGACGTATGGTGGG------------G
Chinook_sdY                 TCAAATTTAATTTGGA--GGTACG---GTGGGGTGGGCCCGCCAAAACCA
RT_sdY                      TCAAATTTAATTTGGATGGGCAAG---GTGGGGTGGGCCCACCAAAACCA


BT_sdY                      --------------------------------------------------
AS_Tasmanian_Ssa02_sdY      CGGGCCCTGCATTAACTACAACAGTTATGATAACATTATGTTTTCCTCCA
AS_Tasmanian_Ssa03_sdY      CGGGCCCTGCATTAACTACAACAGTTATGATAACATTATGTTTTCCTCCA
AS_European_sdY             CGGGCCCTGCATTAACTACAACAGTTATGATAACATTATGTTTTCCTCCA
AS_Tasmanian_Ssa06_sdY      CGGGCCCTGCATTAACTACAACAGTTATGATAACATTATGTTTTCCTCCA
Chinook_sdY                 CAGGCCCTGCATTAACTTCAACAGTTATGATAACATTACATTTTCCTCTA
RT_sdY                      CAGGCCCTGCATTAACTTCAACAGTTATGATAACATTACATTTTCCTCTA
```

```
BT_sdY                    --------------------------------------------------
AS_Tasmanian_Ssa02_sdY    TTTTCCTGTAATTTTC----------------------------------
AS_Tasmanian_Ssa03_sdY    TTTTCCTGTAATTTTC----------------------------------
AS_European_sdY           TTTTCCTGTAATTTTC----------------------------------
AS_Tasmanian_Ssa06_sdY    TTTTCCTGTAATTTTC----------------------------------
Chinook_sdY               TATTACTGTAATTTTCCACCATACAGTGCATTCGGAAAATATTCAGACCC
RT_sdY                    TATTACTGTAATTTTCCACCATACAGTGCATTCGGAAAGTATTCAAACCC


BT_sdY                    --------------------------------------------------
AS_Tasmanian_Ssa02_sdY    -------------------------GTTACAGCCTCATTCTAAAATTGAT
AS_Tasmanian_Ssa03_sdY    -------------------------GTTACAGCCTTATTCTAAAATTGAT
AS_European_sdY           -------------------------GTTACAGCCTTATTCTAAAATTGAT
AS_Tasmanian_Ssa06_sdY    -------------------------GTTACAGCCTTATTCTAAAATTGAT
Chinook_sdY               ATTCCCTTTTCCCACATTTTGTTATGCTACAGCCTTATTCTTATATTGAT
RT_sdY                    ATTCCCTTTTCCCACATTTTGTTATGCTACAGCCTTATTCTAATATTGAT


BT_sdY                    --------------------------------------------------
AS_Tasmanian_Ssa02_sdY    TAAATAAATGTTTTTCCTCATCAATCTACACACAATACCTTATAATGGTA
AS_Tasmanian_Ssa03_sdY    TAAATAAATGTTTTTCCTCATCAATCTACACACAATACCTTATAATGGTA
AS_European_sdY           TAAATAAATGTTTTTCCTCATCAATCTACACACAATACCTTATAATGGTA
AS_Tasmanian_Ssa06_sdY    TAAATAAATGTTTTTCCTCATCAATCTACACACAATACCTTATAATGGTA
Chinook_sdY               TAAAT---CGGTTTTCCTCATCAATCTACACACAATACCCCATAATGACA
RT_sdY                    TAAAT---CGTTTTTCCTCATCAATCTACACACAATACCCCATAATGACA
```

```
BT_sdY                   --------------------------------------------------
AS_Tasmanian_Ssa02_sdY   TATATAACAGATTTAAAGATTTTTTACAAATGT-------ATAAAAAAAT
AS_Tasmanian_Ssa03_sdY   TATATAACAGATTTAAAGATTTTTTACAAATGT-------ATAAAAAAAT
AS_European_sdY          TATATAACAGATTTAAAGATTTTTTACAAATGT-------ATAAAAAAAT
AS_Tasmanian_Ssa06_sdY   TATATAACAGATTTAAAGATTTTTTACAAATGT-------ATAAAAAAAT
Chinook_sdY              --------AGGTGAAAACAGGTTTTAAGAAAGTTTTGCTGATTAAAAAAC
RT_sdY                   --------AGGTGAAAACAGGTTTTAAGAAAGTTTTGCTGATTAAAAAAC


BT_sdY                   --------------------------------------------------
AS_Tasmanian_Ssa02_sdY   GT----------------TTATTTACATAAGTATTTAGACCCTTTGCTAT
AS_Tasmanian_Ssa03_sdY   GT----------------TTATTTACATAAGTATTTAGACCCTTTGCTAT
AS_European_sdY          GT----------------TTATTTACATAAGTATTTAGACCCTTTGCTAT
AS_Tasmanian_Ssa06_sdY   GT----------------TTATTTACATAAGTATTTAGACCCTTTGCTAT
Chinook_sdY              ATCAACAACCTATATATCTTATTTACATAAGATTTCAGACCCTTTGCTAT
RT_sdY                   ATCAACAACCTATACATCTTATTTACATAAGATTTCAGACCCTTTGCTAT


BT_sdY                   --------------------------------------------------
AS_Tasmanian_Ssa02_sdY   GAAACTCGAAATTAAGCTCAGCTGCATCCGGTTTC--CATTGATAATATT
AS_Tasmanian_Ssa03_sdY   GAAACTCGAAATTAAGCTCAGCTGCATCCGGTTTC--CATTGATAATATT
AS_European_sdY          GAAACTCGAAATTAAGCTCAGCTGCATCCGGTTTC--CATTGATAATATT
AS_Tasmanian_Ssa06_sdY   GAAACTCGAAATTAAGCTCAGCTGCATCCGGTTTC--CATTGATAATATT
Chinook_sdY              GAGAATCAAAATTAATATCAGCAGAATCCGGTTTCTGCTTTGATAATATT
RT_sdY                   GAGAATCAAAATTAATCTCAGCAGAATCCGGTTTC--CATTGATAATATT
```

```
                                                                   ---Exon 3-4
BT_sdY                      --------------------------------------------------
AS_Tasmanian_Ssa02_sdY      TGAGATGTTTCTACAACTTGATTGGAGTCCACCTCTGGTAAATTCAATTT
AS_Tasmanian_Ssa03_sdY      TGAGATGTTTCTACAACTTGATTGGAGTCCACCTCTGGTAAATTCAATTT
AS_European_sdY             TGAGATGTTTCTACAACTTGATTGGAGTCCACCTCTGGTAAATTCAATTT
AS_Tasmanian_Ssa06_sdY      TGAGATGTTTCTACAACTTGATTGGAGTCCACCTCTGGTAAATTCAATTT
Chinook_sdY                 TTAGATGTTTCTACAACTTGAGTGGAGTCCACCTGTGGTGGATTCAATTC
RT_sdY                      TTAGATGTTTCTACAACTTGAGTGGAGTCCACCTGTGGTAGATTAAATTC


                            F2a------->
BT_sdY                      --------------------------------------------------
AS_Tasmanian_Ssa02_sdY      A-TGGGACATGATTTGGAAAGGCACACTCCTGTCTATATAAGGTCCCACA
AS_Tasmanian_Ssa03_sdY      A-TGGGACATGATTTGGGAAGGCACACTCCTGTCTATATAAGGTCCCACA
AS_European_sdY             A-TGGGACATGATTTGGAAAGGCACACTCCTGTCTATATAAGGTCCCACA
AS_Tasmanian_Ssa06_sdY      A-TGGGACATGATTTGGAAAGGCACACTCCTGTCTATATAAGGTCCCACA
Chinook_sdY                 ATTGGGGCATGATATTGAAAGGCACACACCTGTCTATACAAGGTCCCACA
RT_sdY                      ATTGGGGCATGATATTGAAAGGCACACACCTGTCTATACAAGGTCCCACA


BT_sdY                      --------------------------------------------------
AS_Tasmanian_Ssa02_sdY      GTTGACAGTGCATTTCAGAGCAAAAACCAAGTCATGAGGGCAAAGGAATT
AS_Tasmanian_Ssa03_sdY      GTTGACAGTGCATTTCAGAGCAAAAACCAAGTCATGAGGGCAAAGGAATT
AS_European_sdY             GTTGACAGTGCATTTCAGAGCAAAAACCAAGTCATGAGGGCAAAGGAATT
AS_Tasmanian_Ssa06_sdY      GTTGACAGTGCATTTCAGAGCAAAAACCAAGTCATGAGGGCAAAGGAATT
Chinook_sdY                 GTTGACAGTGCATGTCAGGGCAAAAACAAAGCCATGAGATTGAAAGAATT
RT_sdY                      GTTGACAGTGCATGTCAGGGCAAAAACCAAGCCATGAGATTGAAAGAATT
```

```
BT_sdY                    --------------------------------------------------
AS_Tasmanian_Ssa02_sdY    ATCCGTAGGGCTCCAAAACAGGATTGTGTATAGGCACAGATCAGGGGAAG
AS_Tasmanian_Ssa03_sdY    ATCCGTAGAGCTCCAAAACAGGATTGTGTATAGGCACAGATCAGGGGAAG
AS_European_sdY           ATCCGTAGAGCTCCAAAACAGGATTGTGTATAGGCACAGATCAGGGGAAG
AS_Tasmanian_Ssa06_sdY    ATCCGTAGAGCTCCAAAACAGGATTGTGTATAGGCACAGATCAGGGGAAG
Chinook_sdY               ATCCG----------AAACAAGATTGTGTTGAGGCACAGATC-TGGGAAG
RT_sdY                    ATCCG----------AAACAAGATTGTGTTGAGGCACAGATC-TGGGAAG


BT_sdY                    --------------------------------------------------
AS_Tasmanian_Ssa02_sdY    GGTACCAAAACATTTCTGAAGCATTGAAGGTCCCCAAGAACACAGTGGTC
AS_Tasmanian_Ssa03_sdY    GGTACCAAAACATTTCTGAAGCATTGAAGGTCCCCAAGAACACAGTGGTC
AS_European_sdY           GGTACCAAAACATTTCTGAAGCATTGAAGGTCCCCAAGAACACAGTGGTC
AS_Tasmanian_Ssa06_sdY    GGTACCAAAACATTTCTGAAGCATTGAAGGTCCCCAAGAACACAGTGGTC
Chinook_sdY               GGTACCAAAACATTTCTGAAGCATTGAAGGTCCCCAAGA--TCAGTGGTC
RT_sdY                    GGTACCAAAACGTTTCTGAA-----------CCCCAAGA--TCAATGGTC


BT_sdY                    --------------------------------------------------
AS_Tasmanian_Ssa02_sdY    TCCATCATTCTTAAATGGAAGAAGTCTGGAAACACCAAGATTCTTCCTAG
AS_Tasmanian_Ssa03_sdY    TCCATCATTCTTAAATGGAAGAAGTTTGGAAACACCAAGATTCTTCCTAG
AS_European_sdY           TCCATCATTCTTAAATGGAAGAAGTTTGGAAACACCAAGATTCTTCCTAG
AS_Tasmanian_Ssa06_sdY    TCCATCATTCTTAAATGGAAGAAGTTTGGAAACACCAAGATTCTTCCTAG
Chinook_sdY               TCCATCATTCTGAAATGGAAGAAGTTTGGAACCACCGAAACTCATCCTAG
RT_sdY                    TCCATCATTCTGAAATGGAAGAAGTTTGGAACCACTGAAACTCATCCTAG
```

```
BT_sdY                      --------------------------------------------------
AS_Tasmanian_Ssa02_sdY      AGCTGGCCACCCG-------------------------------------
AS_Tasmanian_Ssa03_sdY      AGCTGGCCACCCG-------------------------------------
AS_European_sdY             AGCTGGCCACCCG-------------------------------------
AS_Tasmanian_Ssa06_sdY      AGCTGGCCACCCG-------------------------------------
Chinook_sdY                 AGTTGGCCGCCCAGAGCTGGACTCCTGAGTGGAGCAGTGGTCTAAGGCAC
RT_sdY                      AGTTGGCCGCCCA------------------GAGCAGCGGTCTAAGGCAC

BT_sdY                      --------------------------------------------------
AS_Tasmanian_Ssa02_sdY      --------------------------------------------------
AS_Tasmanian_Ssa03_sdY      --------------------------------------------------
AS_European_sdY             --------------------------------------------------
AS_Tasmanian_Ssa06_sdY      --------------------------------------------------
Chinook_sdY                 TGCATCTTAGTGCTAGATTGGTCACTACAGTCCCGGTTTCGATCCCGGGC
RT_sdY                      TGCATCTCAGTGCTAGATTGGTCACTACAGTCCCGGTTTCGATCCCGGGC

BT_sdY                      --------------------------------------------------
AS_Tasmanian_Ssa02_sdY      --------------------------------------------------
AS_Tasmanian_Ssa03_sdY      --------------------------------------------------
AS_European_sdY             --------------------------------------------------
AS_Tasmanian_Ssa06_sdY      --------------------------------------------------
Chinook_sdY                 TGTATCACAACCGGTCGTGATTTGGAGTCCCATAGGGCGCCGTACAATTT
RT_sdY                      TGTATCACAACCGGTCGTGATTTGGAGTCCCATAGGGCGGCGTACAATTT
```

```
BT_sdY                      ----------------------------------------GCCAAACTGA
AS_Tasmanian_Ssa02_sdY      ----------------------------------------GCCAACCTGA
AS_Tasmanian_Ssa03_sdY      ----------------------------------------GCCAACCTGA
AS_European_sdY             ----------------------------------------GCCAACCTGA
AS_Tasmanian_Ssa06_sdY      ----------------------------------------GCCAACCTGA
Chinook_sdY                 GGCCCAGCGTCGTCCGGGTTAGGGGAGGCCGGGGGAGGCCGTCATTGTAA
RT_sdY                      GGCCCAGCGTTGTCCGGGTTAGGGGAGGCCGGGGGAGGCCGTCATTGTAA
                                                                    * **   * *

BT_sdY                      GCA-----------------------------------------------
AS_Tasmanian_Ssa02_sdY      GCA-----------------------------------------------
AS_Tasmanian_Ssa03_sdY      GCA-----------------------------------------------
AS_European_sdY             GCA-----------------------------------------------
AS_Tasmanian_Ssa06_sdY      GCA-----------------------------------------------
Chinook_sdY                 GCAAGAATTTGTTTTTATATGACTTGCCTAGTTAAATAATGGTTAA--AT
RT_sdY                      ACAAGAATTTGTTTTTATATTACTTGCCTAGTTAAATAATGGCTAAATAT
                             **

BT_sdY                      ----------------ATCGGGGGAGAAGGGCCTTGGTCATGGAGGTGAT
AS_Tasmanian_Ssa02_sdY      ----------------ATCGGGGGAGAAGGGCCTTGGTCAGGGAGGTGAT
AS_Tasmanian_Ssa03_sdY      ----------------ATCGGGGGAGAAGGGCCTTGGTCAGGGAGGTGAT
AS_European_sdY             ----------------ATCGGGGGAGAAGGGCCTTGGTCAGGGAGGTGAT
AS_Tasmanian_Ssa06_sdY      ----------------ATCGGGGGAGAAGGGCCTTGGTCAGGGAGGTGAT
Chinook_sdY                 ATATTTATTTTTTATTATCAGGGGAGAAGGGCCTTGGTCAGGGAGGTGAC
RT_sdY                      ATATTTATTTTTTATTATCAGGGGAGAAGGGCCTTGGTCAGGGAGGTGAC
                                            *** ******************** ********
```

```
BT_sdY                    CAAGAACCCGATTGTCAATCGGACAGAGCTCCAGAGTTCCTCTGTGGAGA
AS_Tasmanian_Ssa02_sdY    CAAGAACCCGATTGTCAATCGGACAGAGCTCCAGAGTTCCTCTGTGGAGA
AS_Tasmanian_Ssa03_sdY    CAAGAACCCGATTGTCAATCGGACAGAGCTCCAGAGTTCCTCTGTGGAGA
AS_European_sdY           CAAGAACCCGATTGTCAATCGGACAGAGCTCCAGAGTTCCTCTGTGGAGA
AS_Tasmanian_Ssa06_sdY    CAAGAACCCGATTGTCAATCGGACAGAGCTCCAGAGTTCCTCTGTGGAGA
Chinook_sdY               CAATAACCTGATGGTCACTCTGACATAGCTCCAAAGTTCCTCTGTGGAAA
RT_sdY                    CAATAACCTGATGGTC-CTCTGACATAGCTCCAAAGTTCCTCTGTGGAGA
                          *** **** *** ***  ** **** ******* ************** *

BT_sdY                    TGGGAGAACCTTCCAGAAGGACAACCATCTCTGCAGCACTCCACCAATCA
AS_Tasmanian_Ssa02_sdY    TGGGAGAACCTTCCAGAAGGACAACCATCTCTGCAGCACTCCACCAATCA
AS_Tasmanian_Ssa03_sdY    TGGGAGAACCTTCCAGAAGGACAACCATCTCTGCAGCACTCCACCAATCA
AS_European_sdY           TGGGAGAACCTTCCAGAAGGACAACCATCTCTGCAGCACTCCACCAATCA
AS_Tasmanian_Ssa06_sdY    TGGGAGAACCTTCCAGAAGGACAACCATCTCTGCAGCACTCCACCAATCA
Chinook_sdY               TGGGAGAATCTTCCAGAATGACAGCCATCTCTGCAACACTCCAATAATCA
RT_sdY                    TGGGAGAATCTTCCAGAATGACAGCCATCTCTGCAGCACTCCACTAATCA
                          ******** ********* **** *********** *******  *****

BT_sdY                    GGCCTTTATGGTAGAGTGTCCAGACGGAAGCCACTCCTCAGTAAAAGGCA
AS_Tasmanian_Ssa02_sdY    GGCCTTCATGGTAGAGTGTCCAGACGGGAGCCACTCCTCAGTAAAAGGCA
AS_Tasmanian_Ssa03_sdY    GGCCTTCATGGTAGAGTGTCCAGACGGGAGCCACTCCTCAGTAAAAGGCA
AS_European_sdY           GGCCTTCATGGTAGAGTGTCCAGACGGGAGCCACTCCTCAGTAAAAGGCA
AS_Tasmanian_Ssa06_sdY    GGCCTTCATGGTAGAGTGTCCAGACGGGAGCCACTCCTCAGTAAAAGGCA
Chinook_sdY               GGCCTTTATAGTAGAGTGGCCAGACGGAAGCCACTCCTCAGTAAAAGGCA
RT_sdY                    GGCCTTTTTGGTAGAGTGGCCAGACGGAAGCCACTCCTCAGTAAAAGGCA
                          ******  * ******** ******** **********************
```

```
BT_sdY                    CATGACAGCCCGCTTGTAGTTTGCCAAAAGGAACCTAAAGGACTCTCAGA
AS_Tasmanian_Ssa02_sdY    CATGACAGCCCTCTTGTAGTTTGCCAAAAGGAACCTAAAGGACTCTCAGA
AS_Tasmanian_Ssa03_sdY    CATGACAGCCCGCTTGTAGTTTGCCAAAAGGAATCTAAAGGACTCTCAGA
AS_European_sdY           CATGACAGCCCGCTTGTAGTTTGCCAAAAGGAACCTAAAGGACTCTCAGA
AS_Tasmanian_Ssa06_sdY    CATGACAGCCCTCTTGTAGTTTGCCAAAAGGAACCTAAAGGACTCTCAGA
Chinook_sdY               CATGACAGCCCACTTGGAGTTTGCCAAAAGGAACATAAAGCACTCTCAGA
RT_sdY                    CATGACAGCCCACTTGGAGTTTGCCAAAAGGAACATAAAGCACTCTCAGA
                          *********** **** ****************  ***** *********

BT_sdY                    CCATGAGAAACAAGATTCTCTTGTCTGAATAAACCAAGATTGAACACTTT
AS_Tasmanian_Ssa02_sdY    CCATGAGAAACAAGATTCTCTTGTCTGAATAAACCAAGATTGAACACTTT
AS_Tasmanian_Ssa03_sdY    CCATGAGAAACAAGATTCTCTTGTCTGAATAAACCAAGATTGAACACTTT
AS_European_sdY           CCATGAGAAACAAGATTCTCTTGTCTGAATAAACCAAGATTGAACACTTT
AS_Tasmanian_Ssa06_sdY    CCATGAGAAACAAGATTCTCTTGTCTGAATAAACCAAGATTGAACACTTT
Chinook_sdY               CCATGAGAAACAAGATTCTCTGCTCTGATTTAATCAAGATTTAACTCTAT
RT_sdY                    CCATGAGAAACAAGATTCTCTGCTCTGATTAAATCAAGATTTAACTCTAT
                          *********************  ***** * ** ******* *** ** *

BT_sdY                    GGCCT-GAATGCCAAGTGTCACATCTGGAGGAAAGCTGGCACCATCCCTA
AS_Tasmanian_Ssa02_sdY    GGCCT-GAATGCCAAGTGACACATCTGGAGGAAAGCTGGCACCATCCCTA
AS_Tasmanian_Ssa03_sdY    GGCCT-GAATGCCAAGTGTCACATCTGGAGGAAAGCTGGCACCATCCCTA
AS_European_sdY           GGCCT-GAATGCCAAGTGTCACATCTGGAGGAAAGCTGGCACCATCCCTA
AS_Tasmanian_Ssa06_sdY    GGCCT-GAATGCCAAGTGTCACATCTGGAGGAAAGCTGGCACCATCCCTA
Chinook_sdY               GGCCT-AAATTACAAGCGTCACATCTGGTGGAAAGCTGGCACCATCCCTG
RT_sdY                    GGCCTAAAATTACAAGCGTCACATCTGGTGGAAAGCTGGCACCATCCCTA
                          *****  ***  **** * ********* ******************** 
```

```
BT_sdY                      CAGTGAAGCATGGTGGGAGCAGCATCATGCTGTGGGGATGTTTTTCAGCG
AS_Tasmanian_Ssa02_sdY      CGGTGAAGCATGGTGGGGGCAGCATCATGCTGTGGGGATGTTTTTCAGCG
AS_Tasmanian_Ssa03_sdY      CAGTGAAGCATGGTGGGGGCAGCATCATGCTGCGGGGATGTTTTTCAGCG
AS_European_sdY             CAGTGAAGCATGGTGGGGGCAGCATCATGCTGTGGGGATGTTTTTCAGCG
AS_Tasmanian_Ssa06_sdY      CGGTGAAGCATGGTGGGGGCAGCATCATGCTGTGGGGATGTTTTTCAGCG
Chinook_sdY                 TGA-----------------------------------------------
RT_sdY                      TGA-----------------------------------------------


BT_sdY                      GCAGGGACTGGGATCGAGGGAAAGATTAACAGAGCAAAGTACAGAGAGAT
AS_Tasmanian_Ssa02_sdY      GCAGGGACTGGGATCGAGGGAAAGATTAACAGAGCAAAGTACAGAGAGAT
AS_Tasmanian_Ssa03_sdY      GCAGGGACTGGGATCGAGGGAAAGATTAACAGAGCAAAGTACGGAGAGAT
AS_European_sdY             GCAGGGACTGGGATCGAGGGAAAGATTAACAGAGCAAAGTACAGAGAGAT
AS_Tasmanian_Ssa06_sdY      GCAGGGACTGGGATCGAGGGAAAGATTAACAGAGCAAAGTACAGAGAGAT
Chinook_sdY                 --------------------------------------------------
RT_sdY                      --------------------------------------------------


BT_sdY                      TCTTGATCCAGAGCGCTCAGGACCTCAGACTGGGGCTAAGGTTCACCTTC
AS_Tasmanian_Ssa02_sdY      TCTTGATCCAGAGCGCTCAGGACCTCAGACTGGGGCTAAGGTTCACCTTC
AS_Tasmanian_Ssa03_sdY      TCTTGATCCAGAGCGCTCAGGACCTCAGACTGGGGCTAAGGTTTACCTTC
AS_European_sdY             TCTTGATCCAGAGCGCTCAGGACCTCAGACTGGGGCTAAGGTTCACCTTC
AS_Tasmanian_Ssa06_sdY      TCTTGATCCAGAGCGCTCAGGACCTCAGACTGGGGCTAAGGTTCACCTTC
Chinook_sdY                 --------------------------------------------------
RT_sdY                      --------------------------------------------------
```

```
BT_sdY                      CAACAGGACAACGACCGTAAACACACAGCCAAGAAAACGCAGGAGTGGCT
AS_Tasmanian_Ssa02_sdY      CAACAGGACAACGACCGTAAACACACAGCCAAGACAACGCAGGAGTGGCT
AS_Tasmanian_Ssa03_sdY      CAACAGGACGACGACCGTAAACACACAGCCAAGACAACGCAGGAGTGGCT
AS_European_sdY             CAACAGGACAACGACCGTAAACACACAGCCAAGACAACGCAGGAGTGGCT
AS_Tasmanian_Ssa06_sdY      CAACAGGACAACGACCGTAAACACACAGCCAAGACAACGCAGGAGTGGCT
Chinook_sdY                 --------------------------------------------------
RT_sdY                      --------------------------------------------------

BT_sdY                      TCGGGACAAGTCTCTGAATTTCATTCACGAGCACGGACTTGAACCCGATC
AS_Tasmanian_Ssa02_sdY      TCAGGACAAGTCTCTGAATTTCATTCATGAGCACGGACTTGAACCCGATC
AS_Tasmanian_Ssa03_sdY      TCAGGACAAGTCTCTGAATTTCATTCATGAGCACGGACTTGAACCCGATC
AS_European_sdY             TCAGGACAAGTCTCTGAATTTCATTCATGAGCACGGACTTGAACCCGATC
AS_Tasmanian_Ssa06_sdY      TCAGGACAAGTCTCTGAATTTCATTCATGAGCACGGACTTGAACCCGATC
Chinook_sdY                 --------------------------------------------------
RT_sdY                      --------------------------------------------------

BT_sdY                      TAACATCTCTGTAGAGACCTGAAAATAGCTGTGCAGCGACATTTCCCATC
AS_Tasmanian_Ssa02_sdY      TAACATCTCTGTAGAGACCTGAAAATAGCTGTGCAGCGACATTTCCCATT
AS_Tasmanian_Ssa03_sdY      TAACATCTCTGTAGAGACCTGAAAATAGCTGTGCAGCGACATTTCCCATT
AS_European_sdY             TAACATCTCTGTAGAGACCTGAAAATAGCTGTGCAGCGACATTTCCCATT
AS_Tasmanian_Ssa06_sdY      TAACATCTCTGTAGAGACCTGAAAATAGCTGTGCAGCGACATTTCCCATT
Chinook_sdY                 --------------------------------------------------
RT_sdY                      --------------------------------------------------
```

```
BT_sdY                      CAACCTGACAGAGCTTGAGAGGATCTGCAGAGAATAATGAGAGAAACTCC
AS_Tasmanian_Ssa02_sdY      CAACCTGACAGAGCTTGAGAGGATCTGCAGAGAAGAATGAGAGAAACTCC
AS_Tasmanian_Ssa03_sdY      CAACCTGACAGAGCTTGAGAGGATCTGCAGAGAAGAATGAGAGAAACTCC
AS_European_sdY             CAACCTGACAGAGCTTGAGAGGATCTGCAGAGAAGAATGAGAGAAACTCC
AS_Tasmanian_Ssa06_sdY      CAACCTGACAGAGCTTGAGAGGATCTGCAGAGAAGAATGAGAGAAACTCC
Chinook_sdY                 --------------------------------------------------
RT_sdY                      --------------------------------------------------


BT_sdY                      CCAAATACAGGTGTGCCAAGCTTGTAGCGTCATACCCAAGAAGACTCGAT
AS_Tasmanian_Ssa02_sdY      CCAAATACAGGTGTGCCAAGCTTGTAGCGTCATACCCAAGAAGACTTGAT
AS_Tasmanian_Ssa03_sdY      CCAAATACAGGTGTGCCAAGCTTGTAGCGTCATACCCAAGAAGACTTGAT
AS_European_sdY             CCAAATACAGGTGTGCCAAGCTTGTAGCGTCATACCCAAGAAGACTTGAT
AS_Tasmanian_Ssa06_sdY      CCAAATACAGGTGTGCCAAGCTTGTAGCGTCATACCCAAGAAGACTTGAT
Chinook_sdY                 --------------------------------------------------
RT_sdY                      --------------------------------------------------


BT_sdY                      GCTGTAATCGCTGCCAAAGGTGTTTCAACAAAGTACTGAGTAAAGGGTCT
AS_Tasmanian_Ssa02_sdY      GCTGTAATCGCTGCCAAAGGTGCTTCAACAAAGTACTGAGTAAAGGGTCT
AS_Tasmanian_Ssa03_sdY      GCTGTAATCGCTGCCAAAGGTGCTTCAACAAAGTACTGAGTAAAGGGTCT
AS_European_sdY             GCTGTAATCGCTGCCAAAGGTGCTTCAACAAAGTACTGAGTAAAGGGTCT
AS_Tasmanian_Ssa06_sdY      GCTGTAATCGCTGCCAAAGGTGCTTCAACAAAGTACTGAGTAAAGGGTCT
Chinook_sdY                 -------------------------------------------------T
RT_sdY                      -------------------------------------------------T
                                                                             *
```

```
BT_sdY                      GAATACTTATCTAAATGTGATATTTC---------CGTTTTAA----AT-
AS_Tasmanian_Ssa02_sdY      GAATACTTATCTAAATGTGATATTTC---------CGTTTTAA----AT-
AS_Tasmanian_Ssa03_sdY      GAATACTTATCTAAATGTGATATTTC---------CGTTTAAA----AT-
AS_European_sdY             GAATACTTATCTAAATGTGATATTTC---------CGTTTAAA----AT-
AS_Tasmanian_Ssa06_sdY      GAATACTTATCTAAATGTGATATTTC---------CGTTTTAA----AT-
Chinook_sdY                 GAATACTCATGTAAATGTAATATTTCAATTTTTTTATTTCAATTTTTTT-
RT_sdY                      GAATACTCATGTAAATGTAATATTTCAAAAACAAATATTTCAA----ATG
                            ******* ** ******* *******           *** **     *

BT_sdY                      TTGTAAT--GAATTGGCTAATATTTCTAAAAACCTGTTTTTGCTTTGTCA
AS_Tasmanian_Ssa02_sdY      TTGTAAT--GAATTGGCTAATATTTCTAAAAACCTGTTTTTGCTTTGTCA
AS_Tasmanian_Ssa03_sdY      TTGTAAT--GAATTGGCTAATATTTCTAAAAACCTGTTTTTGCTTTGTCA
AS_European_sdY             TTGTAAT--GAATTGGCTAATATTTCTAAAAACCTGTTTTTGCTTTGTCA
AS_Tasmanian_Ssa06_sdY      TTGTAAT--GAATTGGCTAATATTTCTAAAAACCTGTTTTTGCTTTGTCA
Chinook_sdY                 TTTAAAT--AAATTGGCTAATATTTCTACACACCTGTTTTTTCTTTGTCA
RT_sdY                      TTTTAATAGAAATTGGCTAATATTTCTACACACCTGTTTTTTCTTTGTCA
                            **  ***   ****************** * ********** ********

BT_sdY                      TTATGGGGTATTGTGTGTAGAATAATGA-GGGAAAAAACTATTTAATCCA
AS_Tasmanian_Ssa02_sdY      TTATGGGGTA-TGTGTGTAGATTAATGAGGGGAAAAAACTATTTAATCCA
AS_Tasmanian_Ssa03_sdY      TTATGGGGTA-TGTGTGTAGATTAATGAGGGGAAAAAACAATTTAATCCA
AS_European_sdY             TTATGGGGTA-TGTGTGTAGATTAATGAGGGGAAAAAACAATTTAATCCA
AS_Tasmanian_Ssa06_sdY      TTATGGGGTA-TGTGTGTAGATTAATGAGGGGAAAAAACTATTTAATCCA
Chinook_sdY                 TTGTGGGGTATTGTGTGTAGATTAATGA-GGGAAAAAGCAATTTAATCAA
RT_sdY                      TTGTGGGGTATTGTGTGTAGATTAATGAGGGGAAAAGA-AATTTAATCAA
                            ** ******* ********** ****** *******    ******** *
```

```
BT_sdY                      TTTTAGAATAAGGCTATAATGTAACAAAACGTGGAAAAAGTCAAGGGGTC
AS_Tasmanian_Ssa02_sdY      TTTTAGAATAAGGCTATAATGTAACAAAACGTGGAAAAAGTCGAGGTGTC
AS_Tasmanian_Ssa03_sdY      TTTTAGAATAAGGCTATAATGTAACAAAACGTGGAAAAAGTCAAGGTGTC
AS_European_sdY             TTTTAGAATAAGGCTATAATGTAACAAAACGTGGAAAAAGTCAAGGTGTC
AS_Tasmanian_Ssa06_sdY      TTTTAGAATAAGGCTATAATGTAACAAAACGTGGAAAAAGTCAAGGTGTC
Chinook_sdY                 TTTTGGAATAATGCTGAAATGTAACACAATTTGGAAAATGTCAAGGAG--
RT_sdY                      TTTTGGAATAATGCTGAAATGTAACACAATTTGGAAAATGTCAAGGAGAC
                            **** ****** ***  ********* **  ******* *** *** *

BT_sdY                      TGAATCCTTTCCCAATGCACTGTATATGTGTGAATGTGTATGAGGCATTA
AS_Tasmanian_Ssa02_sdY      TGAATACTTTCCCAATGCACTGTATATGTGTGAATGTGTATGAGGCGTTA
AS_Tasmanian_Ssa03_sdY      TGAATACTTTCCCAATGCACTGTATATGTGTGAATGTGTATGAGGCGTTA
AS_European_sdY             TGAATACTTTCCCAATGCACTGTATATGTGTGAATGTGTATGAGGCGTTA
AS_Tasmanian_Ssa06_sdY      TGAATACTTTCCCAATGCACTGTATATGTGTGAATGTGTATGAGGCGTTA
Chinook_sdY                 -----ACTTTCCCAATGC-----ATATGTGTAAATGTGTATGGGGCCTTA
RT_sdY                      TGAATACTTTCCCAATGC-----ATATGTGTAAACGTGTATGGGGCCTTA
                                  ************     ******** ** ******* *** ***

BT_sdY                      CAGA-----GCCACTTGATGTTGATTATAATTAGATGTT-AACAATCCTT
AS_Tasmanian_Ssa02_sdY      CAGA-----GCCACTTGATGTTGATTATAATTAGATGTT-CACAATCCTT
AS_Tasmanian_Ssa03_sdY      CAGA-----GCCACTTGATGTTGATTATAATTAGATGTT-CACAATCCTT
AS_European_sdY             CAGA-----GCCACTTGATGTTGATTATAATTAGATGTT-CACAATCCTT
AS_Tasmanian_Ssa06_sdY      CAGA-----GCCACTTGATGTTGATTATAATTAGATGTT-CACAATCCTT
Chinook_sdY                 CTTACTTACTCCACTTGGTGTTGATTAT---------------AATCTTT
RT_sdY                      CTTACTTACTCCACTTGGTGTTGATTATAATTAGATGTTGAACAATCTTT
                            *  *      ******* **********               **** **
```

```
                          |           exon4
BT_sdY                      CCTTCTCCAG|ATTAAATTGCCATGGGCTCAGCAGCTATTCAAGCAAGCTC
AS_Tasmanian_Ssa02_sdY      CCTTCTCCAG|ATTAAATTGCCATGGGCTCAGCAGCTATTCAAGCAAGCTC
AS_Tasmanian_Ssa03_sdY      CCTTCTCCAG|ATTAAATTGCCATGGGCTCAGCAGCTATTCAAGCAAGCTC
AS_European_sdY             CCTTCTCCAG|ATTAAATTGCCATGGGCTCAGCAGCTATTCAAGCAAGCTC
AS_Tasmanian_Ssa06_sdY      CCTTCTCCAG|ATTAAATTGCCATGGGCTCAGCAGCTATTCAAGCAAGCTC
Chinook_sdY                 CCTTCTCCAG|ATTAAATTGCCATGGGCTCAGCAGCTATTCAAGGAAGTTC
RT_sdY                      CCTTCTCCAG|ATTAAATTACCATGGGCCCAGCAGCTATTCAAGGAAGTTC
                            **********|******** ******** *************** *** **

                              exon4                       |
BT_sdY                      ACGACTTCAGGATCTGGCTTGAGTCCTCCCCTGTCTCTCCTGGAGTCTGA|
AS_Tasmanian_Ssa02_sdY      ACGACTTCAGGATCTGGCTTGAGTCCTCCCCTGTCTCTCCTGGAGTCTGA|
AS_Tasmanian_Ssa03_sdY      ACGACTTCAGGATCTGGCTTGAGTCCTCCCCTGTCTCTCCTGGAGTCTGA|
AS_European_sdY             ACGACTTCAGGATCTGGCTTGAGTCCTCCCCTGTCTCTCCTGGAGTCTGA|
AS_Tasmanian_Ssa06_sdY      ACGACTTCAGGATCTGGCTTGAGTCCTCCCCTGTCTCTCCTGGAGTCTGA|
Chinook_sdY                 ATGACTTCAGGATCTGGCTTGAGTCCTCCCCTGTCTCTCCTGGAGTCTGA|
RT_sdY                      ATGACTTCAGGATCTGGCTTGAGTCCTCCCCTGTCTCTCCTGGAGTCTGA|
                            * ************************************************|

BT_sdY                      AAATATGGTTG--GTCCCAACAGGACTCCAATTGCTCCCAATATTAGCCT
AS_Tasmanian_Ssa02_sdY      AAATATGGTTG--GTCCCAACAGGACTCCAATTGCTCCCAATATTAGCCT
AS_Tasmanian_Ssa03_sdY      AAATATGGTTG--GTCCCAACAGGACTCCAATTGCTCCCAATATTAGCCT
AS_European_sdY             AAATATGGTTG--GTCCCAACAGGACTCCAATTGCTCCCAATATTAGCCT
AS_Tasmanian_Ssa06_sdY      AAATATGGTTG--GTCCCAACAGGACTCCAATTGCTCCCAATATTAGCCT
Chinook_sdY                 AGAACCGGTTGGAGTCCCAACAGGA---------CTCCCAATATGAGCCT
RT_sdY                      AGAACTGGTTGGAGTCCCAACAGGACCCCAACTGCTCCCAATATGAGCCT
                            * *   *****  ************         ********** *****
```

```
BT_sdY                      ATGCCACAGCCCTTTCCACCACACCTGTTCCCACACCCTAATCACC-TCT
AS_Tasmanian_Ssa02_sdY      ATGCCACAGCCCTTTCCACCACACCTGTTCCCACACCCTAATCACC-TCT
AS_Tasmanian_Ssa03_sdY      ATGCCACAGCCCTTTCCACCACACCTGTTCCCACACCCTAATCACC-TCT
AS_European_sdY             ATGCCACAGCCCTTTCCACCACACCTGTTCCCACACCCTAATCACC-TCT
AS_Tasmanian_Ssa06_sdY      ATGCCACAGCCCTTTCCACCACACCTGTTCCCACACCCTAATCACC-TCT
Chinook_sdY                 AGGCCACAGCCTTTCCCACCAAACTTGT------ACCCTAATCACC-TCT
RT_sdY                      AGGCCACAGCCTTTCCCACCAAACCTCT------ACCCTAATCACCTTCT
                            * ********* ** ****** ** * *      ************ ***

BT_sdY                      CCTCCACCTTTTTACCTCTTTTCATTCCCTATGTAAGCAGCATGCTTCTT
AS_Tasmanian_Ssa02_sdY      CCTCCACCTTTTTACCTCTTTTCATTCCCTATATAAGCAGTATGCTTCTT
AS_Tasmanian_Ssa03_sdY      CCTCCACCTTTTTACCTCTTTTCATTCCCTATATAAGCAGTATGCTTCTT
AS_European_sdY             CCTCCACCTTTTTACCTCTTTTCATTCCCTATATAAGCAGTATGCTTCTT
AS_Tasmanian_Ssa06_sdY      CCTCCACCTTTTTACCTCTTTTCATTCCCTATATAAGCAGTATGCTTCTT
Chinook_sdY                 CCTCAACCTTTTCACCTATTTTCATTCCCTATGTAAACAG-------CTT
RT_sdY                      CCTAAACCTTTTCACCTATTTTCATTCCCTATATAAACAG-------CTT
                            ***  ******* **** ************** *** ***       ***

                                                                          <---
BT_sdY                      CCTGAGCTATAGTA-TGGCAC-----------------------------
AS_Tasmanian_Ssa02_sdY      CCTGAGCTATAGTATTGGCACTAT--------------------------
AS_Tasmanian_Ssa03_sdY      CCTGAGCTATAGTATTGGCACTAT--------------------------
AS_European_sdY             CCTGAGCTATAGTATTGGCACTATATTTCTTCCACCTCACACTAAGTAGA
AS_Tasmanian_Ssa06_sdY      CCTGAGCTATAGTATTGGCACTAT--------------------------
Chinook_sdY                 ATTGA------------GCACTATATTTATTCCATCTCACACTAAATAGA
RT_sdY                      ATTGA------------GCACTATATTTATTCCATCTCACACTAAATAGA
                              ***            ****
```

K. P. Lubieniecki *et al.*

```
                           --sdY 3'UTR--------
BT_sdY                     --------------------------------------------------
AS_Tasmanian_Ssa02_sdY     --------------------------------------------------
AS_Tasmanian_Ssa03_sdY     --------------------------------------------------
AS_European_sdY            AGACAATCAGCCAGTAAATAAA----------------------------
AS_Tasmanian_Ssa06_sdY     --------------------------------------------------
Chinook_sdY                AGACATTCAGACAGTAAATTAATAAATACCCTTCAAATACATAATTTTAA
RT_sdY                     AGACAATCAGACAGTAAATTAATAAATACCCTTCA---------------


BT_sdY                     --------------------------------------------------
AS_Tasmanian_Ssa02_sdY     --------------------------------------------------
AS_Tasmanian_Ssa03_sdY     --------------------------------------------------
AS_European_sdY            --------------------------------------------------
AS_Tasmanian_Ssa06_sdY     --------------------------------------------------
Chinook_sdY                TGTTGCTTTTTTGTCCATTTCAGATTAATGCTTACTTCATGCTGTTTGTT
RT_sdY                     --------------------------------------------------


BT_sdY                     ------------
AS_Tasmanian_Ssa02_sdY     ------------
AS_Tasmanian_Ssa03_sdY     ------------
AS_European_sdY            ------------
AS_Tasmanian_Ssa06_sdY     ------------
Chinook_sdY                CTGTTACAGTTG
RT_sdY                     ------------
```

Figure S1 CLUSTAL 2.1 multiple sequence alignment of sdY from brown trout (BT), three Tasmanian and one European Atlantic salmon, Chinook salmon and rainbow trout (RT). The boundaries of exons and locations of primers are shown.
