# Supplementary material for: Genomic Instability of the Sex-Determining Locus in Atlantic Salmon (Salmo salar)
Source: G3 (Bethesda). 2015 Sep 22;5(11):2513–22. doi: 10.1534/g3.115.020115 (PMC4632069; doi:10.1534/g3.115.020115)
Supplement: Supporting Information [file supp_g3.115.020115_FigureS2.pdf]

```
sdY                    MADREARFQAQHSFLVSVEYCEEEVLSHEVMGSDVRIAYKPFSLMMDGIPVISLPKPPDT
AS_Tasmanian_Ssa02     MVDREARFQAQHSFLVSVEYCEEEVLSHEVMGSDVRIAYKPFSLMMDGIPVISLPKPPDT
AS_Tasmanian_Ssa03     MVDREARFQAQHSFLVSVEYCEEEVLSHEVMGSDVRIAYKPFSLMMDGIPVISLPKPPDT
AS_European            MVDREARFQAQHSFLVSVEYCEEEVLSHEVMGSDVRIAYKPFSLMMDGIPVISLPKPPDT
AS_Tasmanian_Ssa06     MVDREARFQAQHSFLVSVEYCEEEVLSHEVMGSDVRIAYKPFSLMMDGIPVISLPKPPDT
Chinook_sdY            MADREARIQAQHCFLVSVEYCEEEVLSHEVMGGDVRIAHKT-SLMMDGIPFISLPKPPNT
RT_sdY                 MADREARIQAQHCFLVSVEYCEEEVLSHEVMGGDVRIAHKT-SLMMDGIPFISLPKPPNT
                       *.*****:****.*******************.*****:*. ********.*******:*

BT_sdY                 IPISSDRSTLSNLLSLMEGGVVLSSREEGIYAERHSQAIVSWMGGTGDEMHVMERDVDPV
AS_Tasmanian_Ssa02     IPISSDRSILSNLLSLMEGGVVLSSKEEGIYAERHSQAIVSWMGGTGDEMHVMERDVDPV
AS_Tasmanian_Ssa03     IPISSDRSILSNLLSLMEGGVVLSSKEEGIYAERHSQAIVSWMGGTGDEMHVMERDVDPV
AS_European            IPISSDRSILSNLLSLMEGGVVLSSKEEGIYAERHSQAIVSWMGGTGDEMHVMERDVDPV
AS_Tasmanian_Ssa06     IPISSDRSILSNLLSLMEGGVVLSSKEEGIYAERHSQAIVSWMGGTGDEMHVMERDVDPV
Chinook_sdY            LPISSDRSILSNLLSLMEGGVVLSSREEGIYAERHSQATVSWMGGTGDEMHVMDRDVDPV
RT_sdY                 LPISSDRSILSNLLSLMEGGVVLSSREEGIYAERHSQATVSWMGGTGDEMHVMERDVDPV
                       :******* ****************:************ **************:******

BT_sdY                 MLFNRETFRQELERFSRADGFQPQIGFSLWFGQDSSLSAPISISIKLPWAQQLFKQAHDF
AS_Tasmanian_Ssa02     MLFNRETFRQELERFSRADGFQPQIGFSLWFGQDSSLSAPISISIKLPWAQQLFKQAHDF
AS_Tasmanian_Ssa03     MLFNRETFRQELERFSRADGFQPQIGFSLWFGQDSSLSAPISISIKLPWAQQLFKQAHDF
AS_European            MLFNRETFRQELERFSRADGFQPQIGFSLWFGQDSSLSAPISISIKLPWAQQLFKQAHDF
AS_Tasmanian_Ssa06     MLFNRETFRQELERFSRADGFQPQIGFSLWFGQDSSLSAPISISIKLPWAQQLFKQAHDF
Chinook_sdY            MLFNRETFRQELERFARADGSQPQCGFSLWFGQDSSLSAPNFISIKLPWAQQLFKEVHDF
RT_sdY                 MLFNREHFRQELDRFARADGSQPQCGFSLWFGQDSSLSAPIFISIKLPWAQQLFKEVHDF
                       ****** *****:**:**** *** ***************  *************:.***
```

```
BT_sdY                  RIWLESSPVSPGV
AS_Tasmanian_Ssa02      RIWLESSPVSPGV
AS_Tasmanian_Ssa03      RIWLESSPVSPGV
AS_European             RIWLESSPVSPGV
AS_Tasmanian_Ssa06      RIWLESSPVSPGV
Chinook_sdY             RIWLESSPVSPGV
RT_sdY                  RIWLESSPVSPGV
                        *************
```

Figure S2 CLUSTAL 2.1 multiple sequence alignment of sdY from brown trout (BT), three Tasmanian and one European Atlantic salmon, Chinook Ssalmon and rainbow trout (RT).
