# Supplementary material for: Genomic Instability of the Sex-Determining Locus in Atlantic Salmon (Salmo salar)
Source: G3 (Bethesda). 2015 Sep 22;5(11):2513–22. doi: 10.1534/g3.115.020115 (PMC4632069; doi:10.1534/g3.115.020115)
Supplement: Supporting Information [file supp_g3.115.020115_FigureS3.pdf]

```
Atlantic Salmon Ctg_2    TTGT-TCTTAG--GCAAC------------GAATGG-AT-------CATTTCTAGTGA--
Rainbow Trout            TTGTATTTTAACAGCAACAGTTCCAACCTAGACAGGTATATAAGGGTGTTTTTAATGAAA
Chinook Salmon           TTGTATTTTAACAGCAACAGTTCCAACCTAGACAGGTATATAAGGGTGTTTTTAATGAAA
                         **** * ***   *****            **  ** **         *** ** ***

Atlantic Salmon Ctg_2    ---TATT-CAGAGCAT------GGCTGTGATAAATTACATGTGGGTGCATTAGGAAACAG
Rainbow Trout            ATATATTTCAAGGTATTTCATTGTTTGTGTTGCATTTCTTG--GGGGCATAAGGGCAATG
Chinook Salmon           ATATATTTCAAGGTATTTCACTGTTTGTGTTGCATTTCTT--GGGGGCATAAGGGCAATG
                            **** **  * **      *  **** *   **   *   ** **** ***  *  *

Atlantic Salmon Ctg_2    AGGGTGCACCCAAATGGA----------------ACCCTATTC--GATATATAGTGCACT
Rainbow Trout            AAA-TGTACCAATATTGACATATAGTTGCACATTTTCCTACGCTTGGCTCCCAGGAAAAC
Chinook Salmon           AAAACGTACCAATATTGACATATAGTTGCATATTTTCCTACGCTTGGCTCCCAGGAAAAC
                         *    * *** *  * **                  ****  *  *      **   *

Atlantic Salmon Ctg_2    ATTTTTGGTTTA------------CAAAGAATTATAT--------TGTAGTGTCTTCTGT
Rainbow Trout            ATTTCTCATTTGGGTCCTAGGCTGAAAAAGTTTAAGAACCC----TGTAGTGTCTTCTGT
Chinook Salmon           ATTTCTCATTTGGGTCCTAGGCTGAAAAAGTTTAAGAACCCCTGCTGTAGTGTTTTCTGT
                         **** *  ***              ***   ***           ******** ******

Atlantic Salmon Ctg_2    CTTTATCCTGTCTGGTACAAGATAAAGTCTGCAAAGAGGAGGAGAAGGGAGAGAGAGTGA
Rainbow Trout            CTTTATCCTCCCTGGTACAAGATCAAGTCTGGAGAGAGGAG---AAGGGAGAGAG--TGA
Chinook Salmon           CTTTATCCTCCCTGGTACAAGATCAAGTCTGGAGAGTGGAG---AAGGGAGAGAGAGTGA
                         *********  ************ ******* * ** ****   ***********  ***

Atlantic Salmon Ctg_2    GCAAGGCCTTGGCCTTCACCTGACAAGAGAGGATTGAGAGAGAAACACTCCCCTTAACTA
Rainbow Trout            GCAAGGCCTTGGCCTTCACCTGACAAGAGAGGATTGAGAGAGAAACACTCCCCTTAACTA
Chinook Salmon           GCAAGGCCTTGGCCTTCACCTGACAAGAGAGGATTGAGAGAGAAACACTCCCCTTAACTA
                         ************************************************************

Atlantic Salmon Ctg_2    ATCATTTTACATGAAAAAATGCATATCAAAAACTCCAGCGCATGTTTAAAAACAGTCCTT
Rainbow Trout            ATACTTTTACATGAAAACATGCTCATAAAAAACTCCAGCTCATGTTTAAAAGCCGTCCTT
Chinook Salmon           ATACTTTTACATGAAAACATGCACATAAAAAACTCCAGCTCATGTTTAAAAGCCGTCCTT
                         **  ************* ****  ** ************ *********** * ******

                                                    |Exon 1
                                                    ATGGCTGACAGAGAGGCCAGAATCCAAG
Atlantic Salmon Ctg_2    AGAGGTTTTGAATCCACCTGCCCTTCAATGGTTGACAGAGAGGCCAGATTCCAAGGTACA
Rainbow Trout            AGAATATTTGAGTCCATCTGCCCTTCAATGGCTGACAGAGAGGCCAGAATCCAAGGTACA
Chinook Salmon           AGAATAGTTGAGTCCATCTGCCCTTCAATGGCTGACAGAGAGGCCAGAATCCAAGGTACA
                         ***   ***** ******************
```

**Figure S3** CLUSTAL multiple sequence alignment by Kalign (2.0) of 5’ sdY sequences of Atlantic salmon, rainbow trout and Chinook salmon.
