# Supplementary material for: Genomic Instability of the Sex-Determining Locus in Atlantic Salmon (Salmo salar)
Source: G3 (Bethesda). 2015 Sep 22;5(11):2513–22. doi: 10.1534/g3.115.020115 (PMC4632069; doi:10.1534/g3.115.020115)
Supplement: Supporting Information [file supp_g3.115.020115_TableS1.pdf]

Table S1 Nucleotide sequences of primers and probes.

Primer name	Sequence 5' - 3'	Purpose
SDY exon1 F	ATGGTTGACAGAGAGGCCAGA	
SDY exon 1 R	CCATCATCAGGGAGAAGGGC	
SDY exon2 F probe	TGATGGATGGGATCCCCGTCATCTCTCTCCCAAAG	
SDY exon2 R	TAGAGCTTAAAACCACTCCACCCTCCATGAGGGA	
SDY exon3 F	AGTTGGAACGCTTCAGCAGAGCAGATGG	
SDY exon3 R	AGATTGGTGCACTGAGTGATGAGTCTTGTCC	
SDY exon4 F probe	GACTTCAGGATCTGGCTTGAGTCCTCCCCTGTCTCTCCTGGAG	
SDY exon4 R	ATTTACTGGCTGATTGTCTTCTA	
Contig 2 FISH 858 F	TGTGGGTGCATTAGGAAACA	cloning for FISH plasmid
Contig 2 FISH 5976 R	GACTGTGGGGCTGAGACAAT	cloning for FISH plasmid
Contig 2 6155 F	GGTAGCACTTGTAGCCAGAC	male-female boundary check
Contig 2 6450 R	TGGTGACATGGTGAGTAGTG	male-female boundary check
Contig 2 8297 F	CTTAGAATGTTGGGTTGGAG	male-female boundary check
Contig 2 8473 R	CATTCACAGATCCCAGTGC	male-female boundary check
Contig 2 14711 F	GGCATGTTTGTACAAGAACC	male-female boundary check
Contig 2 15014 R	ACAGGTGATGAAGTGAAAGC	male-female boundary check
Contig 2 18353 F	GAATGCAGCTTTCTTACCTG	male-female boundary check
Contig 2 18680 R	ATATGGTATGACCCAACCAG	male-female boundary check
Contig 1 95417 F	ATTGAACAGGTTACCAGGTG	male-female boundary check
Contig 1 95712 R	TCCTCACTCACACAAGACAA	male-female boundary check
Contig 1 86142 F	GATGCTGGGTAAGTCTGAAG	male-female boundary check
Contig 1 86552 R	CCATTTCCTCCAGGTAACTA	male-female boundary check

Contig 1 42083 F	AGGGAACCGTTGATCTACTT	male-female boundary check
Contig 1 42446 R	TACTGCACATTCTGGTGAAA	male-female boundary check
Contig 1 12665 F	AGAAATGTGGAGCAAGAAGA	microsatellite marker
Contig 1 12947 R	TGTAAAACGACGGCCAGTTGTGTATGCGTTTACTGCTT	microsatellite marker
Exon 1 F	ATGGTTGACAGAGAGGCCAGA	sequencing primer
Exon 1 R	CCATCATCAGGGAGAAGGGC	sequencing primer
Exon 2 F	CCCAGCACTCTTTTCTTGTCTC	sequencing primer
Exon 2 R	CCTGTCTGAAGGTCTCCCTG	sequencing primer
Exon 3 R	AGATTGGTGCACTGAGTGATGAGTCTT	sequencing primer
Exon 3 F	AGTTGGAACHCTTCGCCAGAGCAGATGG	sequencing primer
Exon 3-4 F2a	AAATTCAATTTATGGGACATG	sequencing primer
sdY 3' UTR R (Exon 4 R)	ATTTACTGGCTGATTGTCTTCTA	sequencing primer
sdY 5' UTR F (Exon 1 F)	AGAGTGAGCAAGGCCTTGG	sequencing primer
Salmon & BT 10bp gap R	GTCCGCCTATGGAAATCAATTG	sequencing primer
sdY Exon 3-intron-end F2	TATGAACACAACCAGTCATGATG	sequencing primer
